# Accelerating Influenza Research: Vaccines, Antivirals, Immunomodulators and Monoclonal Antibodies. The Manufacture of a New Wild-Type H3N2 Virus for the Human Viral Challenge Model

**DOI:** 10.1371/journal.pone.0145902

**Published:** 2016-01-13

**Authors:** Daniel J. Fullen, Nicolas Noulin, Andrew Catchpole, Hosnieh Fathi, Edward J. Murray, Alex Mann, Kingsley Eze, Ganesh Balaratnam, Daryl W. Borley, Anthony Gilbert, Rob Lambkin-Williams

**Affiliations:** hVIVO Services Limited, Queen Mary BioEnterprises Innovation Centre, 42 New Rd, London, E1 2AX, England, United Kingdom; University of South Dakota, UNITED STATES

## Abstract

**Background:**

Influenza and its associated diseases are a major cause of morbidity and mortality. The United States Advisory Committee on Immunization Practices recommends influenza vaccination for everyone over 6 months of age. The failure of the flu vaccine in 2014–2015 demonstrates the need for a model that allows the rapid development of novel antivirals, universal/intra-seasonal vaccines, immunomodulators, monoclonal antibodies and other novel treatments. To this end we manufactured a new H3N2 influenza virus in compliance with Good Manufacturing Practice for use in the Human Viral Challenge Model.

**Methods and Strain Selection:**

We chose an H3N2 influenza subtype, rather than H1N1, given that this strain has the most substantial impact in terms of morbidity or mortality annually as described by the Centre for Disease Control. We first subjected the virus batch to rigorous adventitious agent testing, confirmed the virus to be wild-type by Sanger sequencing and determined the virus titres appropriate for human use via the established ferret model. We built on our previous experience with other H3N2 and H1N1 viruses to develop this unique model.

**Human Challenge and Conclusions:**

We conducted an initial safety and characterisation study in healthy adult volunteers, utilising our unique clinical quarantine facility in London, UK. In this study we demonstrated this new influenza (H3N2) challenge virus to be both safe and pathogenic with an appropriate level of disease in volunteers. Furthermore, by inoculating volunteers with a range of different inoculum titres, we established the minimum infectious titre required to achieve reproducible disease whilst ensuring a sensitive model that can be translated to design of subsequent field based studies.

**Trial Registration:**

ClinicalTrials.gov NCT02525055

## Introduction

Since Edward Jenner performed the first documented Human Viral Challenge (HVC) study with smallpox on the 14^th^ of May 1796[1], the utility of such studies has been apparent. In 1931 Sir Christopher Andrews returned from the US where he had observed the use of chimpanzees in the study of influenza. However, as his return coincided with the great depression, funding for similar work in the UK was extremely limited. Sir Christopher therefore decided to enrol students from St Bartholomew’s Hospital. He explained to them that as he could not get chimpanzees, he considered the next best thing would be a “Bart’s” student. Despite the comment that “they were cheaper than chimpanzees”, over 100 students immediately enrolled, but the students had to continue their studies and were not isolated in the same way the chimpanzees had been in the USA[2]. This confounded any analysis of the data as the investigators could not be certain that the symptoms were not due to any other respiratory viruses acquired in the community. The UK’s Medical Research Council (MRC) terminated the work just a year later.

After the conclusion of World War II, a new approach was pioneered by Dr David Tyrell at the Common Cold Institute (CCI). From 1946, volunteers were inoculated by instilling small quantities of virus into their noses. The CCI housed healthy volunteers in relative isolation from other people, thereby reducing the risk of contact with natural sources of infection or of passing on the virus to members of the public. During its time, the unit attracted 20,000 volunteers until its closure in 1989.

The HVC Model using healthy volunteers provides a unique opportunity to describe the viral lifecycle as: the time point of infection is known with certainty, nasal virus shedding can be measured, symptoms are recorded prospectively and participants are selected with low pre-haemagglutination inhibition (HAI) antibody titres to ensure a statistically significant infection rate with a relatively small number of volunteers.

Post 1989 experimental infection studies continued, with small motels and hotels in the USA and UK substituting for the wooden huts on Salisbury Plain. Such studies contributed to the significant development of the new neuraminidase inhibitors during the 1990s[3–13]

We restarted HVC studies in the UK in 2001 and since then we have conducted multiple studies with over 2000 volunteers inoculated with Influenza, Respiratory Syncytial Virus (RSV) or Human Rhinovirus (HRV), and multiple proof of concept studies[14–16].

Influenza and its associated diseases are a major cause of morbidity and mortality[17]. It is important to note that Influenza A (H3N2) causes the greatest morbidity and mortality on an annual basis[18] even when compared to the recent 2009 pandemic H1N1 strain[19], hence it is our focus for model development.

In this paper we describe the selection, production and characterisation of a new GMP H3N2 Wild-type influenza challenge virus for use in human challenge studies. This new strain was developed to replenish our H3N2 challenge stocks and to update our portfolio with a more recently circulating strain.

## Methods

**Fig 1:**
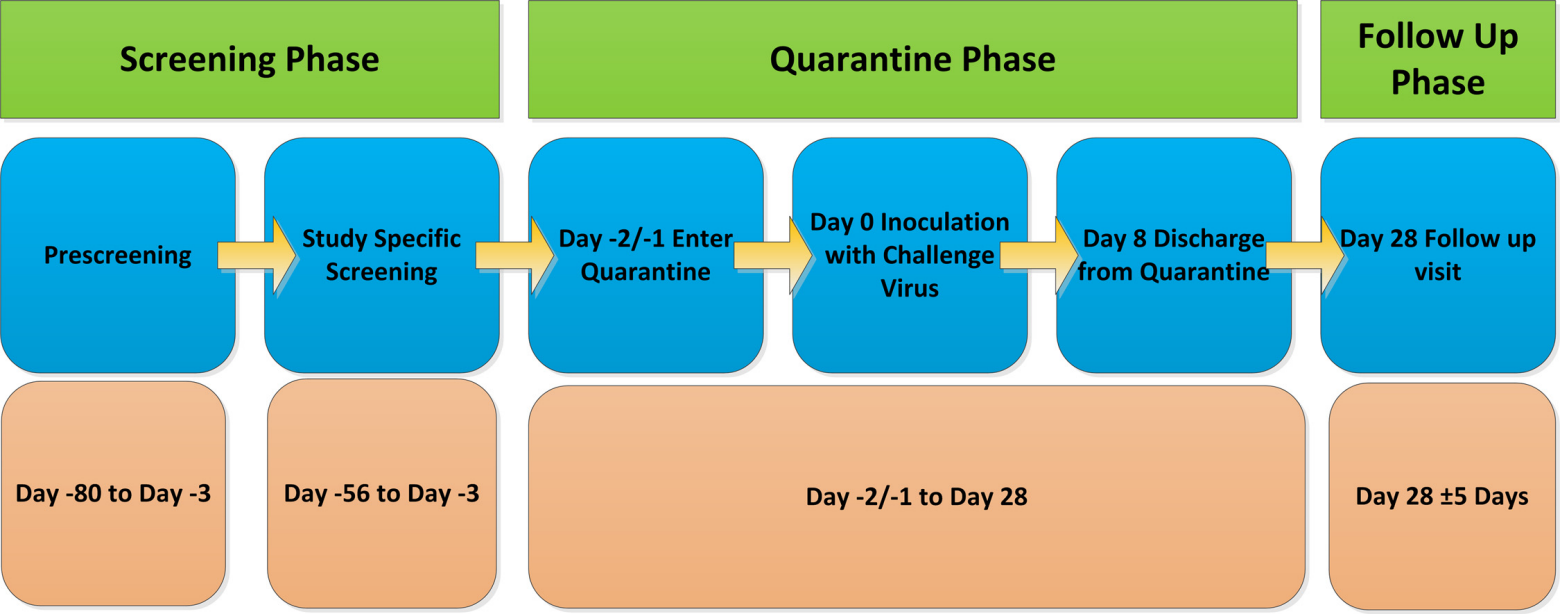
Summary of Clinical Trial Design.

The study was approved by City and East London NRES and the study conducted in accordance with Good Clinical Practice and the Declaration of Helsinki 1996. We thank the committee for their constructive input. A summary of the clinical study design in accordance with the Consort principles is shown in Fig 1. All volunteers provided full written consent using a form approved by the committee.

### Virus Strain Selection

A large number of viruses were considered for the challenge strain based on an extensive literature review, phylogenetic analysis and clinical relevance. A short list of twenty H1N1 and H3N2 viruses were obtained and screened against a panel of 245 volunteers’ sera using the HAI assay [20]. The results were used to identify viruses serosuitable for use in the model, defined as a value of <10 by the HAI assay.

Viruses that had a serosuitable rate of greater than 30% were selected for further analysis, the rationale being to ensure that a high proportion of the volunteer population would be susceptible to infection with the chosen strain. An extensive literature search was performed to confirm there was no known apparent adverse pathology for the selected viruses[21, 22].

Influenza A/Perth/16/2009 (H3N2) was a vaccine component for two influenza seasons (2010 to 2012) in the northern hemisphere it also had the highest serosuitable rate of the strains tested (S1 Fig). It is important to note variability between HAI assays may occur mainly due to the batch of turkey or chicken red blood cells used [23]. To compensate for this we ran up to four tests per volunteer.

### Challenge Virus Production

The virus seed material was reconstituted in water for injection (USP) grade water. Pilot studies were performed *in-vitro* to determine the lowest infectious titre that could be used for consistent recovery of virus at harvest.

**Fig 2:**
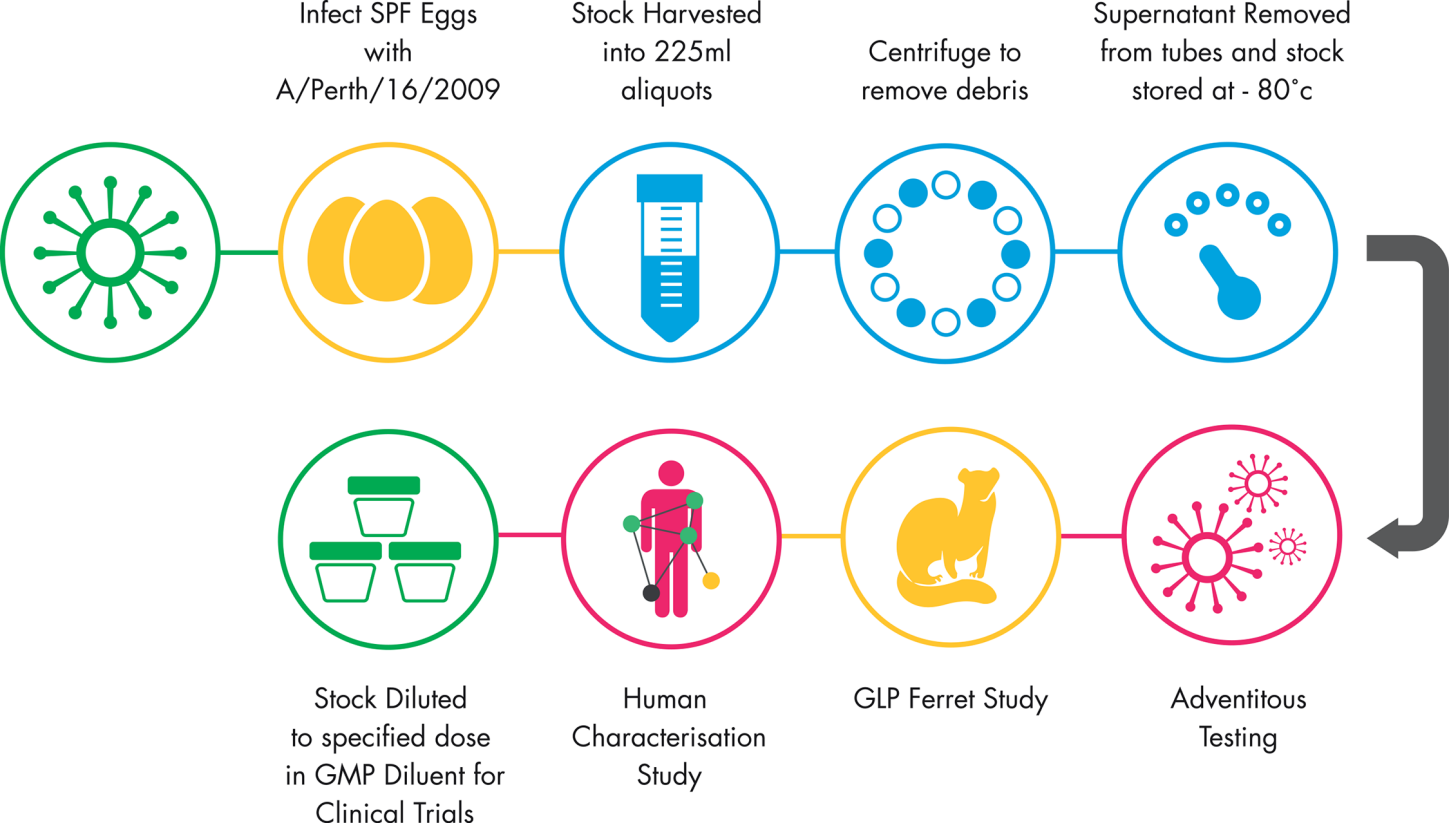
Summary of Production of the GMP Challenge Stock.

Specific Pathogen Free (SPF) eggs were inoculated with virus at a dilution of 1 x 10^−4^,100 μl per egg under Good Manufacturing Practice (GMP) conditions at Meridian Life Sciences (Memphis, USA). The eggs were incubated for 72 hours at 34°C, candled to remove non-viable eggs to reduce the risk of adventitious agents contaminating the pool, chilled overnight at 4°C and the allantoic fluid harvested. The allantoic fluid was centrifuged to remove large particulates and stored at -80°C. Identity of the virus was confirmed to be identical to wild-type virus by amino acid sequence alignment as confirmed by Sanger sequencing (Genbank Refs: ACS71642, AJK01457, AHX37629, AJU46087 and ADW80518) and no drug resistant mutations to oseltamivir and zanamivir were found. The virus was also confirmed to be phenotypically susceptible to oseltamivir and zanamivir in the neuraminidase inhibition assay (NAI). The infectious virus titre determined by tissue culture and expressed as Tissue Culture Infective Dose (TCID_50_). The virus was screened for potential adventitious agents. All results were satisfactory and confirmed the suitability of the Influenza A/Perth/16/2009 (H3N2) GMP stock for use in the HVC model. A summary of the production process is shown in Fig 2.

### Ferret Viral Challenge Characterisation Study Design

Before infecting human volunteers with a challenge virus, we utilised the Ferret Viral Challenge Model[24] to determine the safety of the virus and help design the later human study (as we have with all of the viruses we use in the HVC Model and have previously described[24–27]). The ferret study was conducted by the Southern Research Institute, Birmingham, Alabama, USA and the study was conducted with the approval of the Institutional Animal Care and Use Committee at Southern Research. The procedures used in this study were designed to conform to accepted practices and to minimise or avoid causing pain, distress or discomfort in the animals. This study consisted of four infectious titres of the challenge virus, one titre group using a comparator (Influenza A/Wisconsin/67/2005 (H3N2) virus which has an established safety profile in the HVC Model, this allowed us to compare the characteristics of this virus with other H3N2 viruses we have used. An additional control group was administered the GMP diluent only. The ferrets were Aleutian’s disease free and seronegative for the challenge strains in question as determined by HAI assay.

The four groups to receive Influenza A/Perth/16/2009 received the following inoculum titres of 1.4 × 10^3^, 1.25 × 10^4^, 1.8 × 10^5^, or 2.35 × 10^6^ TCID_50_/animal. Each animal was inoculated intranasally and the animal’s health was monitored throughout the study. The inoculum doses were determined based on our previous experience in the human and animal viral challenge models [26–29]. Our GMP Influenza A/Wisconsin/67/2005 challenge stock, (which has been used in excess of 1000 human volunteers and has an established safety profile) was used as a comparator control for this study, to gauge the relative pathogenicity.

### Human Viral Challenge Characterisation Study Design

A randomised double-blind study was conducted to determine the optimal infectious titre of the virus and its safety for future use, as we have previously done for multiple viruses[25, 26, 28–33]. A suitable infectious titre would be one that yields infection in a high proportion of volunteers along with mild to moderate clinical symptoms in the majority of those infected with the lowest possible inoculum titre. The study design is summarised in Fig 1. The study was retrospectively registered on clinicaltrials.gov as it is not a requirement in the UK to register such studies, where required future studies will be registered. The authors confirm that all ongoing and related trials for a drug/intervention are registered.

Healthy adult volunteers (subjects) who were serosuitable (HAI < 10) for the challenge virus underwent eligibility assessments which included physical examination, medical history, vital signs, electrocardiogram (ECG), spirometry, clinical chemistry, haematology, and coagulation. The quarantine and challenge phase was conducted at our facility in London. Subjects were screened for serosuitablilty from 56 days to 3 days before inoculation day. Subjects were admitted to the quarantine unit 48 hours before inoculation and absence of a concurrent respiratory illness was confirmed by clinical observation and a negative Direct Fluorescence Antibody Assay (DFA), (Light Diagnostics™ SimulFluor® Respiratory Screen, Merckmillipore).

Volunteers were randomised to one of four infectious titre groups, (2.8 x 10^3^, 2.5 x 10^4^ 3.6 x 10^5^, or 4.7 x 10^6^ TCID_50_) with 6 volunteers in each dose group. Both subjects and investigators were blinded. The subjects were inoculated with the challenge virus on Day 0 and remained isolated in individual en-suite rooms to prevent transmission of the virus to others. Volunteers attended a follow up visit on Day 28 (+/- 5 days) post challenge, at which all baseline and safety assessments were repeated.

Virological, clinical, and safety laboratory assessments were performed during the study. Virus shedding was investigated in nasopharyngeal swabs by TCID_50_ on MDCK cells and by reverse transcriptase quantitative polymerase chain reaction (RT-qPCR/real-time qPCR) which measures viral transcripts, but not live virus[34, 35]. Serology was performed at the Day 28 follow-up visit to determine the presence of Influenza antibodies.

Volunteers completed a standardised symptom diary card that our group have used across multiple clinical studies with three different respiratory viruses. The card has been successfully used to demonstrate previous proof of concept for antiviral, monoclonal antibodies and vaccines. The symptom diary card was completed three times daily to evaluate 10 symptoms of upper respiratory tract (URT), lower respiratory tract (LRT) and systemic respiratory tract (SRT) illness (runny nose, stuffy nose, sneezing, sore throat, earache, malaise, cough, shortness of breath, headache, muscle and/or joint ache) (S1 Table). Each symptom was rated from 0–3; 0 for ‘no symptoms, 1 (mild)–‘just noticeable’, 2 (moderate)–‘clearly bothersome but not affecting daily activities’, and 3 (severe)–‘quite bothersome with an effect on daily activities.

Mucus weights were assessed by subtracting the weight of used tissues from the standardised weight of an unused tissue. Daily directed physical examinations were performed by a physician to assess URT and LRT symptoms. Vital signs, temperature, spirometry and ECG analysis were recorded throughout the quarantine period.

### Virus Infection: Infectivity and Viral Shedding

Laboratory-confirmed Influenza infection was defined by the presence of viral shedding, i.e., at least one positive cell culture assay from a nasopharyngeal swab and/or at least two positive detections qPCR within a 24 hour period post-Influenza inoculation to day of discharge from quarantine. The requirement for two positive results was to avoid any risk of a false positive.

## Results

### Virus Strain Selection

We identified the strain Influenza A/Perth/16/2009 (H3N2) as a lead candidate for challenge virus production. This virus was a vaccine component for influenza seasons 2010–2012. Serological analysis indicated that this virus had the highest serosuitable rate (S1 Fig).

### Ferret Study

**Fig 3:**
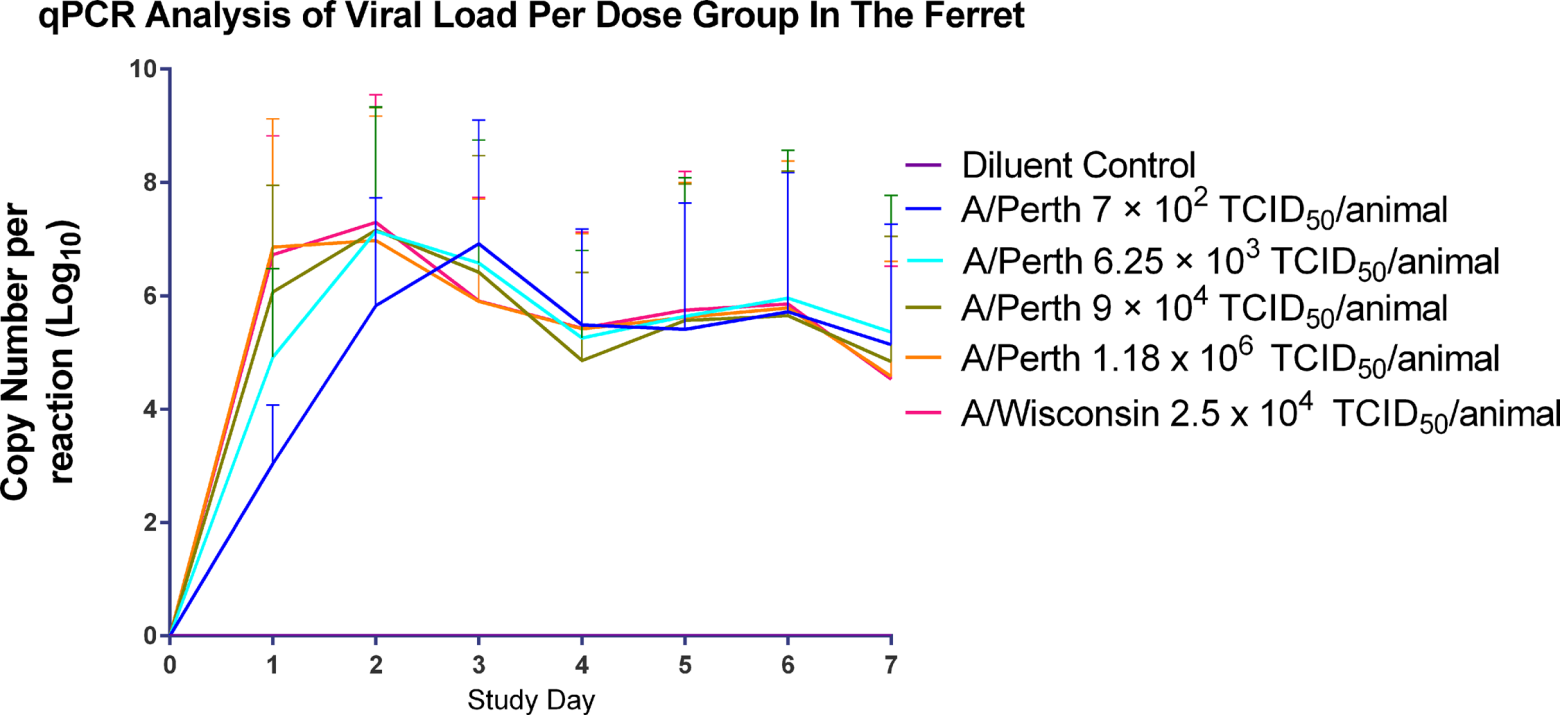
Virus shedding (qPCR) by inoculum titre group in the Ferret Viral Challenge Model.

**Fig 4:**
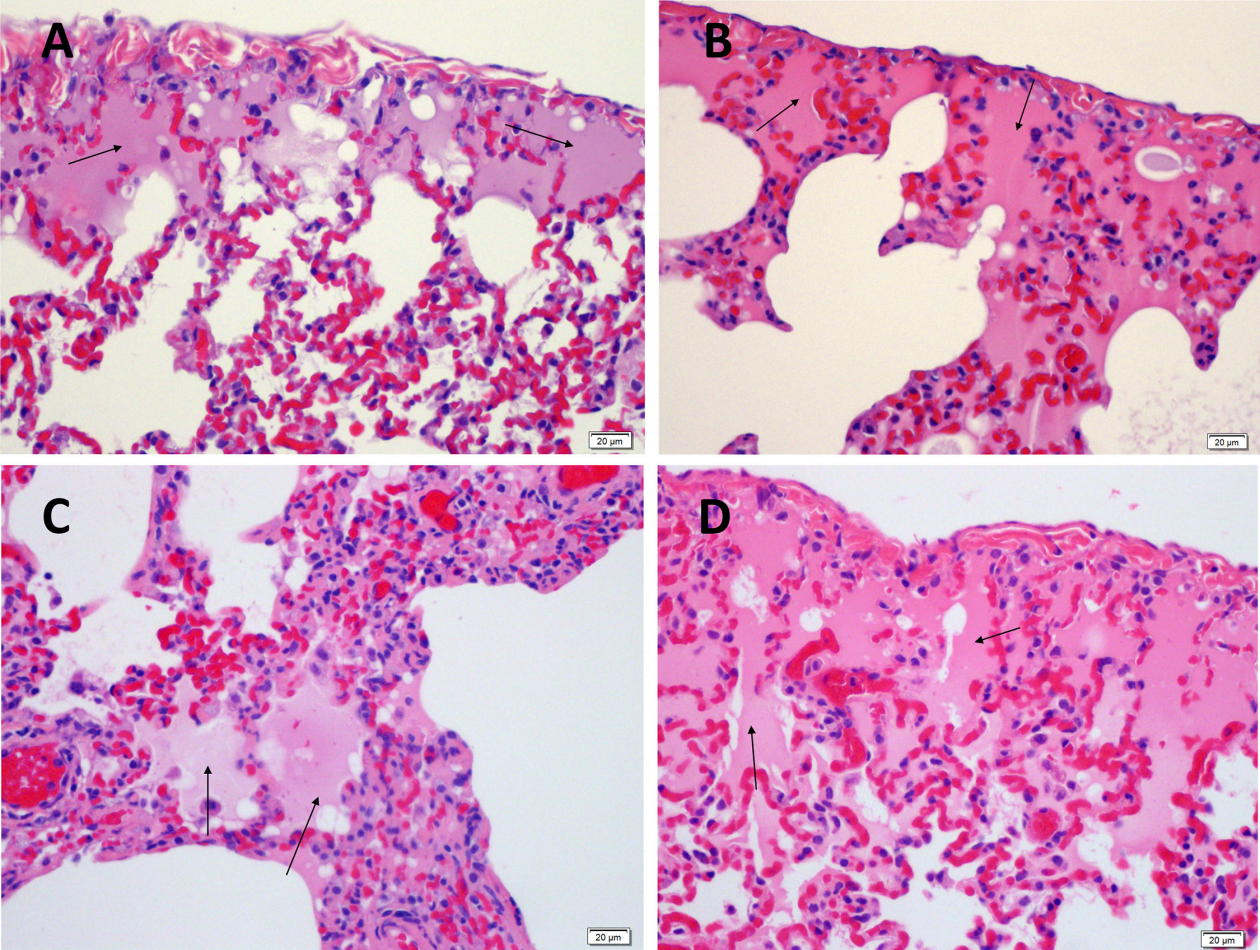
Histopathology of the Ferret Lung Post Challenge with A/Perth/16/2009 or A/Wisconsin/67/2005. A) Ferret Lung Day 4 post inoculation with A/Wisconsin/67/2005 2.5. × 10^4^ TCID_50_/animal. B) Ferret Lung Day 7 post inoculation with A/Wisconsin/67/2005 2.5 × 10^4^ TCID_50_/animal. C) Ferret Lung Day 4 post inoculation with A/Perth/16/2009 1.8 × 10^5^ TCID_50_/animal. D) Ferret Lung Day 7 post inoculation A/Perth/16/2009 1.8 × 10^5^ TCID_50_/animal. Alveolar lumina were filled with hyaline eosinophilic proteinaceous material (edema, see arrows).

As expected for a wild type seasonal Influenza virus strain, all titre groups inoculated with Influenza A/Perth/16/2009 challenge virus in the Good Laboratory Practice (GLP) ferret challenge model showed signs of mild to moderate influenza infection[24, 36–41] with substantial levels of virus shedding detected by qPCR (Fig 3). It was noted that the lowest inoculum titre group (1.4 x 10^3^ TCID_50_/animal) had a delay of one day in peak virus shedding compared to the other titre groups. All animals survived to the end of the study with no adverse illness detected. The viral shedding profile of Influenza A/Perth/16/2009 was comparable to that seen with Influenza A/Wisconsin/67/2005 (Fig 3). The lungs of animals challenged with Influenza A/Wisconsin/67/2005 and Influenza A/Perth/16/2009 were analysed at 4 and 7 days post inoculation. Both challenge groups showed pathology consistent with signs of mild to moderate influenza infection (Fig 4).

As the level of influenza disease caused by the virus was moderate and not severe in accordance with expectations it was considered safe for use in human studies. Had the results of the ferret study indicated that the virus had very low pathogenicity or conversely had caused severe illness, this would have been deemed atypical, the virus would then not have been deemed suitable.

### Clinical Study

#### Volunteers

Twenty-four subjects were enrolled, randomised and completed their follow up at Day 28 (+/-3 days). There were no notable differences in the baseline demographics between the four virus titre groups.

#### Virology and Infectivity

Thirteen of the 24 randomised subjects developed laboratory-confirmed infection across the four viral titre groups. No subjects in the 2.8 x 10^3^ TCID_50_ group had laboratory-confirmed infection. Four subjects (77%) in the 2.5 x 10^4^ titre group, four (77%) in the 3.6 x 10^5^ TCID_50_ titre group and five (83%) in the 4.7 x 10^6^ TCID_50_ titre group had laboratory-confirmed influenza infection (Table 1). Due to the exploratory nature of this study small n numbers used, no statistical comparisons were made.

**Table 1:** Laboratory-confirmed Infection Rates by Definition.

Inoculum (TCID_50_)	Culture Virus Positive (%)	qPCR Virus Positive (%)	Seroconversion (%)	Lab Confirmed Infection (%)
2.8 x 10^3^	0	17	17	0 (0/6)
2.5 x 10^4^	68	68	50	67 (4/6)
3.6 x 10^5^	51	100	33	67 (4/6)
4.7 x 10^6^	68	100	77	83 (5/6)

Definitions:
Culture virus positive; a single positive result. qPCR virus; positive single positive results Seroconversion; a fourfold increase from baseline. Lab confirmed infection; a single culture positive or two qPCR positive results within 24 hours.

**Fig 5:**
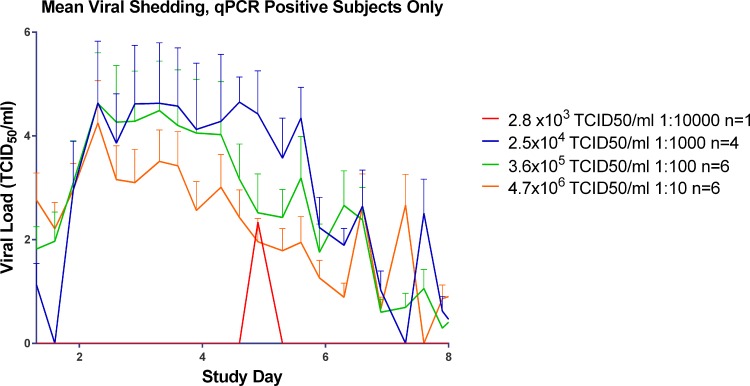
Mean Viral Shedding in qPCR positive subjects in the Human Viral Challenge Model.

**Fig 6:**
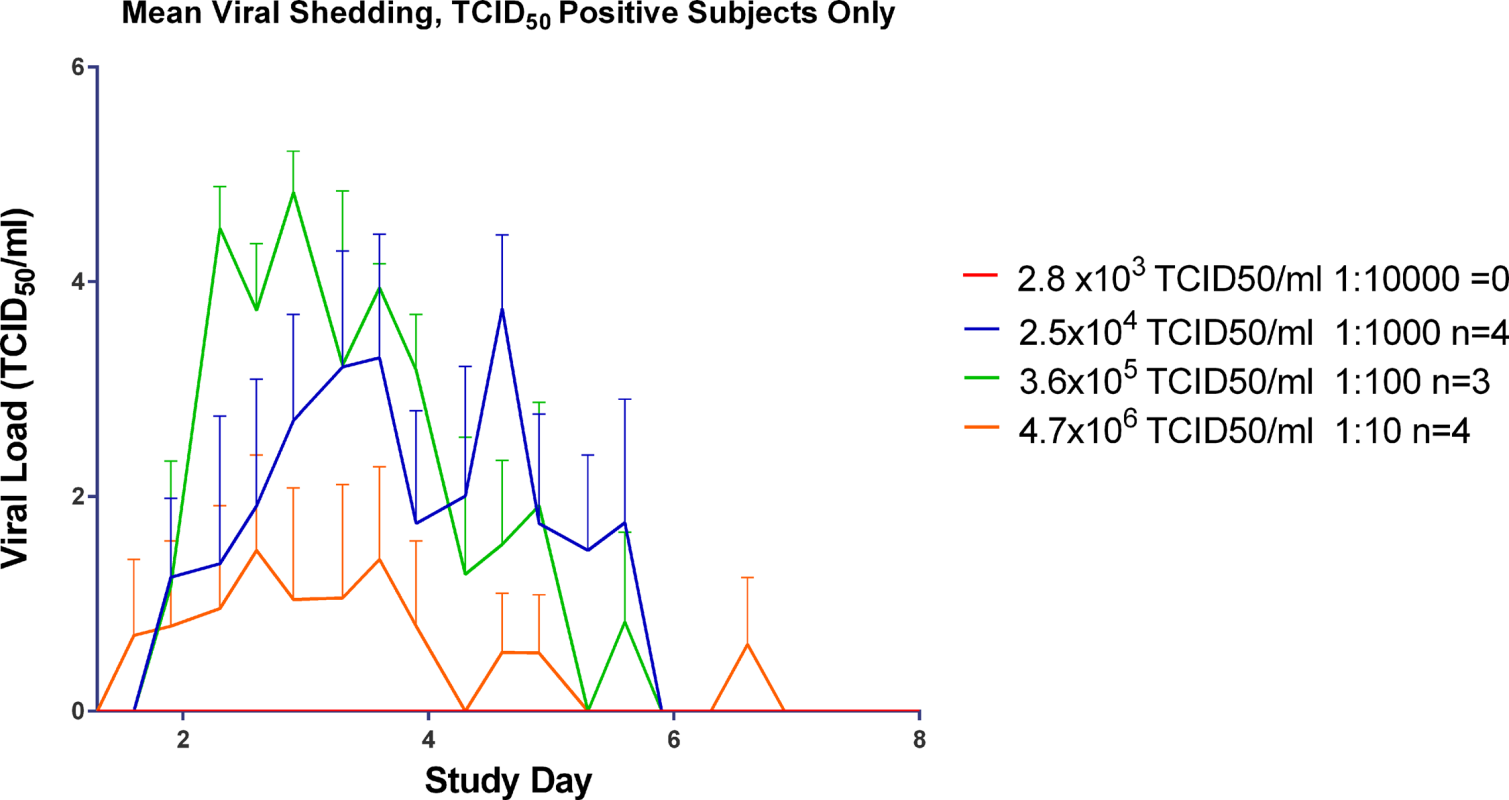
Mean viral shedding by day in TCID_50_ positive subjects in the Human Viral Challenge model.

In the 2.8 x 10^3^ TCID_50_ group one subject had a positive result for viral shedding as detected at one time point by qPCR (Fig 5), but this was not enough for laboratory-confirmed infection; the subject was also negative for viral shedding by tissue culture (Fig 6). This subject did not seroconvert. None of the six subjects had laboratory-confirmed infection post viral challenge.

In the 2.5 x 10^4^ TCID_50_ group all four were positive by tissue culture and by qPCR (Fig 5). One of the laboratory-confirmed infected subjects did not seroconvert. In the 3.6 x 10^5^ TCID_50_ group, four subjects had laboratory-confirmed infection. Three were positive by both tissue culture and qPCR (Table 1). In the 4.7 x 10^6^ TCID_50_ group, five subjects had laboratory-confirmed infection (Table 1). Four of the 4.7 x 10^6^ TCID_50_ group were positive by tissue culture and five had at least two positive detections by qPCR of nasopharyngeal swab.

**Fig 7:**
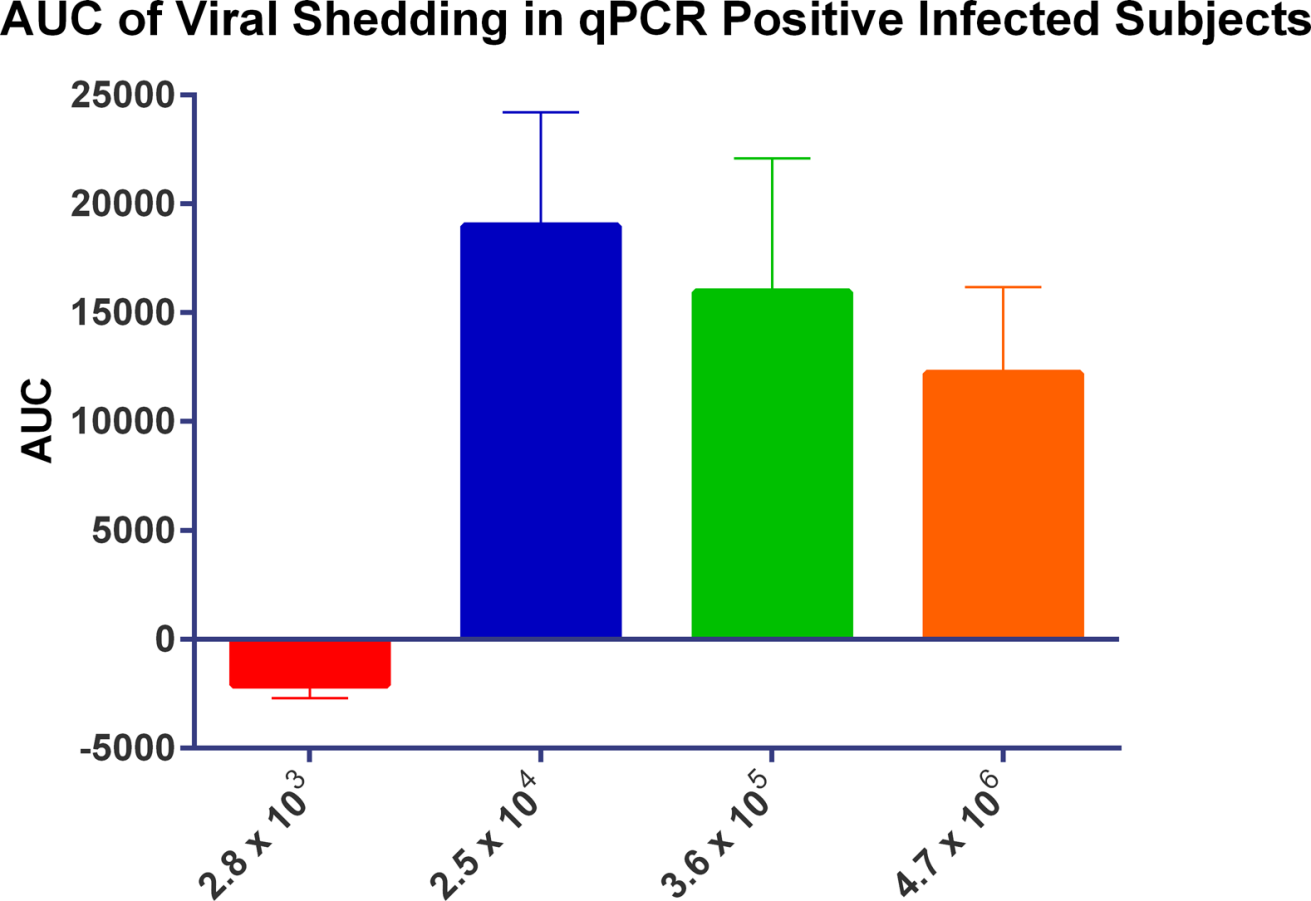
The impact of inoculum titre on viral load in infected subjects.

The time to peak of viral load as detected by the TCID_50_ assay (Fig 6) appeared to be related with the inoculum titre given with the 3.6 x 10^5^ TCID_50_ titre group peaking at Day 3 post inoculation followed by the 2.5 x 10^4^ TCID_50_ titre group peaking at Day 5. While the viral shedding in the 4.7 x 10^6^ TCID_50_ titre appeared to peak at Day 3 post inoculation, the levels of virus shedding were considerably lower than those for 3.6 x 10^5^ and 4.7 x 10^6^ inoculum titre groups and had substantially lower AUCs (Fig 7). All virus titre groups were negative for virus shedding as determined by TCID_50_ by Day 7 post-inoculation.

By comparison, for all groups with laboratory-confirmed infection (2.5 x 10^4^ TCID_50_, 3.6 x 10^5^ TCID_50_ and 4.7 x 10^6^ TCID_50_) the peak in viral shedding as detected by qPCR occurred at Day 2 post inoculation (Fig 5.) Interestingly, the lowest of these inoculum titre groups (2.5 x 10^4^ TCID_50_) maintained peak virus shedding for approximately four days post inoculation (Days 2–6), whilst the higher titre group using 3.6 x 10^5^ TCID_50_ maintained peak shedding for two days (Days 2–4) as determined by qPCR, and the 4.7 x 10^6^ TCID_50_ titre group began a decline from peak shedding after Day 2 post inoculation, qPCR.

### Seroconversion

Seroconversion was determined between Day -2 and Day 28 (Table 1). In the 2.8 x 10^3^ TCID_50_ group only one subject seroconverted, in the 2.5 x 10^4^ TCID_50_ group three subjects seroconverted, while only two subjects seroconverted in the 3.6 x 10^5^ TCID_50_ group. All subjects seroconverted (6 of 6) in the 4.7 x 10^6^ TCID_50_ inoculum titre group.

### Illness Measures: Symptom Scores and Mucus Weights

#### Symptom Scores

**Fig 8:**
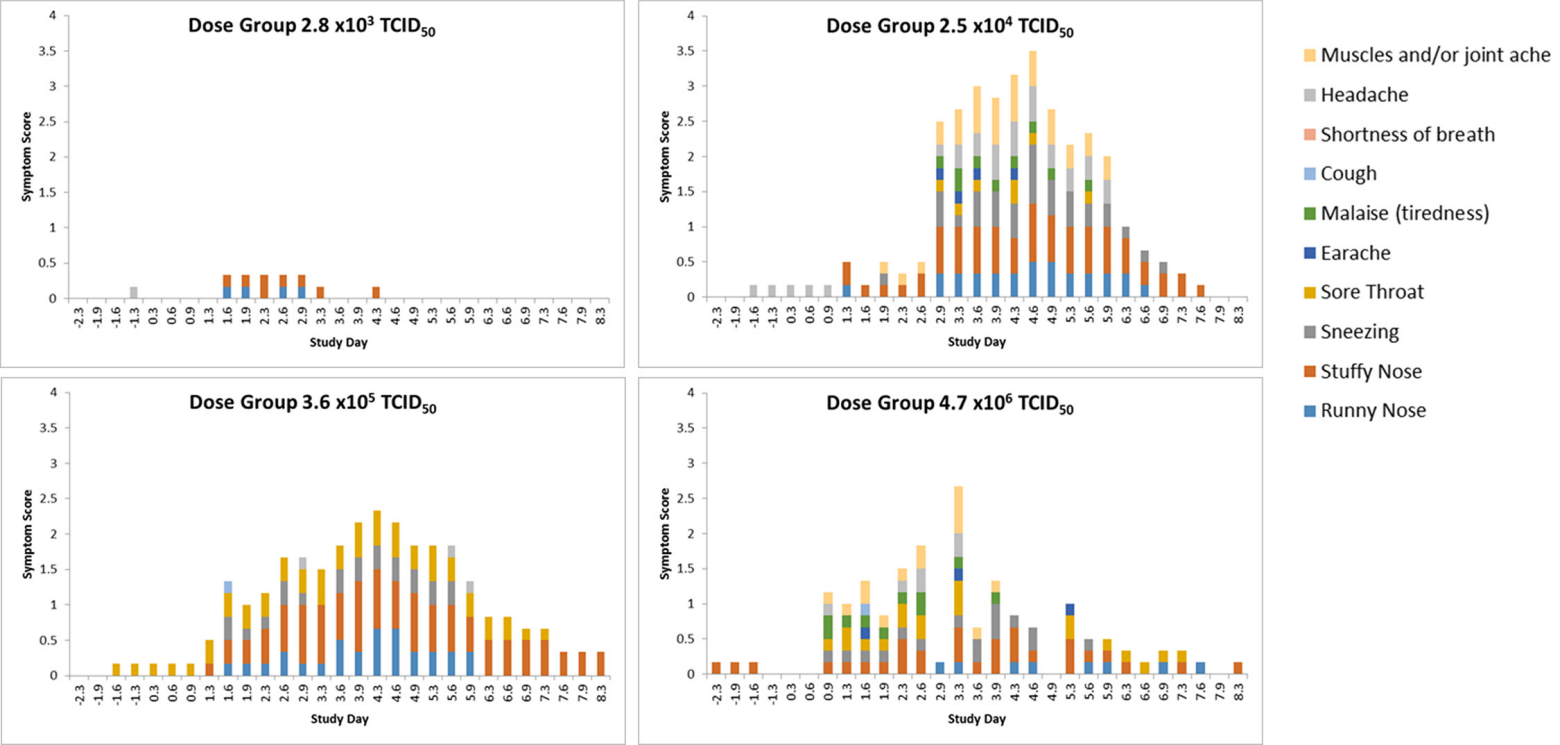
Progression of symptoms over the course of infection in all subjects, by titre group (infected and non-infected volunteers).

Volunteers self-reported their symptoms three times per day during quarantine (Day -2/-1 through to Day 8), using our established symptom diary card. The majority of the subjects in the three highest inoculum titre groups had symptoms consistent with an URT virus infection as shown in Fig 8.

**Fig 9:**
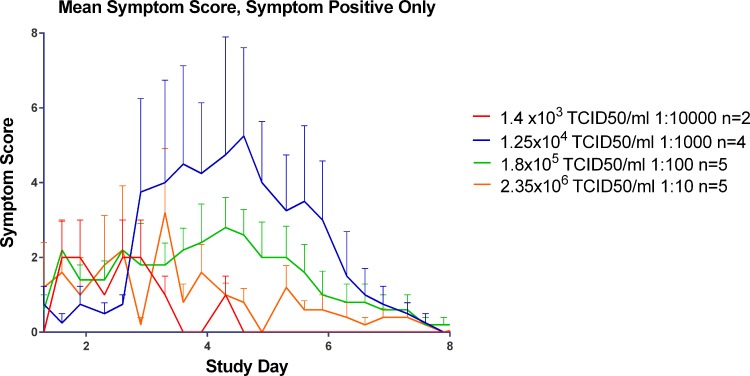
Mean symptom score by day in symptom positive subjects in the Human Challenge Viral Model.

For the three titre groups with laboratory-confirmed infection (2.5 x 10^4^, 3.6 x 10^5^ and 4.7 x 10^6^ TCID_50_) the time of peak symptoms appeared to be directly proportional to inoculum titre (Fig 9) with higher inoculum titres leading to the onset of symptoms sooner than lower titres. The group inoculated with 2.5 x 10^4^ TCID_50_/ml of virus displayed peak symptoms at the end of Day 4 post inoculation while the group inoculated with 3.6 x 10^5^ TCID_50_ of virus had peak symptomology at the beginning of Day 4 post infection and the group inoculated with 4.7 x 10^6^ TCID_50_/ml of virus had peak symptomology on the beginning of Day 3 post inoculation.

**Fig 10:**
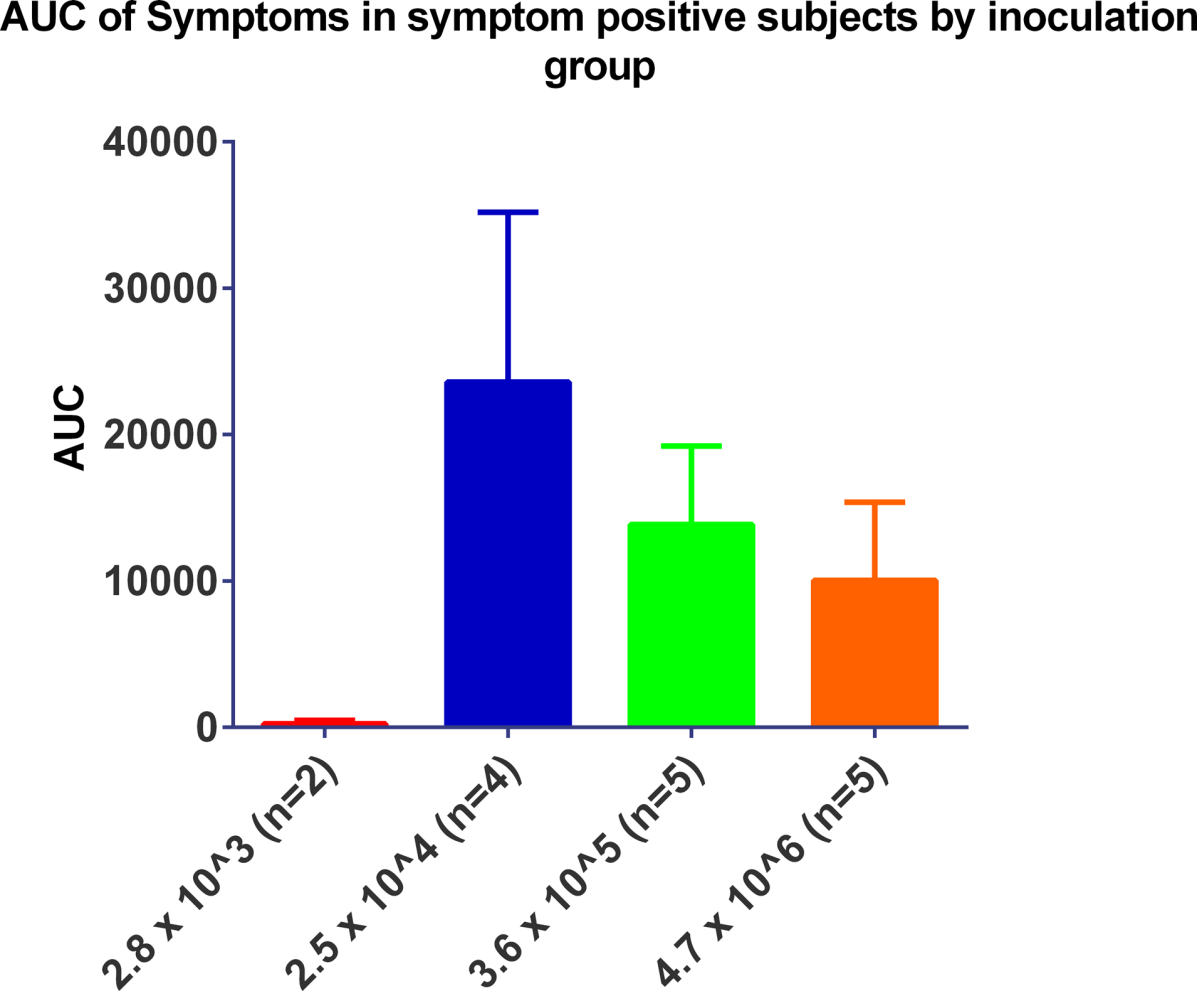
The impact of inoculum titre on symptomology in subjects positive for symptoms.

Despite the peak in symptomology being directly proportional to inoculum titre, the severity of symptoms was inversely proportional to inoculum titre as shown in Fig 10. With the exception of any symptoms that were recorded prior to Day 0, post inoculation the predominating symptoms were stuffy nose followed quickly by a runny nose and sneezing. In the higher titre groups (3.6 x 10^5^ and 4.7 x 10^6^ TCID_50_) sore throat became apparent by the end of Day 1 post inoculation. While malaise and muscle ache were reported it did not seem to be titre specific and generally only occurred around the peak of symptoms.

#### Mucus weights

**Fig 11:**
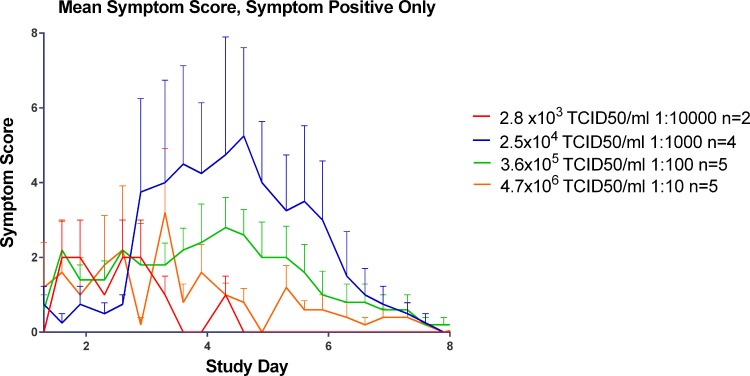
Mean mucus weight by day in all subjects in the Human Viral Challenge Model.

In the groups with laboratory-confirmed infection (2.5 x 10^4^, 3.6 x 10^5^ and 4.7 x 10^6^ TCID_50_/ml) there was an observed apparent relationship between mucus weight and total symptoms (Figs 10 and 11).

The 2.5 x 10^4^ TCID_50_ titre group mucus production peaked at Day 2 with similar levels also being recorded on Day 3 and with the majority of mucus being produced from Day 1–5 post infection (Fig 11). Similarly, the 3.6 x 10^5^ TCID_50_ titre group peaked in mucus production at Day 3 with also the majority of mucus production being between Days 1–5 post infection. The peak mucus production weight for the 3.6 x 10^5^ TCID_50,_ was, however, considerably lower than the levels recorded in the 2.5 x 10^4^ titre group with 2.7 g peak mucus weight recorded in the 3.6 x 10^5^ TCID_50_ group.

In contrast to the 3.6 x 10^5^ TCID_50_ and 2.5 x 10^4^ TCID_50_ titre groups, very low levels of mucus were produced in both the highest and lowest titre groups such that a meaningful peak mucus production day could not be determined in the 2.8 x 10^3^ TCID_50_ and 4.7 x 10^6^ TCID_50_ test titre groups.

#### Virus Expected Events and Adverse Events

A Virus Expected Event (VEE) is that which would be recorded in a symptom diary card or is consistent with Influenza like illness (ILI) and were not considered to be adverse events (AEs). There were 13 VEEs.

Six AEs were procedure related, but were not of serious concern, the volunteers recovered quickly. Only one subject described an AE possibly related to virus, this was from the 4.7 x 10^6^ TCID_50_ group; the subject demonstrated a fall in the FEV_1_ by 22.09% from baseline and a fall in the FVC by 27.43% from baseline on Day 4 post-inoculation but showed complete resolution. There were no SAEs.

## Discussion

We manufactured a new GMP wild-type Influenza virus, suitable for use in the HVC Model for the evaluation of antiviral agents, immunomodulators, monoclonal antibodies, vaccines and other novel treatments. The primary objective of the study was to determine a suitable safe infectious titre of Wild-type Influenza A/Perth/16/2009 (H3N2) virus for use in future studies.

The virus was manufactured under GMP conditions and an extensive panel of adventitious agent testing was performed. The GLP ferret study showed that wild-type influenza A/Perth/16/2009 was mild to moderate in pathogenicity and good viral shedding was detected (Figs 3 and 4). This gave us confidence to move into the HVC model.

Of the four inoculum titre groups in this study (2.8 x 10^3^, 2.5 x 10^4^, 3.6 x 10^5^ and 4.7 x 10^6^ TCID_50_) only three had laboratory-confirmed infection as defined by either a TCID_50_ positive nasal wash sample or two qPCR positive samples within a 24 hour period. The laboratory-confirmed infection rate ranged from 77–83% (Table 1).

The higher the virus inoculum titre given, the sooner peak infectious viral shedding occurred as detected by TCID_50_ assay (Fig 6). However for virus detected by qPCR all the laboratory-confirmed infected groups had peak shedding by Day 2 post inoculation regardless of inoculum titre (Fig 5).

While a high initial viral inoculum titre can lead to an early peak in viral shedding, it does not seem to result in a prolonged production of viral transcripts as is seen with lower inoculum titres. It may be that the higher infectious titres result in an early burst of viral replication due to the number of infectious particles present. However, the higher titre groups have a higher multiplicity of infection thus generating defective interfering particles resulting in the Von Magnus effect[42] leading to aborted replication and ultimately a shorter duration of virus replication.

**Fig 12:**
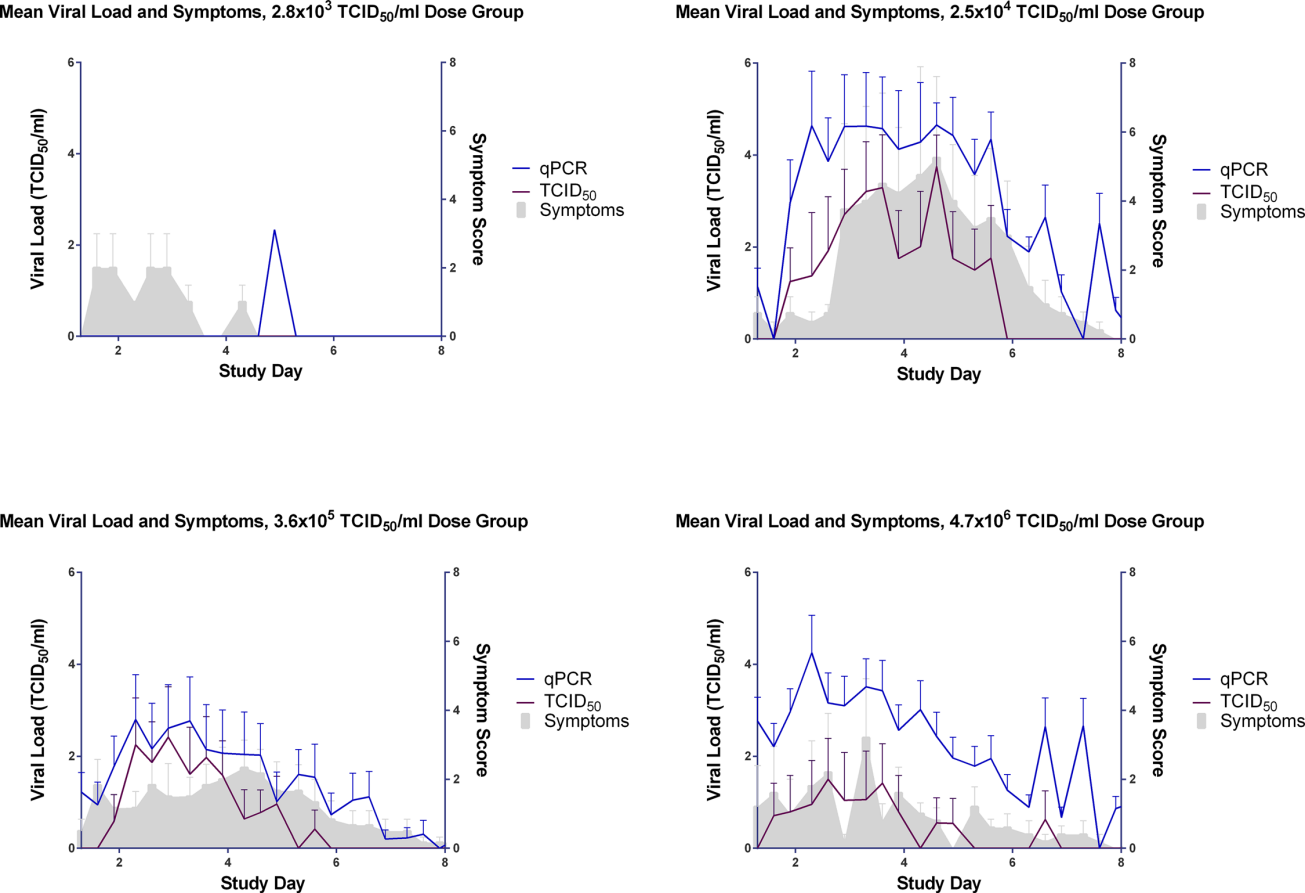
The effects of virus inoculum titre on virus shedding and symptoms in the chosen inoculum titre group in all subjects (infected and non-infected volunteers).

Stuffy, runny noses and sneezing were the predominating symptoms in the majority of cases. Muscle ache and sore throats only really became apparent in the titre groups with the more severe infections (Fig 8). Viral shedding was typically seen to peak prior to the peak onset of symptoms, as can be seen in Fig 12 in the groups including all challenged subjects including non-infected volunteers. This is of particular significance as it can enable the dissemination of virus prior to the host falling ill and so enhance the spread of virus throughout a population (as reported previously [30, 43]).

In this study there was substantial variation in the severity of symptoms reported by subjects as can be seen in Fig 8 based on initial inoculum titre; this variation in symptoms is expected as it reflects the diversity of the host population.

The association between mucus weights may be a suitable objective measure to determine the severity of influenza infection as opposed to relying solely on the subjective reporting of subjects (Figs 8–10 and 12).

This new challenge virus is safe and suitable for use in the HVC Model, and is able to consistently induce mild to moderate influenza-like illness characteristic of influenza infection. We drew upon our past experience of studies with more than 1000 subjects safely inoculated using our HVC model over the course of 15 years to enable us to determine the suitability of this strain for future studies[25–27, 30–33].

The 3.6 x 10^5^ TCID_50_ titre was chosen as the most suitable titre to be used for future HVC studies, as viral load peaked earlier as detected by TCID_50_ as shown in Fig 4. By qPCR the virus shedding reached its peak at the same time as the 2.5 x 10^4^ TCID_50_ titre group (Fig 6) although this lower titre group had a more prolonged period of peak shedding than the titre group chosen and higher AUC (Fig 5). Overall, the 2.5 x 10^4^ TCID_50_ titre group exhibited higher recorded symptoms and mucus production than the chosen titre of 3.6 x 10^5^ TCID_50_ (Figs 10–12).

Both the 2.5 x 10^4^ and 3.6 x 10^5^ titre groups gave a good range of reported symptoms, however the reported symptoms of the 3.6 x 10^5^ titre group appeared to be more consistent (Fig 8). Consequently the key driver for the chosen inoculum titre was consistency in response to infection, so that these studies could be conducted at different facilities and with different inoculum batches. While the 2.5 x 10^4^ virus titre group resulted in the highest AUCs for viral shedding and reported symptoms, a slight variation in the virus inoculum titre could potentially result in low reported symptom scores and viral shedding (Figs 7 and 10), while the 3.6 x 10^5^ virus titre group could still undergo some variation in viral titre and inoculation would still be able to induce consistent Influenza-like illness. This study was conducted in 2013 and to date 153 volunteers have been inoculated with a virus titre 3.6 x 10^5^ TCID_50_ with an overall infection rate of 69%.

Very importantly, in this study the optimal infectious titre of 3.6 x 10^5^ TCID_50_ was substantially lower than that used in recently reported influenza challenge studies with Influenza A/California/4/2009-like viruses[44, 45]. We believe this will be a more sensitive model for evaluation of treatment as the initial input titre of virus will be low and thus mimic a natural infection more closely. The difference in pathology of H1N1 and H3N2 strains may explain why higher inoculum titres were required in the recent H1N1 studies than in this H3N2 study[44].

Another explanation for the difference in the required inoculum titre may be the impact the pre-challenge HAI titre had on infection rate. In our model and in the majority of historical papers, serosuitability was defined as <10 HAI units, whilst in a recent paper in which a serosuitability rate of <40 HAI units was used, an infectious titre of 10^7^ TCID_50_ was required, two logs higher than in our study[44]. A similar paper with an H1N1 virus used <10 HAI units but still required a much higher infectious titre A/Perth/16/2009 used in this study [45].

Previously the HVC Model has been limited to those between 18 and 45 years, we have now extended this age range to 55 years (publication in preparation). The results of this study are consistent with our previous studies in more than 1000 subjects using other H3N2 viruses, namely Influenza A/Panama/2007/1999(H3N2) and Influenza A/Wisconsin/67/2005 (H3N2)[26–29]. The data from these studies can be extrapolated for use in the design of future studies with our new virus.

## Conclusions

Here we report the results from a HVC study using a new wild-type GMP H3N2 virus, and were able to demonstrate a higher infection rate than previously published influenza challenge studies, but using a lower inoculum titre[46]. The signs and symptoms observed in this study were consistent with a natural influenza infection.

By using a strict definition of serosuitable (<10 HAI) subjects, in line with historical studies, we are able to ensure a consistent approach to the HVC Model improving reproducibility by limiting variance in clinical outcome due to varying pre-inoculation antibody titres.

Our wild-type influenza A/Perth/16/2009 (H3N2) stock has been produced in sufficient quantity to enable the same batch to be used throughout the development of the different planned models, thus building up an important body of safety and pathogenicity data.

The use of a wild-type H3N2 virus in the HVC Model may be more appropriate than an H1N1 challenge virus, given that H3N2 viruses cause the greatest levels of morbidity and mortality. This H3N2 virus gave a good infection rate, good influenza-like symptoms and virus shedding.

## Supporting Information

S1 Fig Distribution of specific Antibody in a panel of serum from 245 subjects tested against 4 H3N2 influenza strains. (NDA, No detectable Antibody).
(TIF)

S1 Table Definitions of illness and infection. (DOCX)

S1 Appendix Clinical Study Protocol. (PDF)

S2 Appendix Consort Checklist. (DOC)
