# Correction: Accelerating Influenza Research: Vaccines, Antivirals, Immunomodulators and Monoclonal Antibodies. The Manufacture of a New Wild-Type H3N2 Virus for the Human Viral Challenge Model

**DOI:** 10.1371/journal.pone.0157211

**Published:** 2016-06-09

**Authors:** Daniel J. Fullen, Nicolas Noulin, Andrew Catchpole, Hosnieh Fathi, Edward J. Murray, Alex Mann, Kingsley Eze, Ganesh Balaratnam, Daryl W. Borley, Anthony Gilbert, Rob Lambkin-Williams

Fig 11, “Mean mucus weight by day in all subjects in the Human Viral Challenge Model.” is a duplicate of Fig 9, “Mean symptom score by day in symptom positive subjects in the Human Challenge Viral Model.” Please view the correct Fig 11 here.

**Fig 11:**
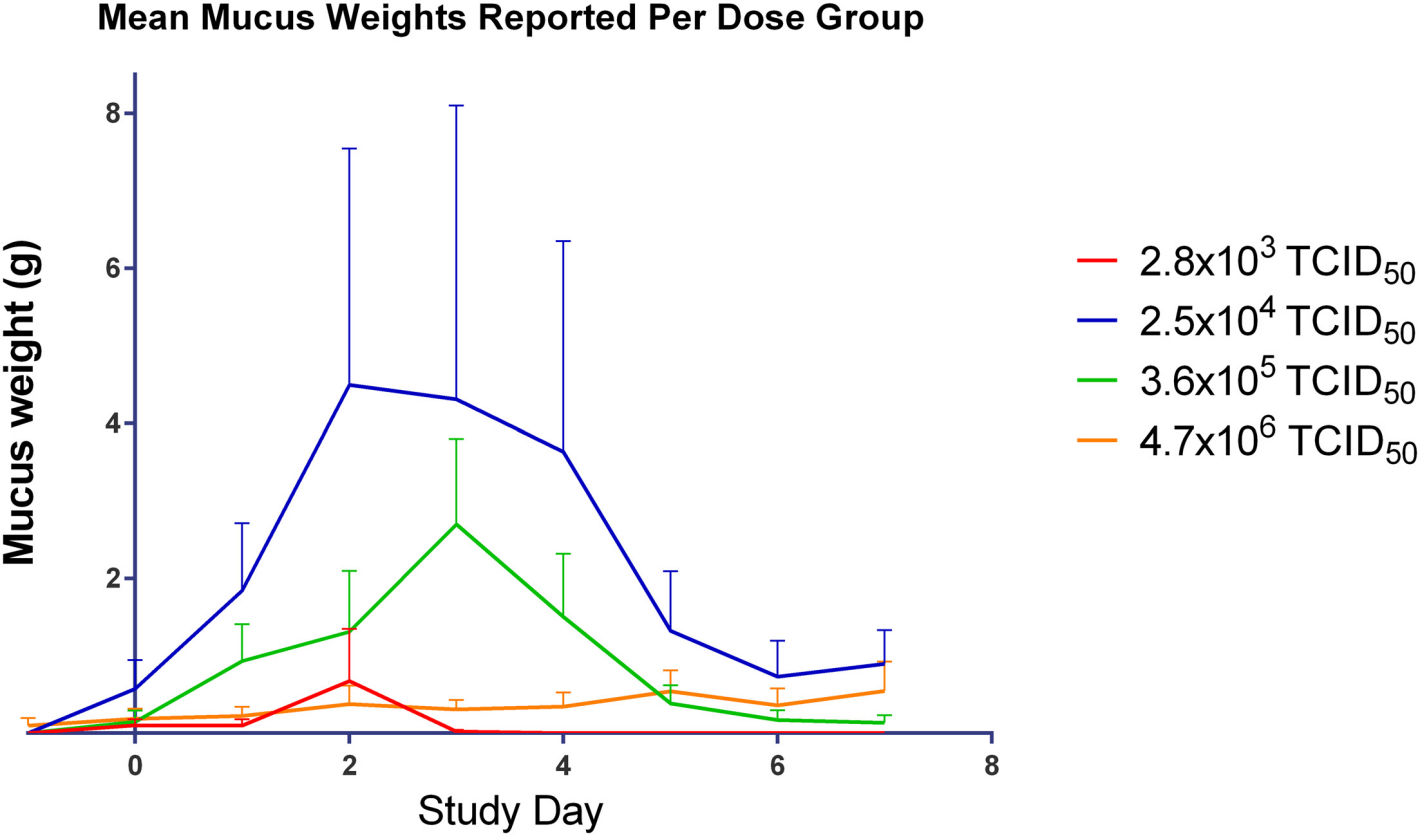
Mean mucus weight by day in all subjects in the Human Viral Challenge Model.
