# Supplementary figures and images for: Accelerating Influenza Research: Vaccines, Antivirals, Immunomodulators and Monoclonal Antibodies. The Manufacture of a New Wild-Type H3N2 Virus for the Human Viral Challenge Model
Source: PLoS One. 2016 Jan 13;11(1):e0145902. doi: 10.1371/journal.pone.0145902 (PMC4711822; doi:10.1371/journal.pone.0145902)

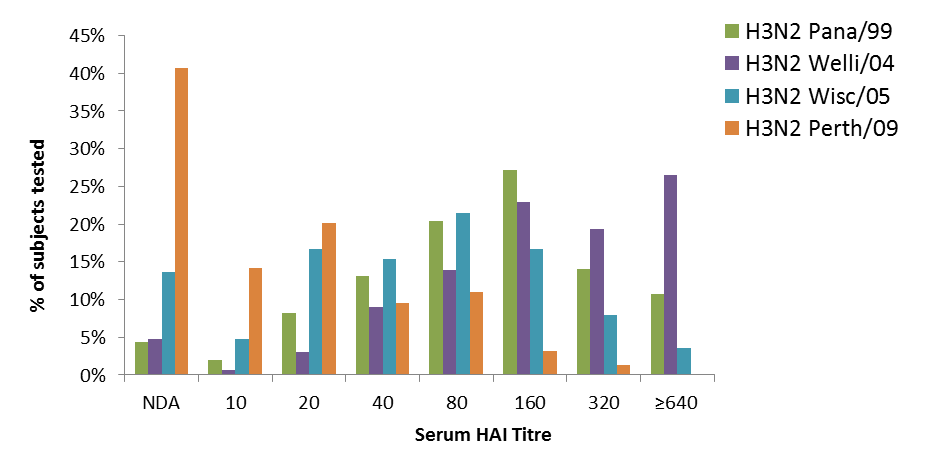

Supplement: S1 Fig — (NDA, No detectable Antibody). (TIF) [file pone.0145902.s001.tif]
