# Supplementary material for: Accelerating Influenza Research: Vaccines, Antivirals, Immunomodulators and Monoclonal Antibodies. The Manufacture of a New Wild-Type H3N2 Virus for the Human Viral Challenge Model
Source: PLoS One. 2016 Jan 13;11(1):e0145902. doi: 10.1371/journal.pone.0145902 (PMC4711822; doi:10.1371/journal.pone.0145902)
Supplement: S1 Table — (DOCX) [file pone.0145902.s002.docx]

| INFECTION/  ILLNESS TERM | CRITERIA FOR INFLUENZA STUDIES |
| --- | --- |
| Viral shedding | - A positive cell culture assay at least once during quarantine post-Viral Challenge; or - If analysed by polymerase chain reaction (PCR), at least 2 positive detections by any PCR assay (from 2 independent samples) within 2 days of each other. |
| Seroconversion | A ≥ 4 fold increase in influenza-specific antibodies from baseline to follow-up post-quarantine. |
| Laboratory-confirmed influenza infection | - Viral shedding   and/or   - Seroconversion. |
| Influenza-like-illness  (ILI) | Any occurrence of the following:   - Upper respiratory tract illness - Lower respiratory tract illness - Systemic illness - Febrile illness. |
| Laboratory-confirmed influenza illness | Any incidence of:   - Influenza-like-illness, AND - Laboratory-confirmed infection. |
| Sub-clinical infection | - Not meeting the definition of influenza-like illness, BUT - Meeting the definition of laboratory-confirmed infection. |
| Upper respiratory tract illness (URTI) | Any one of the following signs and/or symptoms on 2 consecutive scheduled assessments, at least 1 of which must feature Grade 2 severity, or if any of the following attain Grade 3 severity once:  Self-reported symptoms: rhinorrhoea (runny nose), nasal congestion (stuffy nose), sore throat, sneezing  Physician findings: nasal discharge, otitis, pharyngitis, sinus tenderness. |
| Lower respiratory tract illness (LRTI) | Any one of the following signs and/or symptoms on 2 consecutive scheduled assessments, at least 1 of which must feature Grade 2 severity, or if any of the following attain Grade 3 severity once: Self-reported symptoms: cough, shortness of breath  Physician findings: abnormal breath sounds (new wheezing, râles, rhonchi, other). |
| Febrile illness (FI) | - Any occurrence of temperature ≥ 37.9 ^o^C (confirmed by a repeat measurement as ≥ 37.9 ^o^C within 20 to 60 minutes), or - Temperature change (post inoculation) of ≥ 2 standard deviations (SD) greater than a baseline measure (from all scheduled temperatures taken by each method on Day -1). |
| Systemic illness (SI) | - Fulfils the criteria for FI, or fulfils the definition of URTI and/or LRTI   and   - Any one of the following symptoms on 2 consecutive scheduled assessments, at least one of which must feature Grade 2 severity, or if any of the following attain Grade 3 severity once:   - malaise   - headache,   - muscles and/or joint ache. |
| Non-sick and uninfected | - Does not fulfil ILI, and - Does not fulfil laboratory-confirmed evidence of infection. |
