# Supplementary material for: Accelerating Influenza Research: Vaccines, Antivirals, Immunomodulators and Monoclonal Antibodies. The Manufacture of a New Wild-Type H3N2 Virus for the Human Viral Challenge Model
Source: PLoS One. 2016 Jan 13;11(1):e0145902. doi: 10.1371/journal.pone.0145902 (PMC4711822; doi:10.1371/journal.pone.0145902)
Supplement: S1 Appendix — (PDF) [file pone.0145902.s003.pdf]

CLINICAL STUDY PROTOCOL
RVL-vCS-003
A Randomised, Double Blind Study
To Characterise Influenza A/Perth/16/2009(H3N2) Virus

Version:	Draft v3.011_03MAR2014
Sponsor:	Retroscreen Virology Limited (RVL)<br>Queen Mary BioEnterprises Innovation Centre<br>42 New Road<br>London<br>E1 2AX<br>United Kingdom (UK)
Chief Investigator:	Dr Hosnieh Fathi<br>Retroscreen Virology Limited<br>Queen Mary BioEnterprises Innovation Centre<br>42 New Road<br>London<br>E1 2AX
Study Sites	Retroscreen Virology Limited<br>Queen Mary BioEnterprises Innovation Centre<br>42 New Road<br>London<br>E1 2AX<br><br>Retroscreen Virology Limited<br>Angel House<br>24 Station Road<br>Ely<br>Cambridgeshire<br>CB7 4BS<br><br>The Clinic & Laboratory Centre<br>Bourn Hall<br>Bourn<br>Cambridge<br>CB23 2TN<br><br>Retroscreen Virology Limited<br>Manchester Science Parks<br>Kilburn House<br>Lloyd Street North<br>Manchester

M15 6SE

REC/Main REC Reference: 13/LO/0911

1. STUDY PERSONNEL CONTACT LIST

NAME	CONTACT DETAILS
Sponsor	Gareth Ridge<br>Retroscreen Virology Limited<br>Queen Mary BioEnterprises Innovation Centre<br>42 New Road<br>London<br>E1 2AX<br>Tel: 020 7756 1355<br>E-mail: g.ridge@retroscreen.com
Chief Investigator (CI)	Dr Hosnieh Fathi<br>Retroscreen Virology Limited<br>Queen Mary BioEnterprises Innovation Centre<br>42 New Road<br>London<br>E1 2AX<br>Tel: 020 7756 1300<br>E-mail: h.fathi@retroscreen.com
Sponsor's Medical Expert 1 (SME 1)	Dr Ganesh Balaratnam<br>Retroscreen Virology Limited<br>Queen Mary BioEnterprises Innovation Centre<br>42 New Road<br>London<br>E1 2AX<br>Tel: 020 7756 1376<br>E-mail: g.balaratnam@retroscreen.com
Sponsor's Medical Expert 2 (SME 2)	Dr Martin Johnson<br>Retroscreen Virology Limited<br>Queen Mary BioEnterprises Innovation Centre<br>42 New Road<br>London<br>E1 2AX<br>Tel: 07583 908775<br>E-mail: m.johnson@retroscreen.com
Other Parties	Please see the Study Master File for details of 3rd Party Contractors, Sub-Investigators, Laboratories, Data Management and Statistics suppliers and all other suppliers.

The CI will conduct this study in accordance with the standards of Good Clinical Practice (GCP)[1], the Declaration of Helsinki (1996)[2] and the study protocol.

2. SIGNATURE PAGE

SPONSOR'S AUTHORISATION

A designated professional representative of RVL will verify adherence to the protocol, and the accurate and complete recording of data in the source documents.

PROTOCOL AGREED BY SPONSOR REPRESENTATIVE

SPONSOR REPRESENTATIVE	
SIGN____________________________	[ ][ ] / [ ][ ][ ] / [ ][ ]
**GARETH RIDGE**	

PROTOCOL AGREED TO BY CHIEF INVESTIGATOR (CI)

I have read and agree to this protocol. I am aware of my responsibilities as a CI under the guidelines of ICH GCP[1], the Declaration of Helsinki (1996)[2] and the study protocol. I agree to conduct the study according to these guidelines and to appropriately direct and assist the staff under my control who will be involved in the study.

Under certain circumstances, suitably qualified and experienced delegates will undertake my responsibilities as CI. Reference to CI in this protocol therefore also includes my designated delegates.

CHIEF INVESTIGATOR		
SIGN____________________________	DATE	[ ][ ] / [ ][ ][ ] / [ ][ ]
**DR HOSNIEH FATHI**		

The completed protocol agreement signifies review and acceptance of the protocol by the CI prior to initiation of the study and must be signed by both parties. Once signed, the original must be kept on file by RVL and the CI must retain a copy.

Table of Contents

1. STUDY PERSONNEL CONTACT LIST 3

2. SIGNATURE PAGE 5

3. PROTOCOL SYNOPSIS 13

4. ABBREVIATIONS 17

5. RVL DEFINITIONS 21

6. INTRODUCTION 23
6.1 Retroscreen Virology 23
6.2 Influenza 23
6.3 The Human Viral Challenge Model 24
6.3.1 Rationale for the Study Design 24
6.3.2 Potential Risks and Benefits for the Volunteers 24
6.3.2.1 Risk of transmission of Challenge Virus 25
6.3.2.2 Safety Measures 26

7. STUDY OBJECTIVES AND ENDPOINTS 27
7.1 Primary Objective 27
7.1.1 Primary Endpoint 27
7.2 Safety Objective 27
7.2.1 Safety Endpoints 27
7.3 Secondary Objectives 27
7.3.1 Secondary Endpoints 27
7.3.2 Comparison of Optimum Challenge Virus Titre between Cohorts 29
7.4 Dichotomous Objectives 29
7.4.1 Dichotomous Endpoints 29
7.5 Exploratory Objectives 30
7.5.1 Exploratory Endpoints 30
7.6 Discovery Objectives 30
7.6.1 Discovery Endpoints 30

8. STUDY DESIGN 31
8.1 Screening Phase 32
8.1.1 Pre-Screening Visit (Cohort 2 only) 32
8.1.2 Study-Specific Screening Visit (All Cohorts) 32
8.2 Quarantine Phase 32
8.3 Follow up Phase 33
8.3.1 Day 28 Visit (± 5 days) 33
8.4 Duration of the Study 34
8.5 End of Study 34
8.6 Stopping Criteria 34

9. STUDY POPULATION 36
9.1 Number of Subjects 36
9.1.1 Cohort 1 36
9.1.2 Cohort 2 36
9.2 Inclusion Criteria 36
9.3 Exclusion Criteria 37
9.4 Subject Numbering 40
9.5 Discontinuation of Subject Participation 40

9.5.1 Subject Withdrawal 41
9.5.2 Reserve Subjects 41

10. STUDY RESTRICTIONS 43
10.1 Concomitant Medications 43
10.2 Contraception 43
10.3 Other Restrictions 43

11. CHALLENGE VIRUS 44
11.1 Production 44
11.2 Supply and Accountability 44
11.3 Storage 44
11.4 Preparation and Administration 44
11.5 Disposal 44
11.6 Unused Challenge Virus 44

12. RANDOMISATION AND BLINDING 46
12.1 Randomisation 46
12.1.1 Cohort 1 46
12.1.2 Cohort 2 46
12.2 Blinding (Cohort 1) 46
12.3 Unblinding 46

13. STUDY PROCEDURES 47
13.1 Time and Events Schedule 47
13.2 Screening Phase 50
13.2.1 Pre-Screening Visit (from Day -80) (Cohort 2 only) 50
13.2.2 Study Specific Screening (Day -56 to Day -3) (All Cohorts) 50
13.3 Admission to Quarantine (Day -2/ Day -1) 51
13.3.1 Day -2 OR Day -1 51
13.3.2 Day -1 51
13.4 Viral Challenge (Day 0) 52
13.4.1 Pre- Viral Challenge 52
13.4.2 Viral Challenge 53
13.4.3 Post- Viral Challenge 53
13.5 Quarantine Days 1 to 7 53
13.5.1 Daily 53
13.5.2 Day 3 53
13.5.3 Day 7 53
13.6 Discharge from Quarantine (Day 8) 54
13.6.1 Prior to discharge (Day 8) 54
13.6.2 Days 9-11 54
13.7 Withdrawal Visit 54
13.8 Follow up Visit 54
13.8.1 Day 28 (± 5 days) 54
13.9 Subject Discontinuation (Lost to Follow Up) 55

14. DEFINITIONS OF ILLNESS AND INFECTION 56
14.1 Infection 56
14.1.1 Laboratory-Confirmed Influenza Infection 56
14.1.1.1 Challenge Virus Seroconversion 56
14.1.1.2 Seroprotection 56
14.1.2 Viral Replication 56
14.1.3 Viral Shedding 56

14.2 Illness ... 56
14.2.1 ILI (Influenza-like Illness) ... 56
14.2.2 Laboratory confirmed ILI ... 56
14.2.3 Febrile Illness ... 56
14.2.4 Lower Respiratory Tract Illness ... 57
14.2.5 Upper Respiratory Tract Illness ... 57
14.2.6 Systemic Illness ... 57
14.2.7 Sub-clinical Infection ... 58
14.2.8 Non-sick and uninfected ... 58
14.3 Seroconversion and Seroprotection ... 58

15. CLINICAL ASSESSMENTS ... 59
15.1 Medical History ... 59
15.2 Prior and Concomitant Medications ... 59
15.3 Height, Weight, BMI and Waist Measurement ... 59
15.4 Physical Examination ... 59
15.4.1 Complete Physical Examination ... 59
15.4.2 Directed Physical Examination ... 60
15.5 Vital Signs ... 60
15.6 Temperature ... 61
15.7 12-Lead Electrocardiogram ... 61
15.8 Spirometry ... 61
15.9 Alcohol Breath Test ... 62
15.10 Tissue Count and Mucus Weight ... 62
15.11 Symptom Diary Card ... 62
15.12 Adverse Events ... 63

16. LABORATORY ASSESSMENTS ... 64
16.1 Blood ... 64
16.1.1 Clinical Safety ... 64
16.1.1.1 Haematology ... 64
16.1.1.2 Clinical Chemistry ... 64
16.1.1.3 Coagulation ... 65
16.1.1.4 Cardiac Enzymes ... 65
16.1.1.5 HIV, Hepatitis A, B and C ... 65
16.1.1.6 Pregnancy Test ... 65
16.2 Urine ... 65
16.2.1 Urinalysis ... 65
16.2.2 Class A Drugs and Cotinine Screen ... 66
16.2.3 Pregnancy test ... 66

17. PATHOGENICITY AND VIRULENCE SAMPLES AND ANALYSIS ... 67
17.1 Blood ... 67
17.1.1 Sera for Challenge Virus Serology ... 67
17.1.2 Additional Sera ... 67
17.1.3 PAXgene (RNA & DNA) ... 67
17.1.4 PBMC ... 67
17.2 Nasopharyngeal Swab ... 68
17.2.1 Respiratory Virus Panel ... 68
17.2.2 Rapid diagnostic test ... 68

18. ADVERSE EVENTS ... 69
18.1 Definitions ... 69

18.1.1 Adverse Event 69
18.1.2 Serious Adverse Events 69
18.1.3 Clinically Significant Laboratory Abnormalities and Other Abnormal Assessments 70
18.2 Recording Adverse Events 71
18.2.1 Description 71
18.2.1.1 Onset and End 71
18.2.1.2 Seriousness 71
18.2.1.3 Intensity and Severity 71
18.2.1.4 Causality 71
18.2.1.5 Action Taken 72
18.2.1.6 Outcome 72
18.2.1.7 Follow up 72
18.2.2 Pregnancy 73
18.3 Reporting SAEs.... 73
18.3.1 Research Ethics Committee 74
18.4 Post-Study Obligations 74

19. STATISTICAL METHODS AND PLANNED ANALYSES 75

19.1 Study Populations 75
19.1.1 Safety Analysis Set 75
19.1.2 Virulence and Pathogenicity Analysis Set 75
19.1.3 Per Protocol (PP) Analysis Set 75
19.2 Data Management 75
19.3 Data Coding 76
19.4 Sample Size 76
19.5 Interim Analysis 77
19.6 Randomisation (Cohort 1) 77
19.7 Statistical Analysis Plan 77
19.7.1 Subject Accountability 77
19.7.2 Protocol Deviations 78
19.7.3 Subgroup Analysis 78
19.7.4 Demographic and Baseline Characteristics 78
19.7.6.1 Viral Load AUC and Daily AUC 78
19.7.6.2 Peak value, delta peak value, time to peak, duration, time to resolution from peak, and daily incidence of the Challenge Viral load, measured by nasopharyngeal swab qPCR, after Viral Challenge 79
19.7.6.3 The peak value, delta peak value, time to peak, duration, time to resolution from peak, and daily incidence of the Challenge Viral load, measured by nasopharyngeal swab cell culture, after Viral Challenge 80
19.7.6.4 The peak value, delta peak value, time to peak, duration, time to resolution from peak, and daily incidence of the Challenge Viral load, measured by nasopharyngeal swab qPCR, after Viral Challenge. 80
19.7.6.5 Proportion of subjects with laboratory-confirmed influenza infection ...80
19.7.6.6 Proportion of subjects with viral shedding 80
19.7.6.7 Proportion of subjects with viral replication 80
19.7.6.8 AUC and daily AUC of total symptom score, from post *inoculum to the last day of* Quarantine. 80

19.7.6.9 AUC and daily AUC of component symptom scores, from post inoculum to the last day of Quarantine 80
19.7.6.10 AUC and daily AUC of total symptom score using individual symptom scores Grade 2 or higher, from post inoculum to the last day of Quarantine 81
19.7.6.11 AUC and daily AUC of component symptom scores using individual symptom scores Grade 2 or higher, from po*st inoculum to the last day of* Quarantine 81
19.7.6.12 The proportion (on any occasion, and on two separate occasions), duration, peak value, time to peak, time to resolution and daily incidence, of any symptoms Grade 2 or higher, after Viral Challenge .81
19.7.6.13 The proportion (on any occasion, and on two separate occasions), duration, peak value, time to peak, time to resolution, and daily incidence, of the URT and LRT components of symptoms grade 2 or higher after Viral Challenge 82
19.7.6.14 The proportion (on any occasion, and on two separate occasions), duration, peak value, time to peak, time to resolution and daily incidence, of symptoms of any grade after Viral Challenge 82
19.7.6.15 The proportion (on any occasion, and on two separate occasions), duration, peak value, time to peak, time to resolution and daily incidence, of the URT and LRT components of symptoms of any grade after Viral Challenge 82
19.7.6.16 The proportion, AUC, peak value, duration and time to resolution of febrile illness after Viral Challenge 82
19.7.6.17 The proportion of subjects with influenza-like illness (ILI), laboratory confirmed ILI, Upper Respiratory Tract Illness (URTI), Lower Respiratory Tract Illness (LRTI), Systemic Illness (SI), Non-sick but infected subjects (subclinical infection), Non-sic and uninfected, after Viral Challenge 83
19.7.6.18 Proportion of subjects with seroconversion to Viral Challenge 83
19.7.6.19 Proportion of subjects with seroprotection to Viral Challenge 83
19.7.6.20 Mucus weight assessed by paper tissue weights and tissue count 83
19.7.7.2 12-Lead ECG 84
19.7.7.3 Laboratory Parameters 84
19.7.7.4 Vital Signs 84
19.7.7.5 Physical Examination 84
19.7.8 Combined Cohort Analysis 85

20. STUDY MANAGEMENT AND RESPONSIBILITIES 86
20.1 Ethics Review 86
20.2 Deviations from the Protocol and Protocol Amendments 86
20.2.1 Protocol Deviations 86
20.2.2 Serious Breach 86
20.2.3 Protocol Amendments 86
20.3 Study Discontinuation 86
20.4 Study Records Retention and Direct Access to Source Documents 87
20.5 Sponsor Responsibilities 87
20.6 Monitoring and Auditing 87
20.7 Investigator Responsibilities 87
20.7.1 Ethical Considerations 87
20.7.2 Informed Consent 88
20.8 Laboratory Certification and Normal Values 89

20.9 Information for the Subject's General Practitioner 89
20.10 Compensation and Expenses 89
20.11 Non-Protocol Research 89
20.12 Liability and Insurance 89
20.13 Audits and Inspections 89
20.14 Study Termination 89

21. DISCLOSURE OF DATA 91

21.1 Subject Confidentiality 91
21.2 Sponsor Confidentiality 91
21.3 Publication Policy 91
21.4 Study Documentation 91
21.4.1 Source Documents 91

22. REFERENCES 92

23. APPENDICES 94

List of Appendices

Appendix 1: RVL Symptom Diary Card 95
Appendix 2: Wisconsin Symptom Diary Card 97
Appendix 3: Common Terminology Criteria for Adverse Events 98
Appendix 4: DAIDS Table for Grading the Severity of Adult and Pediatric Adverse Events 99

List of Tables

Table 7-1: Samples for RVL Discovery Programme 30
Table 8-1: Challenge Virus Titre for Cohort 1 32
Table 8-2: Challenge Virus Titre for Cohort 2 33
Table 8-3: RVL Study Stopping Criteria 34
Table 13-1: Time and Events Schedule 48
Table 18-1: SAE Reporting Requirements 74

List of Figures

Figure 1: Study Design for Subjects 32

3. PROTOCOL SYNOPSIS

Sponsor	Retroscreen Virology Limited (RVL)
RVL Protocol	RVL-vCS-003
Study Title	A Randomised Study to Characterise Influenza A/Perth/16/2009 (H3N2) Virus
Phase	Exploratory
Study Design	Randomised double blind study
Study Population	Male and/or female subjects aged 18-64 years inclusive who meet the study eligibility criteria.
Sample Size	Up to four groups of subjects will be inoculated with the allocated dilution of Challenge Virus within a Quarantine environment.
Challenge Virus	A/Perth/16/2009(H3N2)
Route	The inoculum will be delivered intranasally in drops by pipette
Number of Titre Groups	Cohort 1 (18 – 45 years): Up to 4 groups of subjects within this cohort will be inoculated with the allocated Challenge Virus dilutions (titres) within a Quarantine environment.<br><br>Cohort 2 (18 – 64 years): Up to 28 subjects will be inoculated with the identified titre from Cohort 1.
Infectious Titres	The dilutions to be used in cohort 1 will be confirmed after the ferret study that assesses different dilutions. A review board will confirm the dilutions before proceeding with the human challenge.<br>Following the analysis of the cohort 1, an identified titre from this cohort will be used in cohort 2.
Primary Objective	To determine the optimum infectious titre of A/Perth/16/2009 (H3N2) Challenge Virus in volunteers 18 to 64 years of age.
Primary Endpoint	Area under the curve (AUC) of the Challenge Viral load post Viral Challenge to the last day of Quarantine.
Safety Objectives	To assess the safety of the Challenge Virus inoculum in various age groups.

Safety Endpoints	The incidence (number and percentage) of emergent adverse events (AEs) which are not consistent with a mild to moderate influenza infection overall and by time period from pre Viral Challenge to the last scheduled assessment, for example LFTs and spirometry.<br>• Change from pre Viral Challenge baseline in electrocardiograph (ECG) by timepoint to the last scheduled assessment.
Secondary Objectives	• To assess the interaction of the Challenge Virus inoculum on the human body<br>• To evaluate post Viral Challenge, the:<br>o Virus load from the nasopharyngeal mucosa<br>o Virus shedding from the nasopharyngeal mucosa<br>o Symptoms of respiratory viral illness, including dichotomous variables<br>• To evaluate the pathogenicity and virulence of the identified optimum Challenge Virus titre in various age groups.
Secondary Endpoints	• Vital signs<br>• Temperature<br>• Spirometry<br>• Composite symptom score<br>• Tissue count and mucus weight<br>• Concurrent medications<br>• Laboratory safety blood tests<br>• Viral load<br>• Virus shedding
Exploratory Objectives	• To propagate the Viral Challenge stock from infected subjects' nasopharyngeal swab samples<br>• To investigate the immune correlates of protection from challenge<br>• To investigate the cytokine responses to infection<br>• To evaluate the total virus load from the throat mucosa and the nasopharyngeal mucosa post Viral Challenge.
Exploratory Endpoints	• Infected subject swab samples for propagation of a new stock of Challenge Virus.<br>• Immunological biomarkers of protection from infection and/or abrogation of disease.<br>• Cytokines induced (or down regulated) post infection.<br>• Viral load parameters in nasopharyngeal swab samples:<br>o AUC, AUC (daily)<br>o Absolute peak, time to peak, delta peak<br>o Virus shedding, duration, time to resolution.

Discovery Objectives	To collect and process appropriately consented samples for storage as per the RVL discovery programme from Day -2/Day -1 through to the last scheduled follow up date.
Discovery Endpoints	Storage of linked-anonymised samples as per the RVL discovery programme under the HTA licence for exploratory and other analyses.
Screening Phase	• Between Day -80 and Day -3 prior to the day of inoculation with Challenge Virus (Day 0) volunteers of who do not have serosuitability results in the RVL database will be invited to a Pre-Screening visit during which their serosuitability will be assessed.<br>• Between Day -56 and Day -3 prior to the day of inoculation with Influenza Challenge Virus (Day 0) volunteers will attend a study specific screening (SSS) visit during which their eligibility to participate will be assessed<br>• If the time between SSS (Pre-Screening Visit for cohort 2) and entry into Quarantine exceeds 54 days (but is no more than 80 days) as a result of unexpected delays, subjects do not require re-screening.
Quarantine Phase	• Eligible subjects will be admitted to the Quarantine Unit prior to the day of inoculation with the Challenge Virus and will be assigned to one of the virus dilution groups.<br>• Intranasal inoculation will be performed on Day 0.<br>• Subjects will remain in the Quarantine Unit until their planned discharge on Day 8, however, if symptoms have not resolved on the planned discharge day or there is evidence of continuing viral infection, subjects may be required to extend their stay in isolation.
Follow Up Phase	• Subjects will attend a follow up visit on Day 28 (± 5 days) post Viral Challenge.<br>• Each subject will be assessed by the investigating team for on-going symptoms and AEs.<br>• Any AEs that are unresolved at the Day 28 (± 5 days) post-challenge Follow up Visit may necessitate further follow up visits up to 60 days post Viral Challenge, with onward referral to an appropriate medical team as required.
Duration of Participation	From initial screening to the final scheduled follow-up visit, the duration of participation for a subject could be up to 145 days (5 months).
End of Trial	The end of the study is defined as last subject last visit (LSLV) on Day 28 (± 5 days).
Total Trial Duration	Study duration for First Subject First Visit (FSFV) to LSLV is expected to be up approximately 12 months with an additional six months for data management, data lock, analysis, reporting, and archiving.<br>For subjects of cohort 1 the Study-Specific Screening (SSS) Visit will be considered FSFV. For subjects of cohort 2 the Pre-Screening Visit will be considered FSFV.

Sample Size	No formal sample size calculation has been performed for this exploratory phase study; the sample size is consistent with earlier virus characterisation studies conducted at RVL.
Analysis	Prior to cohort 2 (18 – 64 years), analysis and unblinding of cohort 1 (18-45) will be undertaken to identify the optimum challenge virus titre for use in cohort 2.<br><br>All endpoints, unless otherwise specified, will be analysed and presented separately for both cohorts 1 and 2.<br><br>The study will be summarised in terms of descriptive statistics, no formal statistical comparisons of groups are planned.<br><br>Continuous variables will be summarised using number of observations, mean, standard deviation, median, lower quartile, upper quartile, minimum and maximum values. Categorical variables will be summarised using proportions (counts and percentages).<br>• For the safety analysis the primary concern will be the Safety Analysis Set<br>• For the efficacy analysis the Virulence and Pathogenicity Analysis Set will be used, with the Per Protocol (PP) population being of secondary concern:<br>• The PP population will only be analysed/presented if the PP and Safety Population differ<br>• Where appropriate, subjects will be grouped and presented against the titre of Challenge Virus they received.<br>• The number of subjects randomised (cohort 1), enrolled, withdrawing (also split by reason for withdrawal) from and completing the study, and the number in each analysis population will be summarised for all subjects and by group.<br>• Any missing data will be accounted for, and their possible impact on the study analysis will be described within the Statistical Analysis Plan (SAP).<br><br>An analysis combining both cohorts 1 and 2 subjects that received the optimum virus titre will be performed. The endpoints for this combined analysis will be similar to those specified for each cohort, and will compare data across age ranges.

4. ABBREVIATIONS

AE	Adverse Event
ALP	Alkaline Phosphatase
ALT	Alanine Aminotransferase
AP	Analytical Plan
APTT	Activated Partial Thromboplastin Time
AST	Aspartate Aminotransferase
AUC	Area Under the Curve
βHCG	Beta-Human Chorionic Gonadotropin
BD	Bis die (2 x daily)
BMI	Body Mass Index
bpm	beats per minute
BP	Blood Pressure
cGMP	Current Good Manufacturing Practice
CI	Chief Investigator
COPD	Chronic Obstructive Pulmonary Disease
CRO	Contract Research Organisation
CRP	C-Reactive Protein
CS	Clinically Significant
CSR	Clinical Study Report
CTCAE	Common Terminology Criteria for Adverse Events
DBP	Diastolic Blood Pressure
DFA	Direct Fluorescence Antibody
DAIDS	Division of Aids
DMID	Division of Microbiology and Infectious Disease.
DMP	Data Management Plan
DNA	Deoxyribonucleic Acid
DPE	Direct Physical Examination
ECG	Electrocardiogram
eCRF	Electronic Case Report Form
FEF	Forced Expiratory Flow
FEV_1	Forced Expiratory Volume in 1 second
FI	Febrile Illness
FSFV	First Subject First Visit
FVC	Forced Vital Capacity
GCP	Good Clinical Practice
GGT	Gamma Glutamyl Transferase or Gamma Glutamyl Transpeptidase
GMP	Good Manufacturing Practice

GP	General Practitioner
h	Hour
HA	Haemagglutinin
HAV	Hepatitis A Virus
HBV	Hepatitis B Virus
HCV	Hepatitis C Virus
HIV	Human Immunodeficiency Virus
HR	Heart Rate
HTA	Human Tissue Authority
HVC	Human viral challenge
ICF	Informed Consent Form
ICH	International Conference on Harmonisation
IFN	Interferon
IgA	Immunoglobulin A
ILI	Influenza Like Illness
IMP	Investigational Medicinal Product
INR	International Normalised Ratio
IUD	Intra-Uterine Device
IUS	Intra-Uterine System
kg	Kilogram
LDH	Lactate Dehydrogenase
LRT	Lower Respiratory Tract
LRTI	Lower Respiratory Tract Infection
LSLV	Last Subject Last Visit
m	Metre
Main REC	Main Research Ethics Committee
MCH	Mean Corpuscular Haemoglobin
MCHC	Mean Corpuscular Haemoglobin Concentration
MCV	Mean Corpuscular Volume
MedDRA	Medical Dictionary for Regulatory Activities
mg	Milligrams
MHRA	Medicines and Healthcare Products Regulatory Agency
MMEF	Maximum Mid Expiratory Flow
mL	Millilitre
mmHg	Millimetres of mercury
MP	Monitoring Plan
mRNA	Messenger Ribonucleic Acid
NA	Neuraminidase

NCS	Not Clinically Significant
NIMP	Non- Investigational Medicinal Product
OTC	Over-the-Counter
PBMC	Peripheral Blood Mononuclear Cell
PCR	Polymerase Chain Reaction
PP	Per Protocol
PT	Prothrombin Time
QA	Quality Assurance
QC	Quality Control
QDS	Quater die sumendus(4 x per day)
qPCR	Quantitative Polymerase Chain Reaction
qRT-PCR	qualitative (Reverse Transcriptase) Polymerase Chain Reaction
RBC	Red Blood Cell
REC	Research Ethics Committee
RNA	Ribonucleic Acid
RR	Respiratory Rate
RT-PCR	Rapid time-polymerase chain reaction
RVAT	Rapid Virus Antigen Test
RVL	Retroscreen Virology Limited
RVP	Respiratory Virus Panel
SAE	Serious Adverse Event
SAP	Statistical Analysis Plan
SAR	Serious Adverse Reaction
SBP	Systolic Blood Pressure
sd	Standard Deviation
SDV	Source Data Verification
SI	Systemic Illness
si-RNA	Small/Short interfering Ribonucleic Acid
SME	Sponsor’s Medical Expert
SMF	Study Master File
SOC	System, Organ, Class
SOP	Standard Operating Procedure
SpO_2	Oxygen saturation
SRT	Systemic Respiratory Tract
SSS	Study Specific Screening
ST	S-T segment of the ECG
SUSAR	Suspected Unexpected Serious Adverse Reaction
T wave	T wave on the ECG

$TCID_{50}$	Tissue Culture Infective Dose (Titre) 50%
TDS	ter die sumendus (3 x daily)
TFT	Thyroid Function Test
TSL	Translational Study Lead
UK	United Kingdom
URT	Upper Respiratory Tract
URTI	Upper Respiratory Tract Infection
USA	United States of America
VIS	Volunteer Information Sheet
WBC	White Blood Cell
WHO	World Health Organisation

5. RVL DEFINITIONS

TERM	RVL DEFINITION
Human Viral Challenge (HVC) Study	A study to determine how a virus and the human body interact. Subjects are isolated in a RVL Quarantine Unit and infected (challenged) with a respiratory virus.
Non-IMP study	A RVL subtype of study where no IMP is administered to subjects, although subjects are challenged with virus.
Investigational Medicinal Product (IMP)	A pharmaceutical form of an active substance or placebo being tested, or to be tested, or used, as a reference in a clinical trial.
Titre	The titre of virus inoculum. The term 'titre' applies to virus inoculum or antibody.
Infectious Titre	The titre of virus inoculum producing viral infection in a subject.
Quarantine Unit	RVL isolation facility for HVC studies.
Quarantine Period	The period of time when clinical trial subjects are isolated in the Quarantine Unit during a HVC study.
Quarantine Group	A group of subjects resident in the Quarantine Unit who are receiving the same dose/ virus titre in a HVC study.
Seroconversion	Seroconversion is defined as a more than or equal to 4 fold increase in antibodies to A/Perth/16/2009(H3N2) from baseline.
Symptom Diary Card	A document in which the subject records his/her assessment of symptoms related to the study indication.
Virulence and Pathogenicity Population	All sero-suitable subjects receiving virus inoculum.
Per Protocol (PP) Population	The PP population is defined as all virulence and pathogenicity population subjects who have no major protocol deviations and who complete the Quarantine period up to the final day of Quarantine.
Safety Population	For a non-IMP study, the safety population is defined as all subjects receiving Challenge Virus inoculum. The sero-suitability of the subject for the study, which may only be determined after viral challenge, has no bearing on inclusion of the subject in the safety population.

TERM	RVL DEFINITION
Viral Challenge (or Challenge)	The inoculation of a study subject with virus inoculum. By definition, the day of viral challenge is Day 0.
End of Study	The end of the study is defined as last subject last visit (LSLV).
Chief Investigator (CI)	The CI (or delegate) is responsible for conducting the trial (GCP E6(R1) 1996 Section 6.1.5).
Sponsor's Medical Expert (SME)	An appropriately qualified medical person designated by RVL who will be readily available to advise on trial related medical questions or problems.
Monitor/Monitoring Organisation	The company overseeing progress of the clinical trial and ensuring that it is conducted, recorded, and reported in accordance with the protocol, standard operating procedures (SOPs), GCP and applicable guideline requirements (GCP E6 (R1) 1996 Section 1.38).<br>The monitoring function may be fulfilled by RVL or contracted out to a Contract Research Organisation (CRO).
Study Monitor	The individual assigned by the Monitoring Organisation to perform study-monitoring visits to the study site.

6. INTRODUCTION

6.1 Retroscreen Virology

Retroscreen Virology Ltd (RVL) is an independent specialist research company with its origins in the University of London. RVL primarily conducts studies into antiviral chemotherapy and vaccine development. Potential studies can range from single vaccine immunogenicity to field studies (where people with respiratory virus infections are identified). The company conducts HVC studies in which subjects may receive a drug or placebo before being inoculated (challenged) with a respiratory virus in a controlled quarantine facility.

Over the past decade, RVL has conducted studies with influenza, respiratory syncytial virus and human rhinovirus, and infected over 1200 volunteers in 29 clinical studies, under similar Quarantine environments.

RVL's research has demonstrated proof of concept for novel therapeutics such as DNA vaccines[3], siRNAs[4], and early stage diagnostic platforms[5-7].

6.2 Influenza

The influenza virus is transmitted primarily by droplets or respiratory secretions of infected persons and causes an acute viral disease of the respiratory tract typically characterised by abrupt onset of fever, prostration myalgias, malaise, headache, sore throat, and non-productive cough. The illness is usually self-limiting, with relief of symptoms occurring within 5 to 7 days.

Influenza occurs all over the world with an estimated annual global attack rate of 5-10% in adults and 20-30% in children. It is an important disease because of its ease of communicability, short incubation time, rapid rate of viral mutation, morbidity caused with resultant loss of productivity, severity of complicating diseases, and increased risk of death. Vaccination has been established as the first-line intervention to prevent influenza and its complications. In the UK, the Department of Health currently recommends that people who are at risk of influenza infection or complications are vaccinated at the beginning of each winter. Such people are those with chronic respiratory, cardiovascular, renal, liver or neurological disease, people with diabetes, people who are immunosuppressed, people aged 65 and older, people who work or live in residential care facilities, carers of at-risk people, healthcare and other essential workers and poultry workers[8, 9].

Historically, influenza has been associated with an increased risk of cardiac changes and an estimated 1% risk of myocarditis during Influenza infection. However a study of cardiac changes during influenza infection using sensitive cardiac indicators has indicated that the actual risk is likely to be substantially less, although skeletal muscle injury is relatively common[10].

Influenza viruses belong to the Orthomyxoviridae family, have a single-stranded segmented RNA genome, and are classified into types A, B, and C on the basis of their core proteins. Influenza A viruses infect a range of mammalian and avian species whereas Influenza B and C viruses mainly affect humans. Only Influenza types A and B cause human disease of any concern, Influenza type C infections cause a mild respiratory illness and are not thought to cause epidemics. [8, 11]

Type A viruses are further subdivided according to their envelope glycoproteins with haemagglutinin (HA) or neuraminidase (NA) activity. The influenza virus undergoes high mutation rates and frequent genetic reassortment leading to variability in HA and NA antigens.

Minor changes in the protein structure of influenza A strains ("antigenic drift") occur frequently and enable the virus to cause repetitive influenza outbreaks by evading immune recognition. Major changes in the influenza type A HA antigen ("antigenic shift") are caused by reassortment from different influenza A subtypes, such as between animal and human subtypes, and in rare events, such shifted viruses can result in strains capable of causing large regional or global pandemic outbreaks. An influenza pandemic is a rare but recurrent event.

The two main classes of antiviral drugs used against Influenza are neuraminidase inhibitors, such as zanamivir (Relenza®) and oseltamivir (Tamiflu®), or inhibitors of the viral M2 protein, such as amantadine (Symmetrel®) and rimantadine (Flumadine®) [12-15]. Unfortunately, strains that show drug resistance to both classes of drug have already emerged and it is possible that extensive use of these antivirals could generate large numbers of resistant viruses.

Mucosal IgA antibodies contribute to resistance against infection, serum antibodies confer protection against clinical disease, and neutralizing antibodies mainly target HA. However, due to antigenic drift and antigenic shift, over time the protective effect of an antibody induced by one strain may be reduced or lost, resulting in individuals being relatively or completely unprotected against new circulating strains[16].

The development of new vaccines and antivirals is therefore urgently required and the experimental Viral Challenge Model allows the rapid conduct of proof of concept studies. RVL has successfully demonstrated that challenge with the Influenza virus can be performed with minimal risk to healthy volunteer subjects [17].

Before conducting clinical trials of vaccines and anti-viral drugs using the HVC model, the virus inoculum to be used (the ‘Challenge Virus’) must be characterised in a separate HVC study, in order to identify the optimal titre of virus inoculum to be used, and determine the safety of the virus and the sample size required for clinical trials.

6.3 The Human Viral Challenge Model

6.3.1 Rationale for the Study Design

Studies using experimental influenza virus infection in human subjects have demonstrated that adult volunteers can be infected by nasal inoculation, and experimental infection is safe and not associated with transmission to contacts [18].

The model has been very useful in evaluating vaccines and new treatments in volunteers in the 18 - 45 age group [3, 6, 19], and will be invaluable to be extended to volunteers up to 64 years of age [20, 21].

6.3.2 Potential Risks and Benefits for the Volunteers

No potential benefits to the volunteer are expected from participation in this study. The risks to volunteers in this study may include events that have not been observed with the Viral Challenge strain.

Based on RVL experience with other Influenza challenge strains, normal healthy volunteers are expected to follow a predictable pattern of clinical symptoms and viral shedding that usually ceases five to seven days after Viral Challenge. Influenza symptoms are not expected to differ in subjects aged 46 to 64 years of age compared to younger adults[22, 23]. Furthermore, the initiation of licensed influenza antiviral medication to subjects with evidence of continuing viral infection before discharge ensures that the infection is cleared and resolved.

Side effects of licensed antiviral medications can include nausea, vomiting and diarrhoea, rarely skin/hypersensitivity reactions, and neuropsychiatric events. In addition, the requirement for negative Rapid diagnostic test for Influenza prior to discharge ensures that volunteers have a low risk of transmitting influenza infection in the community.

There are no known previous HVC studies involving this strain of the Challenge Virus. However, prior to the study RVL will have the following information relating to the Challenge Virus:

- Tissue Culture Infective Dose (Titre) 50% ($TCID_{50}$/mL)
- Quantitative Polymerase Chain Reaction (qPCR)
- HA/50µl

The known risks to participants in this study have been addressed in the study eligibility criteria (See Section 9.2 and 9.3) and are as follows:

- Influenza virus – associated risk cardiac changes
- Allergy to chicken egg, which is used in the virus manufacture

Qualified doctors and nurses in the Quarantine Unit will manage any symptoms that develop during the study.

6.3.2.1 Risk of transmission of Challenge Virus

Although A/Perth/16/2009(H3N2) virus will be present in a subject’s nose for several days after infection, it is unlikely that there will be sufficient virus in the nose for subjects to transmit it to their close contacts once they leave the Quarantine Unit. It is expected that the A/Perth/16/2009(H3N2) infection will be in an adult’s nose for less time (several days) than the time the subject spends in the Quarantine Unit.

Subjects’ nasopharyngeal swab samples will be tested using a rapid diagnostic test (Section 17.2.2) on Day 7, prior to planned discharge on Day 8, and subjects may not be discharged if there is evidence of continuing viral infection.

However, to reduce the risk of passing A/Perth/16/2009(H3N2) to others, for 28 days post inoculation, subjects will be asked to avoid contact with any person who:

- is a child under one year old
- has known immunodeficiency
- is receiving immunosuppressant medication.
- is undergoing or soon to undergo cancer chemotherapy.
- has been diagnosed with emphysema, chronic obstructive pulmonary disease (COPD), or other severe lung disease and resides in a nursing home.
- has received a bone marrow or solid organ transplant.

There are minor risks and discomforts associated with the study procedures that are described in the Volunteer Information Sheet (VIS), and will be included in discussion of the study as part of the informed consent procedure (Section 20.7.2.).

6.3.2.2 Safety Measures

Subjects will be assessed on Day 7 for the presence of a continuing viral infection, hence antiviral medication (e.g., oseltamivir [Tamiflu®]) will be administered only if continuing viral infection is detected.

If indicated, antiviral medication (e.g., oseltamivir [Tamiflu®]) will be administered twice-daily for five days as per the product licence.

Subjects who still have influenza symptoms following the Day 7 assessments (including any febrile symptoms) may be asked to remain in the Quarantine Unit through study Day 8 or at the CI's discretion until febrile illness or symptoms have resolved. A subject must be asymptomatic and have a negative Influenza rapid diagnostic test (Section 17.2.2) before he/she can be discharged from the Quarantine Unit.

7. STUDY OBJECTIVES AND ENDPOINTS

7.1 Primary Objective

To determine the optimum infectious titre of A/Perth/16/2009 (H3N2) Challenge Virus in volunteers 18 to 64 years of age.

7.1.1 Primary Endpoint

The primary endpoint is the AUC of A/Perth/16/2009(H3N2) challenge viral load post Viral Challenge to the last day of Quarantine.

7.2 Safety Objective

To assess the safety of the Challenge Virus inoculum.

7.2.1 Safety Endpoints

- The incidence (number and percentage) of emergent AEs which are not consistent with a mild to moderate influenza infection overall, and by time period from pre-Viral Challenge to the last scheduled assessment.
- Change from pre- Viral Challenge baseline in ECG parameters and waves by timepoint to the last scheduled assessment (e.g., T waves, and ST).

7.3 Secondary Objectives

- To assess the interaction of the Challenge Virus inoculum on the human body
- To evaluate post - Viral Challenge, the:
  - Virus load from the nasopharyngeal mucosa
  - Virus shedding from the nasopharyngeal mucosa
  - Symptoms of respiratory viral illness, including dichotomous variables.

To evaluate the pathogenicity and virulence of the identified optimum Challenge Virus titre in various age groups.

7.3.1 Secondary Endpoints

Vital signs

Pre Viral Challenge baseline by timepoint to last scheduled assessment:

- Change in absolute values

Temperature

Post Viral Challenge to the end of Quarantine:

- AUC
- Time from peak to resolution (time from peak temperature to the timepoint after the last time that the temperature is above 37.9ºC).
- Pyrexia- mean duration (hours) (time from initially exceeding 37.9ºC to the timepoint after the last time that the temperature is above 37.9ºC).

- Pre Viral Challenge by time to the end of Quarantine:
- Peak change.

Spirometry

Change compared with pre Viral Challenge baseline by time point to last scheduled assessment

Composite Symptom Score

Post Viral Challenge to the last day of follow up:

- Total AUC
- Daily AUC.

Post Viral Challenge to the end of Quarantine:

- AUC for URT symptoms
- AUC for LRT self-reported symptoms
- Peak sum
- Time-to-peak for sum of URTI symptoms.

From day of peak symptoms, to resolution (to the end of Quarantine):

- Time to resolution.

Tissue count and mucus weight

Post Viral Challenge to the end of Quarantine:

- Total number of tissues used
- Total mucus weight
- Total weight of mucus over sum of symptoms scores.

Concurrent medications

Use associated with viral infection from viral challenge to the last scheduled assessment.

Laboratory safety blood tests

Pre viral challenge baseline by timepoint to last scheduled assessment:

- Maximum change in absolute values.

Viral load

Post viral challenge to resolution:

- AUC.

Post viral challenge to end of Quarantine:

- Daily AUC
- Absolute peak value per group
- Delta peak (difference between the viral load on day of peak (for each subject) above the viral load on the first detection of virus shedding (for that subject))
- Time to peak per group
- Difference in time to peak per group.

Virus Shedding

Post viral challenge to the timepoint after the last positive virus detection (to end of Quarantine):

- Duration
- Comparison of the duration
- Time to resolution.

Day of peak virus load to the timepoint after the last positive virus detection post viral challenge (to end of Quarantine):

- Comparison of the time to resolution.

7.3.2 Comparison of Optimum Challenge Virus Titre between Cohorts

Secondary endpoints as delineated in Section 0 will be compared between different age groups.

7.4 Dichotomous Objectives

- To evaluate the difference in incidence in the inoculated groups of seroconversion, viral replication, Influenza infection, moderate and severe symptoms and illness.

7.4.1 Dichotomous Endpoints

- The incidence in the inoculated groups of:
- Laboratory confirmed Influenza Infection
- Challenge Virus seroconversion
- Viral replication
- Viral shedding
- Laboratory confirmed Influenza-like Illness (ILI)
- Influenza-like Illness (ILI)
- Upper Respiratory Tract Illness (URTI)
- Lower Respiratory Tract Illness (LRTI)
- Febrile Illness (FI)
- Systemic Illness (SI)
- Non-sick but infected subjects (subclinical infection)
- Non-sick and uninfected.

The proportion of subjects in the inoculated groups, post Viral Challenge to the last day of follow up with:

- any Grade 2 or worse symptoms
  - on any occasion
  - on two separate occasions
- any Grade 2 or worse URT symptoms
  - on any occasion
  - on two separate occasions
- any Grade 2 or worse LRT symptoms
  - on any occasion
  - on two separate occasions.

7.5 Exploratory Objectives

- To propagate the viral challenge stock from infected subjects' respiratory samples, (specific consent will be requested for the use of virus isolated from samples to propagate new viral challenge stock).
- To investigate the immune correlates of protection from challenge.
- To investigate the cytokine responses to infection.

7.5.1 Exploratory Endpoints

- Collection of infected subject swab samples for propagation of a new stock of Challenge Virus

7.6 Discovery Objectives

To collect and process appropriately consented samples for storage as per the RVL discovery programme from Day -2/Day -1 through to the last scheduled follow up date.

- Collection of pre and post viral challenge sera for investigation of immunological biomarkers of protection from infection and/or abrogation of disease.
- Investigation of the cytokines induced (or down regulated) post infection.

7.6.1 Discovery Endpoints

Storage of linked-anonymised samples as per the RVL discovery programme under the HTA licence for exploratory and other analyses as follows:

Table 7-1: Samples for RVL Discovery Programme

Type of sample	Proteomics & inflammatory markers	Transcriptomics	Genomics	Epigenetics	Other: future usage / exploratory analyses [a]
Blood-PBMC	X	X	X	X	X
Blood-PAXgene		X	X	X	X
Nasopharyngeal swab					X
Serum	X				X

[a] All samples taken from this trial maybe entered into the biobank, for those subjects that have consented for future usage.

Specific DNA consent will be required for the use of a sample for optional DNA analysis.

8. STUDY DESIGN

The study will characterise Influenza A/Perth/16/2009(H3N2) virus in two cohorts.

The first cohort (cohort 1), including up to 28 subjects between 18 and 45 years, will be studied in a randomised, double blind arm of the study.

The second cohort (cohort 2), including up to 28 subjects between 18 and 64 years, will be studied in an open-label extension arm of the study. The Challenge Virus titre to be used in cohort 2 will be identified following an interim analysis of the data from cohort 1.

The study design in Figure 1 shows the three sequential phases to both stages of the study, (1) Screening Phase, (2) Quarantine, and (3) Follow-up.

Subjects will be identified either from RVL's volunteer database or RVL's approved recruitment channels. If there is no serum sample from the volunteer in our data set, the volunteer will attend an extra visit (Pre-Screening) for a serum sample to be obtained.

To confirm serosuitability, a blood sample for A/Perth/16/2009(H3N2) antibody determination may be taken from all subjects at the Pre-screening Visit (Day -80, cohort 2 only), SSS Visit (Day -56 to Day -3) and on entry to Quarantine.
Following confirmation of serosuitability, each subject will:

- be inoculated with Challenge Virus on Day 0
- be discharged from the Quarantine Unit on Day 8
- return to the clinic for a follow up visit on Day 28 (± 5 days).

If appropriate, subjects may reside in the Quarantine Unit for a further night or longer before discharge (see Section 8.2).

Figure 1: Study Design for Subjects

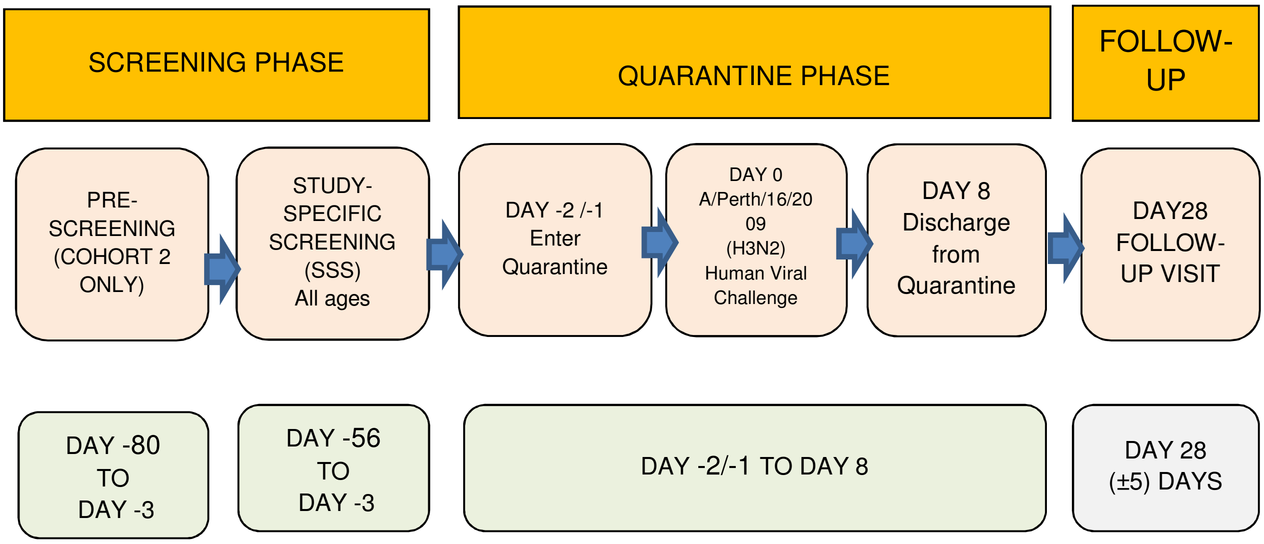

8.1 Screening Phase

8.1.1 Pre-Screening Visit (Cohort 2 only)

For volunteers without a serology sample in RVL database, a Pre-Screening Visit (from Day – 80) will be performed to assess initial serosuitability prior to the SSS visit. Serosuitable subjects will then attend an SSS visit for assessment of their eligibility to participate in the study.

8.1.2 Study-Specific Screening Visit (All Cohorts)

Between Day - 56 and Day -3 prior to the day of inoculation with the Challenge Virus (Day 0), sero-suitable volunteers will attend SSS for assessment of their eligibility to participate in the study.

If, as a result of unexpected delays, the time between SSS and admission into Quarantine /inoculation exceeds 54 days (but is no more than 80 days), then subjects will not need to be re-screened.

8.2 Quarantine Phase

The Quarantine phase will start on admission to the Quarantine Unit (Day-2/Day-1). On admission to and during the Quarantine period, subjects will be observed and tested for signs of concurrent infections.

For cohort 1 different titres of Challenge Virus will be given to up to 4 groups of up to 8 subjects per group as shown in Table 8-1.

Table 8-1: Challenge Virus Titre for Cohort 1

Challenge Virus Titre	Maximum number of volunteers per group
Titre 1	8
Titre 2	8
Titre 3	8
Titre 4	8

The Challenge Virus titre to be used in cohort 2 will be confirmed after an interim analysis has been conducted on results of cohort 1. For subjects of cohort 2, 1 titre of Challenge Virus will be given to up to 28 subjects as shown in Table 8-2.

Table 8-2: Challenge Virus Titre for Cohort 2

Challenge Virus Titre	Maximum number of volunteers per group
Titre X*	28

*Challenge Virus titres to be decided based on data from cohort 1.

Intranasal inoculation with the Challenge Virus will be performed on Day 0 after which subjects will remain in the Quarantine Unit until the day of planned discharge (Day 8).

RVL staff will be in attendance in the Quarantine Unit, to monitor the subjects' safety and wellbeing and to conduct the study procedures and assessments shown in
Table 13-1: Time and Events Schedule, and described in Section 13.

Subjects will be discharged from the Quarantine Unit on Day 8, providing they have a negative influenza rapid diagnostic test (Section 17.2.2) and are free of symptoms.

However, if the scheduled, pre-discharge rapid diagnostic test is positive or the subject remains symptomatic, the subject may need to stay in Quarantine longer (e.g., up to Day 15). The length of extended stay will be at the CI's discretion.

8.3 Follow up Phase

8.3.1 Day 28 Visit (± 5 days)

Subjects will return to the clinic for the End of Study Follow up Visit on Day 28 (± 5 days).

At this clinic visit, blood samples will be taken and safety assessments will be performed. A nasopharyngeal swab will be collected for viral load assessment ($TCID_{50}$ and qPCR).

Subjects will be assessed for on-going symptoms and AEs. Any AEs that are unresolved at this follow up visit may necessitate further follow up visits at the CI's discretion.

8.4 Duration of the Study

From initial screening to the final scheduled follow-up visit, the duration of participation for a subject could be up to 145 days (5 months). Any AEs that are unresolved at the Day 28 Follow up Visit (± 5 days) may necessitate further follow up visits.

The duration of the study from the First Subject First Visit (FSFV) to the Last Subject Last Visit (LSLV) will be approximately 12 months. For subjects of cohort 1 the Study-Specific Screening visit will be considered FSFV. For subjects of cohort 2 the Pre-Screening Visit will be considered FSFV.

The duration of the trial inclusive of data management, data lock, analysis, reporting, and sample and document archiving will be approximately 18 months.

8.5 End of Study

The end of the study is defined as the LSLV.

As a duty of care and in compliance with ICH-E6, it may be necessary to follow up a subject beyond the last scheduled visit for the trial. If further safety visits are required after this (e.g., for repeat spirometry or laboratory tests), such follow up visits will be arranged at the CI's discretion and will not be considered part of the trial data, unless this represents follow up and closure on an AE or SAE identified during the trial period.

Any AEs that are unresolved at the Day 28 (± 5 days) post Viral Challenge Follow up Visit may necessitate further follow up visits up to 60 days post Viral Challenge, with onward referral to an appropriate medical team as required.

8.6 Stopping Criteria

The CI and the Sponsor's Medical Experts (SMEs) will perform safety reviews on available clinical and virology data as appropriate during the Quarantine period.
RVL reserves the right to temporarily suspend or discontinue the study for any reason. In addition, the study may be stopped at any time if, in the opinion of the SME, safety data suggest that the medical safety of the subjects is being compromised.

At any point during this study, the clinical status may present in the following forms:

Table 8-3: RVL Study Stopping Criteria

Status	Criterion	Procedure
1	A report has been received of one (or more) serious adverse events (SAEs) in any one (or more) subject(s).	If such a status occurs at any point during the study, the CI and SME will review the data and make decisions on study continuation or termination. Subject follow up should continue until resolution or stabilisation of AEs.

Status	Criterion	Procedure
2	No SAEs have been reported but an overall pattern of clinical changes or symptoms exist, which may appear minor or moderate in terms of individual AEs, but which collectively represents a concern for safety.	If such a status occurs at any point during the study then the CI and SME will review the data and make a decision on study continuation or termination. Subject follow up should continue until resolution or stabilisation of AEs.
3	Virus-related SAEs or virus-related AEs of clinical concern have been reported following HVC.	Any AEs that are unresolved at the Day 28 (± 5 days) post Viral Challenge Follow up Visit may necessitate further follow up visits up to 60 days post Viral Challenge, with onward referral to an appropriate medical team as required

The CI will promptly inform the REC if the study is suspended or terminated for safety reason(s), and describe the reason(s) for the action.

Premature discontinuation of the study is described in Section 20.3.

9. STUDY POPULATION

Eligible subjects will be males and/or females aged 18 to 64 years inclusive, who meet the eligibility criteria described in Sections 9.2 and 9.3.

Potential subjects will be identified from RVL's volunteer database. To confirm serosuitability, a blood sample for A/Perth/16/2009(H3N2) antibody determination may be taken from all subjects at the Pre-screening Visit (Day -80, cohort 2 only), SSS visit (Day -56 to Day -3) and on entry to Quarantine.

If volunteers are later found not to be sero-suitable for the study, they will be excluded from certain analyses sets, to be defined at the time of analysis.

9.1 Number of Subjects

Once volunteers are invited in Quarantine, up to 28 volunteers of cohort 1 and up to 28 volunteers of cohort 2 may be challenged with the virus. Additional subjects that remain eligible on the Challenge Day may be discharged on Day 0 without being inoculated.

Approximately six subjects of cohort 1 and eight subjects of cohort 2 will be held as reserves (more may be invited to attend Quarantine as required).

9.1.1 Cohort 1

Subjects of cohort 1 will be randomised to one of up to four groups of up to 8 subjects per group (see Table 8-1). Each group will be administered one of up to four different inoculum titres (see Section 8.2).

9.1.2 Cohort 2

Subjects of cohort 2 will receive Challenge Virus inoculum as one group (see Table 8-2).

9.2 Inclusion Criteria

Subjects must meet all of the following inclusion criteria to be eligible for participation in the study	
1	Age 18 to 64 years, inclusive.
2	In good health with no history of major medical conditions from medical history, physical examination, and routine laboratory tests as determined by the Investigator by a screening evaluation.
3	A total body weight ≥ 50 kg and a BMI of >18. If the BMI is above 30 the subject may be included if the waist measurement is less than 102 cm (male), or less than 88 cm (female)

Subjects must meet all of the following inclusion criteria to be eligible for participation in the study	
	(a) Male subjects must use highly effective contraception consisting of two forms of birth control (one of which must be a barrier method) starting at entry to Quarantine and continue until the Day 28 Follow up Visit.<br>(b) In addition, male subjects must not donate sperm following discharge from Quarantine until the Day 28 Follow up Visit.<br>(c) Female subjects must be either:<br>• post-menopausal (defined as at least one year documented history without any menses) prior to Screening or<br>• documented status as surgically sterile or post hysterectomy or<br>• if of childbearing potential, must have a negative urine pregnancy test at SSS and must be using highly effective contraception consisting of two forms of birth control (one of which must be a barrier method) starting at entry to Quarantine and continue until the Day 28 Follow up Visit.<br><br>Acceptable forms of effective contraception include:<br>• Established use of oral, injected or implanted hormonal methods of contraception<br>• Placement of an intrauterine device (IUD) or intrauterine system (IUS)<br>• Barrier methods of contraception: Condom or occlusive cap (diaphragm or cervical/vault caps) with spermicidal foam/gel/film/cream/suppository<br>• Male sterilisation (with the appropriate post-vasectomy documentation of the absence of sperm in the ejaculate). [For female subjects on the study, the vasectomised male partner should be the sole partner for that subject]<br>• True abstinence: When this is in line with the preferred and usual lifestyle of the subject. [Periodic abstinence (e.g., calendar, ovulation, symptothermal, post-ovulation methods) and withdrawal are not acceptable methods of contraception]
5	An informed consent document signed and dated by the subject and Investigator.
6	Sero-suitable for Challenge Virus.

9.3 Exclusion Criteria

Subjects who have any of the following exclusion criteria will be excluded from the study	
1	Subjects who have a significant history of any tobacco use at any time (≥ total 10 pack year history, e.g. one pack a day for 10 years).
2	Subjects who have been pregnant within six months prior to the study, or who have a positive pregnancy test at any point in the study.

Subjects who have any of the following exclusion criteria will be excluded from the study	
3	Any history or evidence of any clinically significant cardiovascular, gastrointestinal, endocrinological, haematological, hepatic, immunological, metabolic, urological, neurological, psychotic, renal, and/or other major disease or malignancy, as judged by the medical investigator.
	(a) Eczema/Atopic Dermatitis: subjects with clinically mild eczema/atopic dermatitis may be included at the Investigator's discretion (e.g. if there is no regular use of topical steroids, no eczema in cubital fossa).
	(b) Psoriasis: patients with active psoriasis affecting less than 5% of the body's surface area for the past five years can be included at the Investigator's discretion. (1% of the body area is roughly equivalent to the palm of the subject's hand). Patients with a history of completely resolved guttate psoriasis can be included.
	(c) Depression: Subjects with a diagnosis of a single mild or moderate depressive episode two or more years ago, with good evidence of preceding stressors and which resolved within approximately three months may be included at the Investigator's discretion.
	(d) Hypertension: Subject with a diagnosis of mild hypertension which is adequately controlled by first line of treatment, may be included at the investigator's discretion.
	(e) Osteoarthritis: Subjects with mild condition who require no medication with no functional limitation may be included at the investigator's discretion.
4	Abnormal pulmonary function in the opinion of the Investigator as evidenced by clinically significant abnormalities in spirometry.
5	History or evidence of autoimmune disease or known immunodeficiency of any cause.
6	Subjects with any history of asthma, COPD, pulmonary hypertension, reactive airway disease, or chronic lung condition of any aetiology. (See No. 7 for asthma).

Subjects who have any of the following exclusion criteria will be excluded from the study	
7	A history of childhood asthma before the age of 12 is acceptable provided the subject is asymptomatic without treatment. Patients with a single episode of wheezing after age 12 (lasting less than eight weeks) can be included at the Investigator's discretion provided the episode is more than four years ago and did not require a hospital admission and/or oral steroids.
8	Positive human immunodeficiency virus (HIV), Hepatitis A (HAV), B (HBV), or C (HCV) test.
9	Any significant abnormality altering the anatomy of the nose or nasopharynx.
10	Any clinically significant history of epistaxis (nose bleeds).
11	Any nasal or sinus surgery within six months of inoculation.
12	Recurrent history of clinically significant autonomic dysfunction.
13	Any abnormal laboratory test or ECG, which is deemed by the Investigator(s) to be clinically significant.
14	Confirmed positive test for drugs of abuse deemed by the Investigator to be clinically significant.
15	Venous access deemed inadequate for the phlebotomy and cannulation demands of the study.
16	Any known allergies to the excipients in the Challenge Virus inoculums.
17	Health care workers (e.g. doctors, nurses, medical students and allied healthcare professionals) who work in units with severely immuno-compromised patients (e.g. bone marrow transplant units).
18	Presence of household member or close contact for 28 days post-inoculation who:<br>• is a child under one year old<br>• has known immunodeficiency<br>• is receiving immunosuppressant medication<br>• is undergoing or soon to undergo cancer chemotherapy<br>• has been diagnosed with emphysema, COPD, or other severe lung disease and resides in a nursing home<br>• has received a bone marrow or solid organ transplant.

Subjects who have any of the following exclusion criteria will be excluded from the study	
19	• Evidence of vaccinations within the four weeks prior to Human Viral Challenge<br>• Intention to receive travel vaccination(s) before the Day 28 Follow Up Visit (No travel restrictions will apply after the Day 28 Follow Up Visit).
20	Those employed or immediate relatives of those employed at RVL.
21	Receipt of blood or blood products, or loss (including blood donations) of 450 mL or more of blood, during the 3 months prior to inoculations.
22	• Use within 28 days prior to Human Viral Challenge (Day 0) of nasal steroids<br>• Use within seven days of any other medication or product (prescription or over-the-counter), for symptoms of hay fever, rhinitis, nasal congestion or respiratory tract infection.
23	• Receipt of any investigational drug within three months prior to inoculation<br>• Prior participation in a clinical trial with the same strain of respiratory virus<br>• Participation in a2ny other Human Viral Challenge Study with a respiratory virus within one year prior to the day of inoculation.
24	Receipt of systemic: glucocorticoids, antiviral drugs, or immunoglobulins (Igs) or any other cytotoxic or immunosuppressive drug within six months prior to dosing. Receipt of any systemic chemotherapy agent at any time.
25	• Presence of significant respiratory symptoms existing on the day of challenge or between admission to the unit and inoculation with virus<br>• History suggestive of respiratory infection within 14 days prior to admission to the unit
26	Any other finding that, in the opinion of the Investigator or Sponsor, deems the subject unsuitable for the study.

9.4 Subject Numbering

At first screening visit, subjects will be assigned a RVL subject number that will be used to identify them up to the point of Viral Challenge. A study specific number will be assigned to them just prior to the Viral Challenge.

9.5 Discontinuation of Subject Participation

A subject's participation in this study may be discontinued for any of the following reasons:

- The subject wishes to withdraw from further participation
- Intolerable AEs
- The subject needs to take medication(s) which may interfere with the study assessments

- The subject does not comply with the protocol requirements
- Continuation in the study would be detrimental to the subject's safety in the opinion of the CI
- A clinically significant abnormal laboratory finding(s) which in the opinion of the CI precludes further participation in the study
- Development of intercurrent illness which, in the opinion of the CI would compromise the health of the subject or the study objectives.

Every attempt will be made to encourage subjects who discontinue after Viral Challenge to comply with the study procedures until the Day 28 (± 5 days) Follow Up Visit, in order to ensure their safety and obtain the required study data.

In the event that a subject discontinues prematurely due to an AE or SAE, the subject will be followed up until the AE or SAE resolves or stabilises, or is judged by the CI to be no longer clinically significant. If necessary, the CI may refer the subject onward to an appropriate medical team as required.

9.5.1 Subject Withdrawal

A subject can withdraw from the study at any time, for any reason, without prejudice to his/her future medical care. Subjects must be counselled that early withdrawal from the Viral Challenge isolation part of the trial will be strongly discouraged, as it may pose a risk both to the subject and to his/her contacts.

In the rare event of a subject insisting on early withdrawal during the Viral Challenge isolation stage, he/she will be encouraged to stay, but would be advised of the risk of potential infection in the community and particularly the risks to vulnerable groups (see Section 6.3.2.1).

If the subject agrees, the Day 28 (± 5 days) follow-up safety assessments may be conducted at the CI's discretion.

9.5.2 Reserve Subjects

The decision to replace subjects who have withdrawn or have been discontinued after Viral Challenge will remain the joint responsibility of the CI and Sponsor, and will be discussed on a case-by-case basis.

Eligible subjects will be available as 'reserves' to replace any subjects who are withdrawn between their admission to the Quarantine Unit and the eligibility assessments prior to Viral Challenge.

Reserves may be admitted to the Quarantine Unit after Day -2 providing there is sufficient time to complete all the required pre- Viral Challenge eligibility evaluations.

Replacement subjects will be assigned a new, unique subject identification number and the same allocated, blinded titre of Challenge Virus as the subject being replaced.

Reserve subjects who are not required for a particular group will be allowed to leave the Unit by Day 0 at the latest. Subjects may be invited to come back for a later group if they remain eligible.

Following inoculation, a subject who has been withdrawn from the study due to an AE will not be able to participate in that quarantine group but will be replaced by a reserve volunteer.

10. STUDY RESTRICTIONS

10.1 Concomitant Medications

Medications taken during the 28 days prior to Viral Challenge will be entered in the subject's source documents.

- Use within 28 days prior to HVC (Day 0) of nasal steroids
- Use within seven days of any other medication or product (prescription or over the counter), for symptoms of hay fever, rhinitis, nasal congestion or respiratory tract infection
- Receipt of systemic; glucocorticoids, antiviral drugs, or immunoglobulins (Igs) or any other cytotoxic or immunosuppressive drug within six months prior to dosing
- Receipt of any systemic chemotherapy agent at any time
- Receipt of any investigational drug within three months prior to inoculation
- Regular use of topical steroids for eczema/ atopic dermatitis
- Vaccination within the four weeks prior to HVC
- Intention to receive travel vaccination(s) before the Day 28 Follow-up Visit

The CI will assess the use of any concomitant medications not listed above and exclude a subject if use of the medication is likely to affect his/her suitability to receive the Challenge Virus inoculum.

The Study Physician may give subjects any concomitant medication required for their welfare. Details regarding the medication(s) and the reason for use will be recorded in the source documentation.

Any changes in medications during the study will be recorded in the source documents.

10.2 Contraception

Study restrictions relating to contraception are described in the inclusion criteria (see Section 9.2).

10.3 Other Restrictions

Volunteers will be asked to refrain from the following from 72 hours (three days) before entry to Quarantine until discharge from the Quarantine Unit:

- alcohol
- smoking
- use of other tobacco products

Subjects will also be advised to avoid strenuous activities from 72 hours (three days) before admission to Quarantine until the last study visit.

11. CHALLENGE VIRUS

11.1 Production

The clinical challenge stock will consist of Challenge Virus manufactured under current Good Manufacturing Practices (cGMP) diluted in a cGMP sucrose solution. The Challenge Virus and diluent will have undergone quality testing (identity, appearance, sterility, infectivity, and contaminants) by the manufacturer (Meridian Life Sciences, Memphis, USA) in accordance with standards for licensed live viral vaccines. Related documentation will have been reviewed and approved by RVL's virologists and Quality Assurance (QA) Department.

The A/Perth/16/2009(H3N2) virus inoculum will have been prepared by an approved subcontractor (Meridian Life Sciences, Memphis, USA) in compliance with cGMP and all batches reviewed and approved by virologists. The final product will be supplied as a separate aliquot for each subject.

11.2 Supply and Accountability

RVL will ensure that all deliveries and returns of Challenge Virus are received and documented by a responsible person. Accurate accountability records detailing receipt, condition and dispensing of all Challenge Virus inoculum stock will be maintained. Documentation will be available for verification by the Study Monitor, QA, or Quality Control (QC) staff and other parties as appropriate.

11.3 Storage

Inoculum will be stored at ≤-70 ºC in a secure freezer; vials will be thawed just prior to inoculation. Once thawed, and if not used within the time acceptable (as per short term stability study results) vials of inoculum virus will not be re-used for human studies.

All used and unused inoculum virus vials should be maintained by RVL.

The residual inoculum in the virus vials used for challenging the subjects will be snap frozen and stored in accordance with RVL SOPs and the RVL-vCS-003 AP.

Accountability records for storage will be maintained and remain available for verification by the Study Monitor, QA, or QC staff and other parties as appropriate.

11.4 Preparation and Administration

The A/Perth/16/2009 (H3N2) inoculum will be prepared by an approved sub-contractor, checked by the lead virologist and the Translational Study Lead (TSL), released to the CI (or deputies) as per relevant SOPs and then administered intranasally in drops by pipette in accordance with RVL's SOPs. Inoculum will be supplied in frozen, pre-diluted aliquots.

11.5 Disposal

Any unused Challenge Virus inoculum vials that are no longer required may be disposed of according to RVL's SOPs for the destruction of bio-hazardous waste. Unused Challenge Virus will be retained in accordance with RVL's SOPs.

11.6 Unused Challenge Virus

The CI will ensure that unused Challenge Virus is retained until verification and accountability procedures have been performed. Challenge Virus that is no longer required may be stored by RVL.

12. RANDOMISATION AND BLINDING

12.1 Randomisation

12.1.1 Cohort 1

This is a double blind randomised study. Subjects of cohort 1 will be randomly allocated to a Challenge Virus titre via a randomisation code list which will be computer generated using a balanced permuted block algorithm between subjects for up to four groups.

12.1.2 Cohort 2

Subjects of cohort 2 will receive only 1 Challenge Virus titre; 1 of the 4 used in cohort 1. Therefore, no randomisation procedure will be necessary.

12.2 Blinding (Cohort 1)

A limited number of staff designated to prepare the inoculum dilution(s) will be unblinded in order to apply the randomisation code provided by the third party statistical contractor on the day of Viral Challenge.

Neither the subjects nor those dispensing the inoculum will know which inoculum dilution a subject has been randomised to receive.

Subjects will be assigned a RVL subject number that will be used to identify them up to the point of Viral Challenge. A randomisation number will also be assigned to each subject to be inoculated, just before the Viral Challenge.

12.3 Unblinding

For the first stage of the study, where necessary (e.g. in a medical emergency), code breaking and unblinding of a subject's randomisation will be performed as per RVL SOPs.

The second stage of the study is an open-label arm with cohort 2 receiving only 1 titre of Challenge Virus. Therefore, as part of the interim analysis, the unblinding will take place prior to stage 2.

13. STUDY PROCEDURES

All procedures will be undertaken in compliance with GCP and in accordance with RVL SOPs.

13.1 Time and Events Schedule

Details of the timing of study assessments and procedures can be found in
Table 13-1: Time and Events Schedule. Where appropriate, additional safety procedures may be performed at the Study Physician's discretion.

Table 13-1: Time and Events Schedule

Assessments	Screening Phase				Quarantine Phase											Follow-Up Phase
	Pre-Screening Visit*	Study-Specific Visit	Admission to Quarantine		Viral Challenge/ Inoculation			Post-Viral Challenge Study Procedures and Assessments							Discharge	End of Study Visit
	Days	Days	Day	Day	Day 0			Day	Day	Day	Day	Day	Day	Day	Day	Day
	Day -80 to Day -3	Day -56 to Day -3	-2	-1	Pre challenge	Challenge	Post challenge	1	2	3	4	5	6	7	8	28 ± 5d
	Pre-Screening Visit*	Study-Specific Visit														
Written informed consent	X	X														
Eligibility criteria		X	X	X^{a}	X											
Demographics		X														
Medical history and prior medications	X**	X	X	X^{a}												
Height[c], Weight, BMI and waist measurement		X	X^{e}	X^{ea}												X
Randomisation					X											
Challenge Virus Inoculation						X										
Anti viral medication (Tamiflu-(oseltamivir))[h]														BD^{h}	X^{h}	
Complete Physical Examination		X	X	X^{a}											X	X
Directed Physical Examination		X	X	X^{a}	X		X	X	X	X	X	X	X	X	X	X
Vital signs (BP, RR, HR and SpO_2)		X	X^{b}	3 x (every 8 hrs)[f]	3 x (every 8 hrs)[fk]			3 x (every 8 hrs)[fk]							X	X
Tympanic temperature		X	X^{b}	X^{b}	3 x (every 8 hrs)[fk]			3 x (every 8 hrs)[fk]							X	X
RVL and Wisconsin Symptom Diary Cards			X	3 x (every 8 hrs)[f]	3 x (every 8 hrs)[fk]			3 x (every 8 hrs)[fk]							X	
12-lead ECG		X	X	X^{a}						X				X		X
Spirometry		X	X	X^{a}	X			X^{e}	X^{e}	X^{e}	X^{e}	X^{e}	X^{e}	X^{e}	X^{e}	X
24h Tissue count and mucus weight				X^{g}	X			X	X	X	X	X	X	X	X	
Adverse events and concomitant medications		Continuous														

(X)- At Study Physician's discretion, if indicated; BD- Twice daily; TDS- Three times daily; *Pre-screening visit applies only to volunteers aged 46 to 64 years (age cohort 2), essential only if there is no serum sample on RVL records; ** Brief medical history questionnaire for age cohort 2 at the pre-screening visit
a- if not performed on Day -2; b- up to 3 x a day; c-Height at Screening only; d-for tolerance only at screening; e-weight on Day -2/Day -1 will be used as reference for spirometry throughout Quarantine; f-Symptom diary cards, vital signs and temperature recordings to be taken at same time points on each day; g-initial distribution of tissues on Day-1 for baseline; h- For subjects with continuing virus infection, antiviral medication will be given for 5 days, as per product licence; j - samples taken at the same time as the nasopharyngeal swabs; k- window for assessments is ± 30 minutes.

Time and Events Schedule (continued)

Assessments	Screening Phase		Quarantine Phase														Follow-Up Phase
	Pre-Screening Visit*	Study-Specific Visit	Admission to Quarantine		Viral Challenge/ Inoculation			Post-Viral Challenge Study Procedures and Assessments							Discharge		End of Study Visit
	Days	Days	Day	Day	Day 0			Day	Day	Day	Day	Day	Day	Day	Day		Day
	Day -80 to Day -3	Day -56 to Day -3	-2	-1	Pre challenge	Challenge	Post challenge	1	2	3	4	5	6	7	8		28 ± 5d
Cannulation for blood draws			X	X^a													
Blood- Challenge Virus Serology	X	X	X	X^a													X
Blood- Haematology		X	X	X^a						X				X			X
Blood- Coagulation		X	X	X^a													X
Blood- Clinical chemistry (including cardiac enzymes)		X	X	X^a						X				X			X
Blood- C-reactive protein		X	X	X^a						X				X			X
Blood- Thyroid function test		X	X	X^a													X
Blood- Blood type	X																
Blood- Hepatitis A, B & C and HIV serology		X															
Blood- Pregnancy test β-HCG (females)			X	X^a													
Blood-Sera (cytokines/ immune markers/proteomics)				X^a	X					X				X			
Blood- PBMC				X	X					X				X			
Blood - PAXgene RNA				X	X					X				X			
Blood - PAXgene DNA				X													X
Nasopharyngeal swab (respiratory viral screen -either RVP/DFA/PCR)			X	X^a													
Nasopharyngeal swab ($TCID_{50}$/ qPCR)		X^d		X				3 x (every 8 hrs)[k]							X		X
Nasopharyngeal swab (Rapid diagnostic test)														X			
Breath alcohol test			X	X^a											X		
Urine screen for drugs of abuse and cotinine		X	X	X^a													
Urinalysis (dipstick)		X	X	X^a						X				X			X
Urine pregnancy test (females)		X			X												X

(X)- At Study Physician's discretion, if indicated; BD- Twice daily; TDS- Three times daily; *Pre-screening visit applies only to volunteers aged 46 to 64 years (age cohort 2), essential only if there is no serum sample on RVL records; ** Brief medical history questionnaire for age cohort 2 at the pre-screening visit
a- if not performed on Day -2; b- up to 3 x a day; c-Height at Screening only; d-for tolerance only at screening; e-weight on Day -2/Day -1 will be used as reference for spirometry throughout Quarantine; f-Symptom diary cards, vital signs and temperature recordings to be taken at same time points on each day; g-initial distribution of tissues on Day-1 for baseline; h- For subjects with continuing virus infection, antiviral medication will be given for 5 days, as per product licence; j- samples taken at the same time as the nasopharyngeal swabs; k- window for assessments is ± 30 minutes.

13.2 Screening Phase

13.2.1 Pre-Screening Visit (from Day -80) (Cohort 2 only)

A Pre-Screening Visit (from Day – 80) will be performed to assess initial serosuitability prior to a Study-Specific Screening Visit. Serosuitable subjects of this cohort will then attend the SSS visit for assessment of their eligibility to participate in the study.

The following procedures will be performed during this visit:

- Brief medical history
- Informed consent
- Blood sample (10mL) for:
    - Challenge Virus serology (serosuitability)
    - Blood type
    - Peripheral Blood Mononuclear Cells

13.2.2 Study Specific Screening (Day -56 to Day -3) (All Cohorts)

Volunteers who are confirmed sero-suitable (likely to become infected with the A/Perth/16/2009(H3N2) will be invited to attend a SSS visit, when the following procedures will be performed in a clinical environment:

- Written informed consent (Section 20.7.2.)
- Eligibility criteria
- Demographics
- Medical history and prior medications
- Height, weight, BMI and waist measurement
- Complete physical examination
- Directed physical examination
- Vital signs: Blood pressure (BP), respiratory rate (RR), heart rate (HR), oxygen saturation (SpO_2), tympanic temperature
- 12 - lead ECG
- Spirometry
- AEs and concomitant medications
- Bloods
    - Challenge Virus serology (if there is not a valid result within the serology time period)
    - Haematology
    - Coagulation
    - Clinical Chemistry (including cardiac enzymes, TFT and C-reactive protein (CRP)
    - HIV, Hepatitis A, B and C serology
- Nasopharyngeal swab (tolerance only)
- Urine screen for Class A drugs and cotinine
- Urinalysis (dipstick)

- Urine pregnancy test (females only)

Volunteers found to be ineligible at any point during the screening phase will be excluded from further screening assessments.

Eligible subjects will be invited to attend for the Quarantine and HVC phase of the study.

13.3 Admission to Quarantine (Day -2/ Day -1)

Subjects will attend the Quarantine Unit at the beginning of the Quarantine phase.
The following procedures will be undertaken and the results will be reviewed with respect to the eligibility criteria.

13.3.1 Day -2 OR Day -1

On admission to Quarantine on Day -2 (or on Day -1 if not done on Day -2):

- Eligibility criteria
- Medical history and prior medications
- Weight, BMI and waist measurement
- Complete physical examination
- Directed physical examination
- Vital signs: BP, RR, HR, SpO2, (up to three times a day on Day -2)
- Tympanic temperature (up to three times a day)
- RVL and Wisconsin Symptom Diary Cards
- 12 - lead ECG
- Spirometry
- AEs and concomitant medications
- Bloods
  - Challenge Virus serology
  - Haematology
  - Coagulation
  - Clinical Chemistry (including cardiac enzymes and CRP)
  - Blood pregnancy test - β-Human Chorionic Gonadotropin (β-HCG)
- Blood
    - Sera
    - PBMC
    - PAXgene RNA
    - PAXgene DNA
- Nasopharyngeal swab (Respiratory Virus Panel (RVP)/DFA/PCR)
- Breath alcohol test
- Urine screen for Class A drugs and cotinine
- Urinalysis

13.3.2 Day -1

- Vital signs: BP, RR, HR and SpO_2 (every 8 hours)

- RVL and Wisconsin Symptom Diary Cards (every 8 hours)
- 24 hour tissue collection commences (initial supply given in the morning)
- AEs and concomitant medications
- Nasopharyngeal swab ($TCID_{50}$ and qPCR)

Depending on the time of a subject's arrival in Quarantine on Day -1, the required interval between some assessments (e.g., eight hourly) may not be possible to achieve. Therefore, for subjects admitted on Day -1, apart from admission assessments, the scheduling of other Day -1 assessments will aim to meet the required frequency for those assessments and not the required interval.

Eligible subjects will remain resident in the Quarantine Unit. Subjects who no longer meet the study eligibility criteria, have evidence of a respiratory infection, or the CI judges they are unfit to be inoculated with the Challenge Virus, will be discharged and replaced with reserve subjects.

Subjects withdrawn from the study will be asked to complete an End of Study Follow up visit.

During the Quarantine period, additional safety tests (e.g., urine screen for Class A drugs and cotinine, breath alcohol, spirometry, ECG, blood safety tests) may be undertaken at the Study Physician's discretion, in order to ensure the subjects' on-going safety and compliance with the study restrictions.

13.4 Viral Challenge (Day 0)

On the morning of Day 0, subjects who have been confirmed as eligible for the study will be assigned a randomisation code by the RVL laboratory. The CI and clinical team will remain blinded to each subject's virus titre allocation.

The following procedures will be performed throughout Day 0:

- Vital signs - BP, RR, HR, SpO2 (every 8 hours)
- Tympanic temperature (every 8 hours)
- RVL and Wisconsin Symptom Diary Cards (every 8 hours)
- AEs and concomitant medications

The following procedures will also be performed:

13.4.1 Pre- Viral Challenge

- Eligibility criteria
- Randomisation
- Directed physical examination
- Spirometry
- 24 hour tissue collection
- Urine pregnancy test (females)

13.4.2 Viral Challenge

- Inoculation with Challenge Virus A/Perth/16/2009(H3N2)

13.4.3 Post- Viral Challenge

- Directed physical examination

13.5 Quarantine Days 1 to 7

Procedures and assessments during the post-Viral Challenge Quarantine period (Days 1 to 7) are shown in
Table 13-1: Time and Events Schedule. Assessments that are scheduled as 2 or 3 times daily will all be undertaken at approximately the same timepoints as per required timelines.

13.5.1 Daily

- Directed physical examination – (morning)
- Vital signs - BP, RR, HR, SpO_2 – (every 8 hours)
- Tympanic temperature - (every 8 hours)
- RVL and Wisconsin Symptom Diary Cards - (every 8 hours)
- Spirometry
- 24 hour tissue collection
- AEs and concomitant medications
- Nasopharyngeal swab ($TCID_{50}$ and qPCR) - (every 8 hours)

13.5.2 Day 3

- 12-lead ECG
- Blood
  - Haematology
  - Clinical chemistry including cardiac enzymes and CRP
  - Sera
  - PBMC
  - PAXgene RNA
- Urinalysis

13.5.3 Day 7

Start twice-daily treatment with anti-viral medication (e.g., oseltamivir [Tamiflu®]) to continue for 5 days for subjects with evidence of continuing viral infection.

- 12-lead ECG
- Blood
  - Haematology
  - Clinical chemistry including cardiac enzymes and CRP
  - Sera
  - PBMC
  - PAXgene RNA

- Nasopharyngeal swab (rapid diagnostic test (Section 17.2.2))
- Urinalysis

13.6 Discharge from Quarantine (Day 8)

Subjects who are well enough for discharge will leave the Quarantine Unit on the morning of Day 8. Otherwise, subjects may be required to stay in Quarantine for one or more extra night(s). All decisions relating to the subject's suitability for discharge into the community will be at the CI's discretion.

13.6.1 Prior to discharge (Day 8)

- Morning dose of anti-viral medication
- Complete physical examination
- Directed physical examination
- Vital signs: BP, RR, HR and SpO_2
- Tympanic temperature
- RVL and Wisconsin Symptom Diary Cards
- Spirometry
- Complete 24 hour tissue collection
- AEs and concomitant medications
- Nasopharyngeal swab ($TCID_{50}$ and qPCR)
- Breath alcohol test.

Subjects will be given instructions to contact the CI in the event of a medical emergency after discharge from Quarantine.

13.6.2 Days 9-11

Subjects will be advised to self-administer twice-daily doses of anti-viral medication (e.g., oseltamivir [Tamiflu®]) in order to complete the prescribed dose regimen as per the product licence.

13.7 Withdrawal Visit

If a subject withdraws from Quarantine, every effort will be made to complete all the required withdrawal visit assessments. The details and reason for the withdrawal, if known, will be recorded in the source documents.

For withdrawn subjects, the Day 28 (± 5 days) Follow up safety assessments may be conducted at the CI's discretion.

13.8 Follow up Visit

13.8.1 Day 28 (± 5 days)

This End of Study Follow up Visit marks the completion of the subjects' planned participation in the study. The following procedures and assessments will be performed:

- Weight, BMI and waist measurement
- Complete physical examination
- Directed physical examination
- Vital signs: BP, RR, HR and SpO_2
- Tympanic temperature
- 12 - lead ECG
- Spirometry
- AEs and concomitant medications
- Nasopharyngeal swab ($TCID_{50}$ and qPCR)
- Blood
  - Challenge Virus serology
  - Haematology
  - Coagulation
  - Clinical Chemistry (including cardiac enzymes, CRP and TFT)
  - PAxgene DNA
- Urinalysis
- Urine pregnancy test (females only).

13.9 Subject Discontinuation (Lost to Follow Up)

If a subject does not return to the clinic for their final visit, the CI should make every effort to contact the subject to review all AEs.

14. DEFINITIONS OF ILLNESS AND INFECTION

14.1 Infection

14.1.1 Laboratory-Confirmed Influenza Infection

- A positive cell culture assay at least once during Quarantine post-Viral Challenge
  - And/or if analysed by qPCR: At least two positive detections by any qPCR assay, (if a single sample is used, it must be confirmed positive by analysis of a separate aliquot) between virus inoculation and the day of discharge from Quarantine.
- And/or: Seroconversion

14.1.1.1 Challenge Virus Seroconversion

From a pre Viral Challenge baseline to the highest follow up titre:

- Greater than or equal to a (≥) 4 fold increase in antibodies to Challenge Virus.

14.1.1.2 Seroprotection

Seroprotection is an antibody titre of 40 or greater.

14.1.2 Viral Replication

- A positive cell culture assay at least once during Quarantine post Viral Challenge.

14.1.3 Viral Shedding

- A positive cell culture assay at least once during Quarantine post Viral Challenge
- And/or if analysed by qPCR:
  - At least two positive detections by any qPCR assay, (if a single sample is used, it must be confirmed positive by analysis of a separate aliquot) between Day 1 post-Viral Challenge and the day of discharge from Quarantine.

14.2 Illness

14.2.1 ILI (Influenza-like Illness)

Defined by any incidence of either:

- FI
- LRTI
- URTI
- SI

14.2.2 Laboratory confirmed ILI

- ILI (any incidence of either URI or LRI or SI or FI)
- AND
- Laboratory confirmed infection.

14.2.3 Febrile Illness

If the subject has, post Viral Challenge:

- any occurrence of a temperature of ≥37.9°C (confirmed by a repeat measurement as ≥37.9°C within 20 to 60 minutes)

OR

- Normal values - two standard deviations greater than a baseline measure.

14.2.4 Lower Respiratory Tract Illness

A subject will be considered to have LRTI if he/she has any one of the following on 22 consecutive days, at least one day of which must attain Grade 2 severity, or if any of the following attain Grade 3 severity once:

- Self-reported symptoms:
  - Cough
  - Shortness of Breath

- Physician findings:
  - Abnormal breath sounds (new wheezing, râles or rhonchi)

Or

  - Other lower respiratory tract signs.

14.2.5 Upper Respiratory Tract Illness

A subject will be considered to have an URTI if he/she has any one of the following symptoms on two consecutive days, at least one day of which must attain Grade 2 severity, or if any of the following attain Grade 3 severity on one or more days:

- Self-reported symptoms:
  - Nasal congestion (stuffy nose)
  - Rhinorrhoea (runny nose)
  - Sneezing
  - Sore throat

- Physician findings:
  - Otitis
  - Nasal discharge
  - Sinus tenderness

14.2.6 Systemic Illness

A subject will be considered to have systemic illness (SI) if he/she has any one of the following on two consecutive days, at least one day of which must attain Grade 2 severity, or if any of the following attain Grade 3 severity once:

- Chilliness/fever
- General malaise
- Headache persisting for >1 hour

- Rhonchi
- Myalgia and/or arthralgia.

14.2.7 Sub-clinical Infection

The criteria for sub-clinical infection are met if the subject:

- does not fulfil ILI
  and
- does fulfil laboratory confirmed evidence of infection.

14.2.8 Non-sick and uninfected

The criteria for 'non-sick and uninfected' are met if the subject:

- does not fulfil ILI, and
- does not fulfil laboratory confirmed evidence of infection.

14.3 Seroconversion and Seroprotection

Seroconversion is a fourfold increase in antibody titre; sero-protection is a titre of 40 or greater.

15. CLINICAL ASSESSMENTS

At scheduled times throughout the study period (see
Table 13-1: Time and Events Schedule) the following clinical assessments will be performed according to RVL standard procedures, and the results recorded in the source documentation. Whenever possible, the same observer will perform assessments of individual subjects. The time window for assessments specified as 4- or 8-hourly will be ± 30 minutes.

Where appropriate, the normal ranges for clinical assessments and the study timepoints used to determine baseline values are detailed in the relevant RVL SOP/ Operating Instruction (OI).

15.1 Medical History

Medical history will be elicited from each volunteer during SSS, and will include a complete review of body systems, past medical and surgical histories, and any allergies.

Based on the information obtained, subjects will be assessed for their eligibility to participate in the study according to the inclusion and exclusion criteria.

15.2 Prior and Concomitant Medications

Medications taken up to the time of SSS will be documented at the Study-Specific Screening Visit. Thereafter, review of any concomitant medication will continue until the End of Study Follow up Visit; all new and changed medications will be recorded in the source documents.

15.3 Height, Weight, BMI and Waist Measurement

Height (m), body weight (kg), calculated BMI [kg/m^2], and waist measurement (to calculate body fat percentage) will be recorded at SSS and on Day -2/-1.

Body weight, BMI, and waist measurement will also be calculated at the end of study Follow up Visit.

BMI will be calculated as BMI [kg/m^2] = Body weight [kg] ÷ Height^2 [m^2]

15.4 Physical Examination

Any clinically significant changes in the physical examination that are not assessed as symptoms of A/Perth/16/2009(H3N2) infection during the study will be recorded as an AE and dealt with according to RVL SOPs. The CI may perform additional physical examination assessments to evaluate or manage clinical illness.

15.4.1 Complete Physical Examination

Complete physical examination includes:

- Examination of the:
    - Cardiovascular system
    - Respiratory system
    - Musculoskeletal system

  - Neurological system
  - Abdominal examination
  - Lymph nodes
  - Skin

- and other general examination as judged necessary by the CI

15.4.2 Directed Physical Examination

Any clinically significant changes not assessed as symptoms of A/Perth/16/2009(H3N2) infection will be considered to be AE(s) and will be dealt with according to RVL SOPs (Section 18).

The directed physical examination includes

- Examination of the:
  - Ear
  - Nose
  - Throat
  - Chest (via stethoscope)

- Daily (morning) assessment of any upper and lower respiratory symptoms (nasal discharge, otitis, pharyngitis, sinus tenderness, new wheezes, râles and rhonchi) graded according to the appropriate standard procedures.

Physician-reported assessments of URTI resulting from Challenge Virus infection will be graded in accordance with their intensity as absent, mild, moderate or severe and will include:

- Nasal discharge
- Otitis
- Sinus tenderness
- Pharyngitis

15.5 Vital Signs

Vital signs (BP, RR, HR and SpO_2) will be recorded after the subject has been resting for at least five minutes as follows:

- BP (Systolic and Diastolic) - Where possible the same arm will be used for each recording.
- Respirations will be counted for 15 seconds and recorded in breaths per minute
- Heart rate in beats per minute (bpm)
- % SpO_2 will be recorded using a pulse oximeter placed over the subject's finger or ear

A severity grading will be assigned to any vital signs that are out of the normal range. Clinically relevant abnormal vital signs will be reported as AEs and dealt with accordingly.

The Common Terminology Criteria for Adverse Events (CTCAE)[24] and/or Division of AIDS Table For Grading the Severity of Adult and Paediatric Adverse Events Guidance (DAIDS)[25] and/or the Division of Microbiology and Infectious Diseases (DMID) Adult Toxicity Table may be used as guidance for grading of AE severity.

Additional vital sign or physical examination assessments may be performed in order to evaluate or manage clinical illness.

15.6 Temperature

Tympanic temperature will be recorded at the times shown in
Table 13-1. Tympanic temperature recordings taken on Days -2 through to discharge will be evaluated for virulence and pathogenicity of the A/Perth/16/2009(H3N2) infection.

Temperature is critical to definitions of illness and to symptomatic and specific therapy criteria, therefore temperatures of ≥ 37.9ºC must be confirmed by a repeat measurement not less than 20 minutes and not more than 60 minutes after the first reading. If confirmed by a second reading, the first temperature measurement will be used (but both readings will be recorded).

15.7 12-Lead Electrocardiogram

12-lead Electrocardiogram (ECG) will be read on site by an appropriately qualified Investigator. Wherever possible the same Investigator will review subsequent ECGs from the same subject for the assessment of any change from baseline.

The following data will be calculated at the time of recording:

- HR (normal 50-100 beats per minute (bpm))
- PR interval (normal 0.12 to 0.2 sec)
- QRS duration (normal < 0.12 sec)
- QT interval (normal < 0.33-0.44 sec)
- QTc interval (normal- male <0.43 sec, female < 0.45 sec).

A central reader may also be used in all cases to review and interpret the ECG; however the investigator/PI will be responsible for the initial interpretation of all ECGs.

Any clinically significant changes in the ECG (from SSS) will be recorded as an AE.
The CI will assess non-clinically significant changes to determine whether they should be recorded as AEs.

15.8 Spirometry

Up to eight blows will be taken to obtain three technically acceptable measurements at each time-point; the best reading from the assessment will be used for analysis.

The following will be calculated:

- FEV_1 (absolute)
- FEV_1 (% predicted)
- FVC (absolute)
- FVC (% predicted)
- FEV_1/FVC (absolute)
- FEV_1/FVC (% predicted)
- Maximum Mid-Expiratory Flow (MMEF) (absolute)
- MMEF (% predicted).

Any clinically significant change from baseline in pulmonary function during the study will be recorded as an AE.

Any drop in spirometry pre-inoculation will not be due to the Challenge Virus so will not be considered to be an AE unless other factors make it clinically significant.

In comparison to baseline values, a 15% drop in subsequent spirometry values will be considered a Grade 1 AE (mild). Above this, the CI will use clinical judgement to assign severity.

15.9 Alcohol Breath Test

Subjects will breathe into a breath Alco-meter to check for evidence of alcohol consumption.

15.10 Tissue Count and Mucus Weight

All paper tissues used by each subject will be collected for each 24-hour period throughout the Quarantine period, to determine number of tissues used and the weight of mucus expelled.

Paper tissue and bag distribution will start on Day -1 at 0800 (± 1h).

Subjects will be given pre-weighed packets of paper tissues. After a subject has used a tissue for nose blowing or sneezing into, the subject will place them into separate bags for collection the following morning.

Collection of used tissues will start on Day 0 at approximately 0800 (± 1h) and will be performed daily until Day 8 or until the subject is finally discharged.

15.11 Symptom Diary Card

Throughout the Quarantine period, subjects will be asked to record their symptoms on two symptom diary cards, the RVL Symptom Diary Card (Appendix 1), and the Wisconsin Symptom Diary Card (Appendix 2) (Appendix 2).

The quarantine applicable questions from the Wisconsin Symptom diary card will be considered for this study.

Definitions of illness resulting from A/Perth/16/2009(H3N2) Challenge Virus infection are described in Section 14.

The Symptom Diary Cards and clinician interviews will be used to elicit and record subjects' symptoms.

Symptoms rated greater than Grade 0 will be presumed to represent A/Perth/16/2009(H3N2) infection consequent to challenge, and will not be additionally captured as AEs (Section 18) unless they meet one of the criteria for a SAE, or at the discretion of the CI (Section 18.1.2).

The Study Physician will review the subject's symptom diary cards on a daily basis after admission to Quarantine. Any AEs will be recorded in the source documents.

15.12 Adverse Events

The Study Physician is responsible for the detection and documentation of events meeting the criteria for AEs or SAEs (Section 18.2) from the time of completion of written informed consent until completion of the Follow up Visit.

Throughout the study, from Screening to the Follow up Visit, subjects will be asked to report any unusual, undesirable, or unwanted symptoms, worsening of existing conditions or changes in their health and wellbeing. Subjects will be asked to report any problems and the Study Physician will periodically enquire about the occurrence of AEs and need for concomitant medications.

During Quarantine, subjects will be probed about AEs twice a day using open-ended questions, for example:

- How are you feeling?
- Have you had any medical problems since your last visit/assessment?
- Have you taken any new medicines, other than those given to you in this study, since your last visit/assessment?

Adverse events will be assessed and recorded as described in Section 18.

16. LABORATORY ASSESSMENTS

Laboratory safety values that are outside the reference range will be evaluated by the CI using CTCAE, DMID and/or DAIDS grading. The Study Physician will sign the laboratory report and clinically relevant values will be managed as described in Section 18.

Laboratory normal ranges will be provided by the contract laboratory.

16.1 Blood

Blood samples will be collected and analysed in accordance with RVL SOPs, at the study timepoints detailed in
Table 13-1: Time and Events Schedule. The amount of blood that will be drawn in the study will not exceed 473 mL.

Samples will be drawn for the following analyses:

16.1.1 <u>Clinical Safety</u>

If required, additional safety samples may be taken at the discretion of the CI.

Samples will be analysed for the following parameters:

16.1.1.1 Haematology

- Platelet Count
- White blood cell (WBC) count differential
- Red blood cell (RBC) count
- Neutrophils
- WBC (absolute)
- Lymphocytes
- Reticulocyte count
- Monocytes
- Haemoglobin
- Eosinophil's
- Haematocrit
- Basophils
- Mean corpuscular volume (MCV)
- Mean corpuscular haemoglobin (MCH)
- Mean corpuscular haemoglobin concentration (MCHC)

16.1.1.2 Clinical Chemistry

- Total and direct bilirubin
- Fasting Glucose (only if clinically indicated at CI's discretion)
- Lactate dehydrogenase (LDH)
- Sodium
- Gamma Glutamyl Transferase (GGT)

- Potassium
- Albumin
- Chloride
- Alkaline phosphatase (ALP)
- Bicarbonate
- Aspartate aminotransferase (AST)
- Calcium
- Alanine aminotransferase (ALT)
- Urea
- TFT
- Uric Acid
- Total Protein
- Creatinine
- C-reactive protein (CRP) (see Section 18.1.3)

16.1.1.3 Coagulation

Blood samples for coagulation will be collected for the following analyses:

- Prothrombin time (PT)
- Activated partial thromboplastin time (APTT)
- International normalised ratio (INR)
- APTT Ratio

16.1.1.4 Cardiac Enzymes

Blood samples for cardiac enzymes will be collected for analysis of:
- Troponin I/T

16.1.1.5 HIV, Hepatitis A, B and C

Blood samples for HIV, HAV, HBV and HCV antibodies will be collected at SSS.

16.1.1.6 Pregnancy Test

A blood serum β-HCG pregnancy test will be performed on entry to the Quarantine Unit on Day -2/ Day -1.

16.2 Urine

At various timepoints during the study (see
Table 13-1: Time and Events Schedule), urine samples will be collected and analysed in accordance with RVL SOPs. Additional testing may be undertaken at the CI's discretion.

16.2.1 Urinalysis

Urinalysis will be performed using dipsticks, which detect the following:
- pH
- ketones

- protein
- nitrite
- bilirubin
- urobilinogen
- blood
- leukocytes
- glucose
- specific gravity

16.2.2 Class A Drugs and Cotinine Screen

Urine samples will be tested for evidence of Class A drugs of abuse and cotinine (nicotine) at the study timepoints detailed in
Table 13-1: Time and Events Schedule, and at the CI's discretion. The test will be performed using the RVL standard test kit.

16.2.3 Pregnancy test

All females will have a urine pregnancy test at the study timepoints detailed in
Table 13-1: Time and Events Schedule. Urine pregnancy tests may also be performed at any time during the study at the CI's discretion.

17. PATHOGENICITY AND VIRULENCE SAMPLES AND ANALYSIS

During the study (see
Table 13-1: Time and Events Schedule), the following samples will be collected and analysed for viral disease in accordance with RVL SOPs.

Providing appropriate consent is in place, additional samples may be collected for RVL exploratory purposes.

The time window for all samples specified as eight hourly will be ± 30 minutes.

17.1 Blood

17.1.1 Sera for Challenge Virus Serology

Blood samples will be taken to determine A/Perth/16/2009(H3N2) specific antibody responses at Pre-Screening Visit (where applicable) and SSS Visit.

To be eligible for the study a subject must be 'sero-suitable' i.e. have low immunity to the Challenge Virus, which is defined by low levels of HAI antibodies to A/Perth/16/2009(H3N2).

Each subject will be initially screened for pre-existing A/Perth/16/2009(H3N2) specific antibody responses using an HAI assay.

Additional immunological assays may be used to further investigate immunity to Influenza, to assist in understanding the virulence and pathogenicity of the Challenge Virus.

17.1.2 Additional Sera

Sera will be analysed for proteomics and inflammatory markers to investigate the human-virus interaction.
Sera will also be collected and used for discovery purposes to investigate the human-virus interaction and to better understand influenza disease and the correlates of protection in relation to biomarkers.

17.1.3 PAXgene (RNA & DNA)

Blood will be collected into PAXgene tubes for RNA analysis in order to investigate the human transcriptomic profiles when exposed to challenge virus and to better understand the pathogenicity and virulence of Influenza disease.

Blood will also be collected into PAXgene tubes for DNA analysis in order to investigate the human genomic profiles to understand susceptibility to Influenza infection and disease.

17.1.4 PBMC

Blood will be collected for PBMC separation. PBMCs will be used in various assays to investigate the proteomic, genomic and transcriptomic profiles when exposed to challenge virus and to better understand the pathogenicity and virulence of Influenza disease.

PBMCs will be used for discovery purposes to investigate the human-virus interaction and to better understand influenza disease and the correlates of protection:

- proteomics and biomarkers
- transcriptomics
- genomic sequencing (if appropriate DNA consent is provided)
- exploratory purposes, to investigate the human-virus interaction and to better understand influenza disease and the correlates of protection.

17.2 Nasopharyngeal Swab

A nasopharyngeal swab will be performed at SSS to determine the subject's tolerance of the procedure.

During the study, nasopharyngeal swabs will also be taken for:

- RVP /DFA and/or PCR
- Rapid diagnostic test (Section 17.2.2)
- Cell culture (e.g., $TCID_{50}$)
- qPCR, and
- exploratory purposes to investigate the human-virus interaction and to better understand influenza disease and the correlates of protection.

17.2.1 Respiratory Virus Panel

A respiratory virus panel will be performed using a nasopharyngeal swab sample, to test for the presence of other respiratory viruses, which could potentially contraindicate a volunteer's participation in the study.

17.2.2 Rapid diagnostic test

A rapid diagnostic test will be used to determine the presence of the Challenge Virus in a nasopharyngeal swab sample taken on Day 7. The following assays may be utilised for this purpose:

- Rapid Viral Antigen Test (RVAT) and/or the
- Rapid Time PCR (RT-PCR) test

18. ADVERSE EVENTS

18.1 Definitions

18.1.1 Adverse Event

An AE is any untoward medical occurrence in a subject, regardless of its relationship to the product being studied/ administered.

An AE can be any unfavourable and unintended sign (including an abnormal laboratory finding), symptom, or disease temporally associated with the use of the Challenge Virus, whether or not considered related to the Challenge Virus.

An AE includes:

- Exacerbation of a pre-existing illness
- Increase in frequency or severity of a pre-existing episodic condition
- A condition detected or diagnosed after Challenge Virus administration even though it may have been present prior to the start of the study
- Complications and termination of a pregnancy

An AE does not include:

- Medical or surgical procedures (e.g., surgery, endoscopy, tooth extraction, transfusion); the condition that leads to the procedure is an AE
- Pre-existing disease or conditions present or detected prior to start of Challenge Virus administration that do not worsen
- Hospitalisation for elective surgery, social and/or convenience admissions provided they are arranged before the start of Challenge Virus administration
- Over- administration of either virus inoculum or concomitant medication without any signs or symptoms
- An uncomplicated pregnancy or an induced elective abortion to terminate a pregnancy without medical reason
- Typical/normal viral symptoms on diary cards

18.1.2 Serious Adverse Events

A SAE is an untoward medical occurrence that:

- Results in death
- Is life-threatening
- Requires inpatient hospitalisation or prolongation of existing hospitalisation
- Results in persistent or significant disability/incapacity
- Is a congenital anomaly/birth defect

The term life threatening refers to an event in which the patient was, in the opinion of the CI or delegate, at risk of death at the time of the event. It does not refer to an event that hypothetically might have caused death if it were more severe.

Medical and scientific judgment should be exercised in deciding whether expedited reporting is appropriate in other situations, such as important medical events that may not be immediately life threatening or result in death, but may jeopardize the patient or may require intervention to prevent one of the outcomes listed in this definition above. These should also usually be considered serious.

Examples of such events are intensive treatment in an acute care environment or at home for allergic bronchospasm, blood abnormalities or convulsions that do not result in inpatient hospitalisation, or the development of drug dependency or drug abuse.

18.1.3 Clinically Significant Laboratory Abnormalities and Other Abnormal Assessments

Laboratory results and other safety assessments will be reviewed by a Study Physician. Values outside the reference range will be documented as AEs if they meet the CTCAE/DAID/DMID criteria (Appendix 3). In some cases, significant changes within the normal range may also need to be assessed by the CI.

The CI will monitor all clinically significant abnormal laboratory results or assessments until they return to normal or baseline values (i.e. resolve), stabilise, or are no longer clinically significant. Where appropriate, (e.g., after a period of monitoring of an abnormal result), clinically significant abnormalities should be reassessed by the CI to determine whether an AE has changed.

Abnormalities will be rated either as clinically significant (CS) or as not clinically significant (NCS).

Influenza-associated laboratory abnormalities (e.g., elevated ALT, AST or GGT; decreased neutrophils) may be recorded as AEs although reported in the source data as NCS.

The severity of the laboratory abnormalities will be determined based on the grading scales specified in the protocol (NCI CTCAE/DAIDS/DMID or 2007 Food and Drug Administration (FDA) Guidance).

The following will be used as grading criteria for C-reactive protein (CRP) values: Any value above 5 mg/L but less than 60 mg/L will be considered as Grade 1 AE (mild). Clinical judgement will be used to assign the grading above Grade 1 and in all assessments will be based on evaluation of clinical signs and symptoms.

All clinically significant laboratory abnormalities deemed to be AEs or SAEs will be documented in the source documents. Relationship to the administration of Challenge Virus will be assessed.

The following will be used as grading criteria for spirometry values: In comparison to baseline values, a 15% drop in subsequent spirometry values will be considered a Grade 1 AE (mild). Above and beyond this, the CI will use clinical judgement to assign severity.

All AEs will be captured on the AE log regardless of clinical significance.

18.2 Recording Adverse Events

All AEs, with the exception of spirometry (see Section 15.8), will be recorded from the SSS baseline visit until the Day 28 (± 5 days) Follow up Visit, or until the resolution of the AE. AEs will be fully recorded in the source documents as they are reported, whether spontaneously volunteered by a subject or in response to questioning about wellbeing at each study visit. Enquiries about AEs should cover the period between the previous and current visit.

The CI will review all documentation (e.g., laboratory, or diagnostic reports) relative to the event and record all relevant information in the source documents.

AEs will be assessed as follows:

18.2.1 Description

If the event consists of a cluster of signs and symptoms, a diagnosis should be recorded (e.g., gastroenteritis) rather than each sign and symptom.

18.2.1.1 Onset and End

The dates and times of the onset and end of the event should be recorded.

18.2.1.2 Seriousness

The CI must record whether the AE meets the definition of serious. If the event is serious, the CI must complete a SAE Report Form (see Section 18.1.2).

18.2.1.3 Intensity and Severity

Intensity is defined as one of the following:

- Mild: awareness of sign or symptom, but easily tolerated
- Moderate: discomfort sufficient to cause interference with normal activities
- Severe: incapacitating, with inability to perform normal activities

It is important to distinguish between serious and severe AEs.

Severity is a measure of intensity whereas seriousness is defined by the criteria provided in Section 18.1.2. An AE of severe intensity need not necessarily be considered serious.

For example, a migraine headache that incapacitates a subject for many hours may be severe, whereas a stroke that results in a limited degree of disability may be considered mild, but should be reported as a SAE.

18.2.1.4 Causality

Every effort should be made by the CI to assess whether the AE has any relationship to the Challenge Virus, any study procedure or assessment, concomitant medication or other treatment.
Causality will be classified using the following categories:

Related-	There is an association between the event and the administration of Challenge Virus Inoculum, and a plausible mechanism for the event to be related to the Challenge Virus Inoculum. Causes other than the Challenge Virus Inoculum have been ruled out, and/or the event re-appeared on re-exposure to the Challenge Virus Inoculum.
Possibly Related-	There is an association between the event and the administration of the Challenge Virus Inoculum and there is a plausible mechanism for the event to be related to Challenge Virus Inoculum, but there may also be alternative aetiology, such as characteristics of the subject's clinical status or underlying disease.
Unlikely Related-	The event is unlikely to be related to the Challenge Virus Inoculum and likely to be related to factors other than Challenge Virus Inoculum.
Not Related-	The event is related to aetiology other than the Challenge Virus Inoculum (the alternative aetiology must be documented in the study subject's medical record).

18.2.1.5 Action Taken

Actions taken should be recorded as either:

- None
- AE required treatment

18.2.1.6 Outcome

An AE should be followed until the CI has determined and provided the outcome, or an alternative explanation has been provided. The outcome should be classified according to the categories shown below:

Recovered/ Resolved	Resolution of an AE with no residual signs or symptoms
Recovered/ Resolved with sequelae	Resolution of an AE with residual signs or symptoms
Not recovered/ not resolved (continuing)	Either incomplete improvement or no improvement of an AE, such that it remains on-going
Fatal	Outcome of an AE is death. "Fatal" should be used when death is at least possibly related to the AE.
Unknown	Outcome of an AE is not known (e.g., a subject lost to follow up).

18.2.1.7 Follow up

All AEs and SAEs must be followed-up by the CI or referred to the subject's GP for follow up until they return to normal or baseline values (are resolved) or stabilise, or until they are judged by the CI to be no longer clinically significant.

Supplemental measurements and/or evaluations may be necessary to investigate the nature and/or causality of an AE or SAE. This may include additional laboratory tests, diagnostic procedures, or consultation with other healthcare professionals. If the subject dies, any post-mortem findings (including histopathology) must be provided to RVL.

18.2.2 Pregnancy

Pregnancy of a study subject or subject's partner during or within 3 months of inoculation is not a SAE but must still be reported to the CI.

Congenital anomalies/birth defects are SAEs, therefore pregnancies occurring during the study must be recorded to allow subject follow up to birth. Pregnancy related information must be reported as per the RVL SOP.

If appropriate, the pregnant individual may be referred to their GP or to a specialist. The CI will be responsible for informing the REC as appropriate.

18.3 Reporting SAEs

All SAEs should be reported immediately to the CI and SMEs. The immediate and follow up reports will be completed promptly as per RVL SOPs.

These SAE reports will identify subjects by a unique code number assigned to the subject rather than by the subject's names, personal identification numbers and or addresses.

There are no SAEs identified that would not require immediate reporting.

Grade 3 to 4 laboratory abnormalities must be reported to the CI, who will determine whether immediate reporting to the appropriate SME is required.

As per RVL SOPs, the CI in collaboration with the SME should promptly notify the REC of all findings that:

- could adversely affect the safety of subjects
- impact the conduct of the trial
- alter the REC favourable opinion to continue
- are fatal or life/threatening

Table 18-1: SAE Reporting Requirements

	**INITIAL REPORTS**		**FOLLOW UP REPORTS**
**Type of SAE**	**Fatal or Life-threatening SAE**	**Other SAEs**	**Any SAE**
**REPORTING REQUIREMENTS**	Within 24 hours<br>Telephone notification<br><br>Within 48 hours<br>Fully completed SAE Report Form to SME	Within 48 hours<br>Fully completed SAE Report Form to SME	Within 48 hours<br>Updated SAE Report Form to SME

**Reporting SAEs to the SMEs**	
**Name**	Dr Ganesh Balaratnam
**Name**	Dr Martin Johnson
**SAE Mobile Number**	0845 330 5664
**SAE e-mail address**	RVLSAEreporting@retroscreen.com

18.3.1 Research Ethics Committee

The CI must notify the REC of any SAEs or unexpected AEs regardless of their relationship to the Challenge Virus. Concurrently, the CI must send documentation of notification of the REC to the SMEs.

The REC will be sent safety updates at least once a year in order to facilitate their continuing review of the study as required.

18.4 Post-Study Obligations

The CI is not obliged to seek AEs or SAEs in former study participants, but should notify the SMEs if he/she becomes aware of any SAE or death of a study subject following the subject's participation in the study, and such event(s) is (are) reasonably related to the HVC.

Pregnancies should be managed as described in Section 18.2.2.

19. STATISTICAL METHODS AND PLANNED ANALYSES

S-cubed will perform statistical analysis for the study on behalf of RVL.

An interim analysis will be performed on cohort 1 before deciding which Challenge Virus titre will be used for subjects in cohort 2.

19.1 Study Populations

In all reporting presentations where the virus titre is shown, subjects will be grouped and presented against the titre of challenge virus that the subject actually received.

19.1.1 Safety Analysis Set

The Safety population is defined as all randomised subjects receiving the A/Perth/16/2009(H3N2) Challenge Virus inoculum. Unless otherwise indicated, all baseline and safety analyses will be performed on the Safety analysis set. Data for any subjects who were randomised but did not receive challenge virus inoculum will be presented in subject listings only.

19.1.2 Virulence and Pathogenicity Analysis Set

The Virulence and Pathogenicity analysis set is defined as all randomised sero-suitable subjects receiving the A/Perth/16/2009(H3N2) Challenge Virus.

The Virulence and Pathogenicity analysis set will be considered the primary analysis population for virulence and pathogenicity analyses. All Virulence and Pathogenicity analyses will be performed on this analysis set.

19.1.3 Per Protocol (PP) Analysis Set

The PP population is defined as all virulence and pathogenicity analysis set subjects who have no major protocol deviations and who complete the Quarantine period up to the final day of Quarantine, Day 8 (Discharge).

The PP analysis set will be considered the secondary analysis population for virulence and pathogenicity analyses. All virulence and pathogenicity analyses will be performed on this analysis set, if it is different from the Virulence and Pathogenicity analysis set.

19.2 Data Management

RVL will be responsible for the supervision of the overall conduct of data management for this study, using qualified individuals throughout all stages in accordance with the principles of GCP:

- Trial process from design
- Electronic case report forms (eCRF) data handling
- Data verification
- Planning and conducting the statistical analyses (in conjunction with S-cubed)
- Preparing the final report

Prior to starting data processing, a Data Management Plan (DMP) will be produced, which will describe and define all data management activities, and the database structure and contents.

Data will only be collected from subjects that have signed a written informed consent form (ICF) and in accordance with the DMP via the eCRF provided by SureSource™. RVL will ensure in conjunction with the eCRF provider this electronic data processing system conforms to the established requirements for

- Completeness
- Accuracy
- Reliability
- Consistency with intended performance (i.e. validation)
- Maintain SOPs for use
- Ensure the system is designed to permit data changes to maintain an audit trail, data trail and edit trail
- Maintain a security system the prevents unauthorised access to the data
- Maintain a list of the individual authorised to make data changes
- Safeguard the blinding
- It will be possible to compare the original data and observations if the data is processed
- An unambiguous subject identification code that allows identification of the all the data reported will be used.

Prior to database lock the database will undergo quality control as detailed in the DMP. A 100% check will be carried out on critical variables. Acceptance rates for critical variables will be 0% error, and 0.25% for all other data. Following database lock, the final database will be transferred as SAS™ datasets to the Study Statistician for statistical analysis and reporting as per the agreed SAP.

The study site will comply with UK Data privacy regulations. All source documents must be kept in order and up-to-date so that they reflect the latest observations on the enrolled subjects. All entries, corrections, and alterations must be made by the CI or other authorised study-site personnel. The CI must verify that all data entries in the source documents are accurate and correct.

Source data will be collected either in paper form or directly in the eCRF (SureSource™) as documented in the Source Document agreement.

During the study, data will be made available for review by the CI and statistics team via pre-defined reports extracted from the database at agreed intervals documented in the DMP.

19.3 Data Coding

AEs and concomitant medications will be coded as documented in the DMP.

19.4 Sample Size

No formal sample size calculation has been performed for this exploratory phase study.

Subjects will be recruited in 2 cohorts; cohort 1 is comprised of subjects 18 – 45 years and cohort 2 will be comprised of subjects between 18 – 64 years inclusive.

In cohort 1, a total of up to 28 subjects will be studied in up to 4 virus titre groups, each with up to 8 subjects per group. In cohort 2 up to 28 subjects will receive 1 titre of Challenge Virus.

19.5 Interim Analysis

Prior to cohort 2, analysis and unblinding of cohort 1 will be undertaken to identify the optimum Challenge Virus titre for use in cohort 2. Results from cohort 1 will be kept securely and will be limited to a pre-defined selection of staff.

19.6 Randomisation (Cohort 1)

Subjects will be randomly allocated to one of up to four groups of up to eight subjects per group (see Table 8-1) via a randomisation code list, which will be computer generated using a permuted block algorithm in a 1:1:1:1 ratio. Each group will be administered one of up to four different inoculum titres (see Section 8.2).

19.7 Statistical Analysis Plan

Data will be analysed and reported using SAS® version 9.2 or later.

All endpoints in this section, unless otherwise specified, will be analysed and presented separately for both cohorts 1 and 2.

Due to the sample size in this study, no statistical comparison of groups is planned. The study will be summarised in terms of descriptive statistics. Continuous variables will be summarised using number of observations, mean, standard deviation, median, minimum and maximum values. Categorical variables will be summarised using proportions (counts and percentages).

Summary tables will be presented by virus titre and for all subjects.

Accounting for the occurrence of and extent of missing data, and its possible impact on the study analysis will be described within the SAP.

A detailed SAP will be developed, and approved by RVL prior to any lock of the study database. The SAP will give a more detailed description of the report presentations to be produced, expanding on the protocol specified analysis. Any deviation from the protocol specified analysis will be documented within a protocol or SAP amendment, as appropriate, and described within the Clinical Summary Report (CSR).

Further post-hoc evaluations of exploratory endpoints may be performed and these will be separately reported and identified in the CSR.

19.7.1 Subject Accountability

The number of subjects randomised, inoculated, withdrawing (also split by reason for withdrawal) from and completing the study, and the numbers in each analysis set, will be summarised for all subjects and by virus titre.

19.7.2 Protocol Deviations

Subject data will be reviewed for major protocol deviations prior to database lock at a planned data review meeting, and decisions will be documented within the meeting minutes. At this meeting, the analysis populations will also be reviewed and finalised.

19.7.3 Subgroup Analysis

A subgroup identifying laboratory-confirmed infected subjects will be used for reporting (in addition to the analysis sets). This subgroup is defined as those subjects who are laboratory-confirmed (by viral shedding and/or seroconversion, (see Section 14.1) infection.

19.7.4 Demographic and Baseline Characteristics

Descriptive statistics of demographics (age, sex, height, weight, BMI, body fat at Screening, and ethnicity) will be presented by virus titre and overall across all subjects. Medical history information will be listed.

19.7.5 Primary Efficacy Analysis

The primary endpoint is the AUC of the Challenge Viral load, measured by nasopharyngeal swab qPCR, post Viral Challenge to the last day of Quarantine.

The qPCR values will be used to calculate the AUC for each subject using the trapezium rule. The AUC will be summarised by virus titre and overall across all subjects. The mean qPCR values will be displayed graphically by day and virus titre.

19.7.6 Secondary Efficacy Analysis

Secondary efficacy analyses will be performed on the following endpoints:

19.7.6.1 Viral Load AUC and Daily AUC

Similar presentations will be produced as for the viral load AUC by nasopharyngeal swab qPCR endpoint.

- The AUC of the Challenge Viral load, measured by nasopharyngeal swab cell culture, post Viral Challenge to the last day of Quarantine.
- The daily AUC of the Challenge Viral load, measured by nasopharyngeal swab qPCR, post Viral Challenge to the last day of Quarantine.
- The daily AUC of the Challenge Viral load, measured by nasopharyngeal swab cell culture, post Viral Challenge to the last day of Quarantine.

19.7.6.2 Peak value, delta peak value, time to peak, duration, time to resolution from peak, and daily incidence of the Challenge Viral load, measured by nasopharyngeal swab qPCR, after Viral Challenge

These endpoints will only be reported for subjects who are deemed to be “virus shedders”, i.e. those subjects who specifically satisfy the qPCR related part of the definition of viral shedding (not applying the viral culture part of the definition) given in Section 14.1.3 apart from daily incidence (which will be reported for all subjects), and these endpoints are based on the time period post inoculum to the last day of Quarantine.

Peak value:
Using the scheduled protocol assessments from post inoculum to the last day of Quarantine, this endpoint corresponds to the highest observed virus titre value.

Delta Peak value:
Using the scheduled protocol assessments from post inoculum to the last day of Quarantine, this endpoint corresponds to the difference between the viral load on day of peak (for each subject) above the viral load on the first detection of virus shedding (for that subject).

Time to Peak:
Using the scheduled protocol assessments from post inoculum to the last day of Quarantine, this endpoint corresponds to the number of days from post inoculum until the peak is observed. If peak is seen on more than one day, then take first occurrence.

Duration:
Using the scheduled protocol assessments from post inoculum to the last day of Quarantine, this endpoint corresponds to the time (in days) from the day where virus shedding (see Section 14.1.3) was first observed to the day where virus shedding was last observed. This does not depend on virus shedding being observed on days within these period boundaries.

Time to resolution from peak:
Using the scheduled protocol assessments from post inoculum to the last day of Quarantine, this endpoint corresponds to the time (in days) from the day when the peak value was observed until the last day that virus shedding is observed.

Daily incidence:
Using the scheduled protocol assessments from post inoculum to the last day of Quarantine, this endpoint corresponds to the proportion of subjects achieving virus shedding (see Section 14.1.3) on at least one assessment, separately considered for each day, post inoculum to the last day of quarantine.

The mean incidence values will be displayed graphically by assessment on each day and virus titre.

19.7.6.3 The peak value, delta peak value, time to peak, duration, time to resolution from peak, and daily incidence of the Challenge Viral load, measured by nasopharyngeal swab cell culture, after Viral Challenge

These endpoints will only be reported for subjects who are deemed to be "virus shedders", i.e. those subjects who specifically satisfy the cell culture related part of the definition of viral shedding (not applying the qPCR part of the definition) given in Section 14.1.3, apart from daily incidence (which will be reported for all subjects), and these endpoints are based on the time period post inoculum to the last day of Quarantine. The definitions for peak value, delta peak value, time to peak, duration, time to resolution from peak and daily incidence are as per previously defined for nasopharyngeal swab qPCR analyses.

19.7.6.4 The peak value, delta peak value, time to peak, duration, time to resolution from peak, and daily incidence of the Challenge Viral load, measured by nasopharyngeal swab qPCR, after Viral Challenge.

19.7.6.5 Proportion of subjects with laboratory-confirmed influenza infection

Laboratory-confirmed Influenza Infection is defined in Section 14.2.2. The proportion of subjects with Laboratory-confirmed Influenza infection will be summarised by virus titre and overall across all subjects.

19.7.6.6 Proportion of subjects with viral shedding

Viral shedding is defined in Section 14.1.3. The proportion of subjects with viral shedding will be summarised by virus titre and overall across all subjects.

19.7.6.7 Proportion of subjects with viral replication

Viral replication is defined in Section 14.1.2. The proportion of subjects with viral replication will be summarised by virus titre and overall across all subjects.

19.7.6.8 AUC and daily AUC of total symptom score, from po*st inoculum to the last day of* Quarantine.

Total symptom scores (i.e. sum of all 10 symptom scores for each assessment) will be used to calculate the AUC from post inoculum to the last day of Quarantine, for each subject using the trapezium rule. The following types of symptoms are recorded (on a grading scale, 0 to 3):

- URT symptoms: runny nose, stuffy nose, sneezing, sore throat, earache
- LRT symptoms: cough, shortness of breath
- Systemic symptoms: headache, malaise (tiredness), muscle and/or joint ache

The total symptom scores will be summarised by virus titre and also across all subjects. The AUC will be summarised by virus titre and overall across all subjects. The mean total symptom scores will be displayed graphically by day and virus titre.

19.7.6.9 AUC and daily AUC of component symptom scores, from post inoculum to the last day of *Q*uarantine

The components correspond to:

- URT symptoms
- LRT symptoms
- Systemic Respiratory Tract (SRT) symptoms

AUC presentations (calculated in a similar way to total symptoms scores) for each component will be produced.

19.7.6.10 AUC and daily AUC of total symptom score using individual symptom scores Grade 2 or higher, from post inoculum to the last day of Quarantine

An AUC presentation (calculated in a similar way to total symptoms scores) for total symptoms scores, using only symptom scores Grade 2 or higher, will be produced.

19.7.6.11 AUC and daily AUC of component symptom scores using individual symptom scores Grade 2 or higher, from po*st inoculum to the last day of* Quarantine

AUC presentations (calculated in a similar way to component symptoms scores) for component symptoms scores, using only symptom scores Grade 2 or higher, will be produced.

19.7.6.12 The proportion (on any occasion, and on two separate occasions), duration, peak value, time to peak, time to resolution and daily incidence, of any symptoms Grade 2 or higher, after Viral Challenge

Symptoms are recorded on the symptom diary cards. The data will be presented as follows:

Proportion from post inoculum to the last day of quarantine:
The proportion of subjects who develop any of the 10 individual symptoms Grade 2 or higher (separately on any occasion and on two separate occasions) from post inoculum to the last day of Quarantine will be calculated. The proportion of subjects with symptoms will be summarised by virus titre and overall across all subjects.

Duration:
Using the scheduled protocol scheduled assessments from post inoculum to the last day of quarantine, this endpoint corresponds to the time (in days) from the first occurrence of any Grade 2 or higher symptom to the day when a Grade 2 or higher symptom was last observed.

Peak value:
Using the scheduled protocol assessments from post inoculum to the last day of Quarantine, this endpoint corresponds to the highest observed symptom score (defined as the day where there are most Grade 2 or higher symptom values observed).

Time to Peak:
Using the scheduled protocol assessments from post inoculum to the last day of Quarantine, this endpoint corresponds to the number of days post inoculum until the peak is observed. The peak is defined as the day when the most Grade 2 or higher symptom values are observed. If peak occurs on more than one day then the first occurrence is selected.

Time to Resolution:
Using the scheduled protocol assessments from post inoculum to the last day of Quarantine, this endpoint corresponds to the time to resolution of symptoms from day of peak symptoms to resolution.

Daily Incidence:
This endpoint is similar to the above proportion endpoint in definition, except incidence is separately considered for each day.

19.7.6.13The proportion (on any occasion, and on two separate occasions), duration, peak value, time to peak, time to resolution, and daily incidence, of the URT and LRT components of symptoms grade 2 or higher after Viral Challenge

The components correspond to:

- URT symptoms
- LRT symptoms

Similar presentations as for all symptoms scores will be produced, except each (URT and LRT symptoms) component will be separately considered.

19.7.6.14The proportion (on any occasion, and on two separate occasions), duration, peak value, time to peak, time to resolution and daily incidence, of symptoms of any grade after Viral Challenge

Similar presentations as for the endpoint based on Grade 2 or higher symptoms will be produced, but any grade symptoms will be considered.

19.7.6.15The proportion (on any occasion, and on two separate occasions), duration, peak value, time to peak, time to resolution and daily incidence, of the URT and LRT components of symptoms of any grade after Viral Challenge

Similar presentations as for the endpoint based on Grade 2 or higher component symptoms will be produced, but any grade component symptoms will be considered.

19.7.6.16The proportion, AUC, peak value, duration and time to resolution of febrile illness after Viral Challenge

Febrile illness is defined in Section 14.2.3. The proportion of subjects who develop febrile illness will be calculated. A single occurrence of febrile illness anytime from post inoculum to the last day of Quarantine corresponds to meeting the endpoint. The proportion of subjects with febrile illness will be summarised by virus titre and overall across all subjects.

Duration:
Duration is defined as the time from initially achieving or exceeding 37.9 ^{o}C to the timepoint after the last time that the temperature is equal to or above 37.9 ^{o}C.

Time to Resolution:

Time to resolution is defined as time from peak temperature to the timepoint after the last time that the temperature is equal to or above 37.9 °C.

In addition, summary statistics for AUC, peak value, absolute tympanic temperature values, and changes from baseline will be tabulated by virus titre and all subjects.

19.7.6.17 The proportion of subjects with influenza-like illness (ILI), laboratory confirmed ILI, Upper Respiratory Tract Illness (URTI), Lower Respiratory Tract Illness (LRTI), Systemic Illness (SI), Non-sick but infected subjects (subclinical infection), Non-sick and uninfected, after Viral Challenge

Definitions for each endpoint are shown in the following sections:

- ILI, Section 14.2.1
- Laboratory confirmed ILI, Section 14.2.2
- URTI, Section 14.2.5
- LRTI, Section 14.2.4
- SI, Section 14.2.6
- Non-sick but infected subjects (sub-clinical infection), Section 14.2.7
- Non-sick and uninfected, Section 14.2.8

The proportion of subjects with each of the above definitions will be summarised by virus titre and overall across all subjects.

19.7.6.18 Proportion of subjects with seroconversion to Viral Challenge

Seroconversion is defined in Section 14.1.2. The incidence of seroconversion will be separately summarised by virus titre and across all subjects.

19.7.6.19 Proportion of subjects with seroprotection to Viral Challenge

Seroprotection is defined in Section 14.3. The incidence of seroprotection will be separately summarised by virus titre and across all subjects.

19.7.6.20 Mucus weight assessed by paper tissue weights and tissue count

Mucus weight (in grams) will be calculated from tissue weights. Mucus weight will be summarised by virus titre (and across all subjects) separately for all scheduled visits from post inoculum to the last day of Quarantine. In addition, the mucus weight values will be used to calculate the AUC. The AUC will be summarised by virus titre and overall across all subjects.

In addition, total weight of mucus divided by sum of symptoms scores post inoculum to the last day of Quarantine will be calculated and summarised by virus titre and overall across all subjects.

The mean mucus weight values will be displayed graphically by day and virus titre.

In addition, the number of tissues used will be displayed graphically by day and virus titre.

<u>Exploratory Analyses</u>

Exploratory endpoints are described in Section 7.5.1. Where relevant, exploratory endpoints will be summarised by virus titre and across all subjects.

19.7.7 Safety Analysis

19.7.7.1 Adverse Events (AEs)

The safety endpoint of the study is the incidence of virus challenge emergent AEs that are not consistent with a mild to moderate influenza infection. These AEs will be summarised by virus titre.

In addition, the incidence of all virus challenge emergent AEs will be reported within summary presentations, by Medical Dictionary for Regulatory Activities (MedDRA) system organ class (SOC) and preferred term, by virus titre. An AE will be classified as virus challenge emergent if the onset date of the AE is on or after the date of inoculation (Study Day 0). Should any onset date for an AE be missing or only a partial date recorded (such that it cannot be determined if the event onset was prior to virus inoculation or not) then it will be assumed that the event is virus challenge emergent, unless the AE stop date indicates otherwise. Any AE with an onset date earlier than the date of inoculation will be classified as a pre-inoculation AE.

If a subject experiences more than one AE with the same preferred term, that preferred term will be counted only once in summary presentations. It will be assigned the worst observed severity and the strongest relationship to the Challenge Virus among those events for summaries in which those characteristics are considered.

Summary presentations will be produced for the number and percentage of subjects reporting virus-emergent: AEs, severity of AEs and AEs related to Challenge Virus. In addition, SAEs and AEs directly resulting in withdrawal from study will be listed.

19.7.7.2 12-Lead ECG

Summary statistics for changes from baseline will be tabulated, by virus titre and all subjects, for ECG parameters (HR, PR, QRS, QT and QTc) and will be included within subject listings.

19.7.7.3 Laboratory Parameters

Summary statistics for maximum changes from baseline will be tabulated, by virus titre and all subjects, for absolute laboratory parameters (i.e. haematology, clinical biochemistry, coagulation, cardiac enzymes, blood glucose, thyroid function test and urinalysis). A scatter plot for each laboratory parameter will be produced over time, identifying subjects from the virus titres. Laboratory values outside the normal range will be identified in subject listings.

19.7.7.4 Vital Signs

Summary statistics for changes from baseline will be tabulated, by virus titre and all subjects, for vital signs parameters (systolic blood pressure (SBP), diastolic blood pressure (DBP), respiratory rate (RR), heart rate (HR), SpO_2 and temperature).

19.7.7.5 Physical Examination

Physical examination findings (complete and directed examinations) will be included within subject listings.

19.7.7.6 Spirometry

Summary statistics for changes from baseline will be tabulated, by virus titre and all subjects, for spirometry parameters (FEV1 % predicted (%), FEV1/FVC ratio (absolute), FEF 25-75%/MMEF (%), FEV1 [base] (absolute), FVC [base] (absolute), FVC % (%), FEV1/FVC ratio % (%), FEF 25-75%/MMEF [base] (L/sec)) and will also be included in the subject listings. For all spirometry parameters, a scatter plot will be produced over time, identifying subjects from the virus titres.

19.7.7.7 Concurrent Medications

Medications will be assigned as being prior to or concomitant with Challenge Virus (i.e. given prior to or after receiving Challenge Virus) based on the start and stop dates of the medication and the timing of receiving the Challenge Virus. If the medication stop date is before the date of virus inoculation, the medication will be assigned as being prior to Challenge Virus. In all other situations, the medication will be assigned as being concomitant with the Challenge Virus. Prior and concomitant medications (separately identified) will be included in subject listings.

19.7.8 Combined Cohort Analysis

An analysis combining subjects of both cohorts will be performed. The endpoints for this combined analysis will be similar to those specified in Section 19.7 of the protocol, and will compare data across age ranges.

In addition a regression analysis comparing age will be performed for the following efficacy and safety endpoints:

- Virus shedding
- Symptoms
- AEs
- SAEs

Further details of this combined (cohort) analysis will be specified in the SAP.

20. STUDY MANAGEMENT AND RESPONSIBILITIES

All ethical and legal requirements must be met before the first subject is enrolled in the study.

20.1 Ethics Review

The protocol and ICF will be reviewed and approved by a REC.

20.2 Deviations from the Protocol and Protocol Amendments

20.2.1 Protocol Deviations

Any unplanned or unintended departures from the study protocol will be documented on a protocol deviation log as soon as possible after they occur, by the CI or other appropriately qualified person.

20.2.2 Serious Breach

Any serious breach of the conditions and principles of GCP or the Trial Protocol in connection with the study will be reported to the CI and the REC. A serious breach is defined as any breach that is likely to affect to a significant degree either:

- The safety or physical or mental integrity of the subjects
- The scientific value of the trial

20.2.3 Protocol Amendments

Once the study has started, protocol amendments should only be made in exceptional cases. The CI will decide whether an amendment meets the definition of substantial or non-substantial, and must approve all amendments before their implementation.

A substantial amendment is an amendment that is likely to have a significant impact on the:

- Safety or physical or mental integrity of the subjects
- Scientific value of the trial
- Conduct or management of the trial
- Quality or safety of the Challenge Virus used in this study

Amendments that have an impact on subject risk or the clinical trial objectives, or require revision of the informed consent document, must receive Sponsor and REC approval prior to their implementation.

20.3 Study Discontinuation

RVL reserves the right to temporarily suspend or discontinue the study for any reason. In addition, the study may be stopped at any time if, in the opinion of the SME, safety data suggest that the medical safety of subjects is being compromised.

RVL reserves the right to terminate the study for refusal of the CI to supply source documentation of work performed in this clinical study.

If the study is suspended or terminated, the CI will promptly inform the REC with the reason(s) for the action. All study data must then be returned to RVL. The site must also conduct final disposition of all unused protocol mandated non-IMP in accordance with RVL SOPs.

20.4 Study Records Retention and Direct Access to Source Documents

The CI shall keep a copy of the paper and electronic source documents and the Study Master File (SMF). Data will be reviewed and signed by the CI or designee as applicable.

The CI shall allow inspections of the study site and any source documentation by clinical research and audit personnel, external auditors or representatives of regulatory authorities/ethics committees, and will allow direct access to source documents.

Direct access to the subject’s paper or electronic medical/clinical records (if applicable to the study) is necessary to verify and corroborate the data recorded on the source documents (Source Document Verification (SDV)). The anonymity of the subject will be respected during the review of these documents. The CI shall keep the paper and electronic source documents as per RVL SOPs.

20.5 Sponsor Responsibilities

It is the Sponsor’s responsibility to obtain the appropriate approvals to perform the study, and to report to regulatory authorities the results of this study as appropriate.

20.6 Monitoring and Auditing

The Sponsor will describe the appropriate extent and nature of the monitoring in a Monitoring Plan (MP). Monitoring is performed to verify that:

- The rights and wellbeing of the subjects are protected
- The reported trial data are accurate, complete and verifiable from source
- The conduct of the study complies with the approved protocol and GCP

The CI will allow the Study Monitors direct access to all relevant documents, and allocate his time and staff to the Monitor to discuss and resolve findings or relevant issues.

20.7 Investigator Responsibilities

The CI shall ensure that all persons assisting with the study are adequately informed about the protocol and any amendments, their study-related duties and functions. The CI should maintain a list of sub-investigators or delegates and other appropriately qualified persons who have been delegated significant study-related duties. The CI may delegate his/her responsibilities to suitably qualified staff provided that their details and delegated roles and responsibilities are described in the study delegation log.

20.7.1 Ethical Considerations

The CI shall make all required REC submissions and notifications/updates and obtain all required approvals in accordance with RVL SOPs.

Study progress reports will be submitted to the REC by the CI in accordance with local regulatory practices and in agreement with the Sponsor.

The REC shall be informed about the end of the trial with the required timelines:

- Routine termination- 90 days from the protocol-defined end of trial date or event
- Early termination- 15 days from trial completion

20.7.2 Informed Consent

Potential subjects will be provided with a copy of the Volunteer Information Sheet (VIS) and Informed Consent Form (ICF) to read and consider prior to the Pre-Screening and Study- Specific Screening visits.

Prior to entry to the study, during the informed consent procedure, the CI or designated physician will explain to each volunteer, in lay language, the study procedures and any known or likely risks associated with the study; any questions will be answered to the volunteer's satisfaction.

A properly executed, written, ICF, in compliance with the Declaration of Helsinki, ICH GCP, and other applicable regulations will be obtained from volunteers who are willing to participate.

The ICF must be signed and dated by the subject and countersigned by the CI or designated physician (whoever conducted the consent discussion).

Two copies of the Volunteer Information Sheet (VIS) and ICF will be made.

- The original will be held in the SMF with the Screening and Enrolment Logs
- A copy will be given to the subject
- A copy will be retained in the subject's study notes

Subjects will be assured that:

- they can withdraw from the study at any time and for any reason without prejudice to their medical care
- they will be informed in a timely manner if new information becomes available that may affect their willingness to continue participation in the study.

The communication of this information shall be documented.

Specific informed consent will be required for the use of samples for DNA analysis.

If the ICF is amended during the study, the CI shall follow all applicable regulatory requirements pertaining to approval of the amended ICF by the REC. The site must use the amended ICF for all new subjects and repeat the consent process with the amended ICF for any on-going subjects if required.

20.8 Laboratory Certification and Normal Values

The CI will inform the Sponsor of the name and location of the clinical laboratory(s) used for laboratory tests, and retain a copy of certification for all laboratory tests included in the protocol, (including certification number and date of certification), and a list of the normal values for all laboratory tests required by the protocol. Updated versions must be provided as appropriate.

These documents must be available before any subject is treated in the study.

20.9 Information for the Subject's General Practitioner

The CI will ensure that the subject's GP is informed by letter of the subject's participation in the study.

20.10 Compensation and Expenses

RVL will reimburse subjects for their inconvenience and out-of-pocket expenses, including travelling costs. The REC shall approve all proposed payments to subjects prior to the start of the study and the Volunteer Information Sheet will detail the payment schedule.

20.11 Non-Protocol Research

No investigational research procedures pertaining to this study other than those outlined in this protocol may be undertaken on the subjects or their biological samples without the prior written permission of the subject, RVL, the REC and, when appropriate, the national regulatory authority.

20.12 Liability and Insurance

RVL subscribes to an insurance policy for insurance/indemnity in its terms and provisions, its legal liability as a Sponsor and a CI site for injuries caused to participating persons and arising out of this research performed strictly in accordance with the scientific protocol as well as with applicable law and professional standards.

20.13 Audits and Inspections

To ensure the study is conducted and the study data are generated, documented and reported in compliance with the protocol, GCP and regulatory requirements, RVL will implement and maintain quality assurance (QA) and quality control (QC) systems as detailed in the Study Audit Plan and relevant SOPs. The CI will ensure direct access to all trial related sites source documents and reports for the purpose of monitoring and auditing.

If a regulatory agency conducts an inspection of the study, the CI will allow the Inspector direct access to all source documents and other study documentation for source data checking and/or on-site audit inspection, and allocate his time and the time of his staff to the Inspector to discuss findings of any relevant issues.

20.14 Study Termination

Upon completion of the study, the Study Monitor and CI will ensure the following activities are completed as appropriate:

- Provision of all study data to RVL

- Data clarifications and/or resolutions
- Review of site study records for completeness

RVL reserves the right to suspend or prematurely terminate this study for any reason (see Section 20.3).

21. DISCLOSURE OF DATA

21.1 Subject Confidentiality

The CI shall reassure subjects that their confidentiality will be maintained during all audits and inspections of the study site and documentation by third parties. A unique study number will be assigned to subjects at the start of the study, and will be used to identify the subject on study documentation, study correspondence and in the study database. The CI will keep an identification code list and enrolment log that will list the full name of each subject alongside the subject number assigned and the date enrolled. This log will remain in the SMF at the study site.

21.2 Sponsor Confidentiality

RVL will use confidential information solely for conducting this study.

21.3 Publication Policy

All unpublished information provided and held by RVL is confidential and will remain the sole property of RVL. We intend to publish the results of this study as soon as practical following completion and we are committed to the open dissemination of research results. Where appropriate, RVL prefers publication in open access journals to ensure maximum access for others in the scientific and medical communities. In addition we will do our utmost to ensure that volunteers involved in our studies are kept informed of any publications that arise from studies they have been involved in, their privacy will be protected at all times.

21.4 Study Documentation

21.4.1 Source Documents

The CI shall keep the SMFs and source documents until notified otherwise by RVL.

22. REFERENCES

1. *ICH Harmonised Tripartite Guideline for Good Clinical Practice E6(R1) (ICH GCP).* 1996.
2. *Declaration of Helsinki*. 1996, World Medical Association: Republic of South Africa.
3. Jones, S., et al., *DNA vaccination protects against an influenza challenge in a double-blind randomised placebo-controlled phase 1b clinical trial.* Vaccine, 2009. **27**(18): p. 2506-12.
4. DeVincenzo, J., et al., *A randomized, double-blind, placebo-controlled study of an RNAi-based therapy directed against respiratory syncytial virus.* Proc Natl Acad Sci U S A, 2010. **107**(19): p. 8800-5.
5. Zaas, A.K., et al., *Gene expression signatures diagnose influenza and other symptomatic respiratory viral infections in humans.* Cell Host Microbe, 2009. **6**(3): p. 207-17.
6. Lillie, P.J., et al., *Preliminary assessment of the efficacy of a T-cell-based influenza vaccine, MVA-NP+M1, in humans.* Clin Infect Dis, 2012. **55**(1): p. 19-25.
7. Woods, C.W., et al., *A host transcriptional signature for presymptomatic detection of infection in humans exposed to influenza H1N1 or H3N2.* PLoS One, 2013. **8**(1): p. e52198.
8. WHO. *Influenza*. 2012 18 July 2012; Available from: http://www.who.int/biologicals/vaccines/influenza/en/index.html#.
9. *Oseltamivir, amantadine (review) and zanamivir for the prophylaxis of influenza;TA158* N.I.f.H.a.C.E.N. http://publications.nice.org.uk/oseltamivir-amantadine-review-and-zanamivir-for-the-prophylaxis-of-influenza-ta158/clinical-need-and-practice, Editor.
10. Greaves, K., et al., *The prevalence of myocarditis and skeletal muscle injury during acute viral infection in adults: measurement of cardiac troponins I and T in 152 patients with acute influenza infection.* Arch Intern Med, 2003. **163**(2): p. 165-8.
11. CDC. *Seasonal Influenza*. 2013; Available from: http://www.cdc.gov/flu/about/viruses/types.htm.
12. GlaxoSmithKline, *Relenza® (zanamivir)* in *Package Insert*.
13. Genentech USA, I., *Tamiflu® (oseltamivir phosphate)*, in *Package Insert*.
14. Forest Pharmaceuticals, I., *Flumadine® (rimantadine hydrochloride)*, in *Package insert*.
15. EndoPharmaceuticals, *Symmetrel® (amantadine hydrochloride)* in *Package insert*.
16. WHO. *Influenza*. 2013; Available from: http://www.who.int/biologicals/vaccines/influenza.
17. Killingley, B., et al., *Use of a human influenza challenge model to assess person-to-person transmission: proof-of-concept study.* J Infect Dis, 2012. **205**(1): p. 35-43.
18. Longini, I.M., et al., *Estimation of the efficacy of live, attenuated influenza vaccine from a two-year, multi-center vaccine trial: implications for influenza epidemic control.* Vaccine, 2000. **18**(18): p. 1902-9.
19. Wilkinson TM, L.C., Chui CS, Huang AK, Perkins M, et al, *Preexisting influenza-specific CD4+ T cells correlate with disease protection against influenza challenge in humans.* Nat Med, 2012. **18**: p. 274-280.
20. Macintyre CR1, H.A., Kovoor P, Ridda I, Seale H, Tan T, Gao Z, Katelaris AL, Siu HW, Lo V, Lindley R, Dwyer DE., *Ischaemic heart disease, influenza and influenza vaccination: a prospective case control study.* 2013 **99**(24).
21. HPA, *Annual Influenza Report 2010/11* HPA, May 2011: http://www.hpa.org.uk/NewsCentre/NationalPressReleases/2011PressReleases/.
22. Turbelin C, S.C., Pelat C, Hanslik T, Sarazin M, Blanchon T, Falchi A., *Age distribution of influenza like illness cases during post-pandemic A(H3N2): comparison with the twelve previous seasons, in France.* PLoS One. , 2013 **8**(6).
23. Cromer D, v.H.A., Jit M, Edmunds WJ, Fleming D, Miller E., *The burden of influenza in England by age and clinical risk group: A statistical analysis to inform vaccine policy.* J Infect. , 2013.
24. *Common Terminology Criteria for Adverse Events*. 2010; v4.03: June 14, 2010:[Available from: http://evs.nci.nih.gov/ftp1/CTCAE/CTCAE_4.03_2010-06-14_QuickReference_8.5x11.pdf.

25. *Division of AIDS Table for Grading the Severity of Adult and Pediatric Adverse Events* 2009; Available from: http://rsc.tech-res.com/Document/safetyandpharmacovigilance/Table_for_Grading_Severity_of_Adult_Pediatric_Adverse_Events.pdf.

23. APPENDICES

Appendix 1: RVL Symptom Diary Card 95
Appendix 2: Wisconsin Symptom Diary Card 97
Appendix 3: Common Terminology Criteria for Adverse Events 98
Appendix 4: DAIDS Table for Grading the Severity of Adult and Pediatric Adverse Events 99

Appendix 1: RVL Symptom Diary Card

[Study No] RVL Symptom Diary Card [Version No]: [Version Date]

RVL Symptom Diary Card

RETROSCREEN VIROLOGY

Subject Number	Subject Initials	Date	Time (24 hour clock)	Time of Day (mark with an X)		
				Morning	Afternoon	Evening
	F M L	D D M M M Y Y Y Y	H H : M M			

Level	0	1	2	3
Symptoms<br>Please report the symptoms you are experiencing at the moment<br><br>(Mark with an ×)	I have NO symptoms	Just noticeable	It's clearly bothersome from time to time, but it doesn't stop me from participating in activities	It's quite bothersome most or all of the time, and it stops me from participating in activities
Runny Nose	☐ 0	☐ 1	☐ 2	☐ 3
Stuffy Nose	☐ 0	☐ 1	☐ 2	☐ 3
Sneezing	☐ 0	☐ 1	☐ 2	☐ 3
Sore Throat	☐ 0	☐ 1	☐ 2	☐ 3
Earache	☐ 0	☐ 1	☐ 2	☐ 3
Malaise (tiredness)	☐ 0	☐ 1	☐ 2	☐ 3
Cough	☐ 0	☐ 1	☐ 2	☐ 3
Shortness of breath	☐ 0	☐ 1	☐ 2	☐ 3
Headache	☐ 0	☐ 1	☐ 2	☐ 3
Muscle and/or joint ache	☐ 0	☐ 1	☐ 2	☐ 3

Subject's initials	Doctor's Initials	Date	Time (24 hour clock)
		D D M M M Y Y Y Y	H H : M M

[Study No] RVL Symptom Diary Card [Version No]: [Version Date]

RVL Symptom Diary Card

Subject Number	Subject Initials	Date	Time (24 hour clock)	Time of Day (mark with an X)		
	F M L	D D M M M Y Y Y Y	H H : M M	Morning	Afternoon	Evening

SYMPTOMS	Please report the symptoms you are experiencing at the moment<br>*Please mark the line with an X to indicate the severity of each of your symptoms*		
Runny Nose	Less severe ⇨	________	⇦ More severe
Stuffy Nose	Less severe ⇨	________	⇦ More severe
Sneezing	Less severe ⇨	________	⇦ More severe
Sore Throat	Less severe ⇨	________	⇦ More severe
Earache	Less severe ⇨	________	⇦ More severe
Malaise (tiredness)	Less severe ⇨	________	⇦ More severe
Cough	Less severe ⇨	________	⇦ More severe
Shortness of breath	Less severe ⇨	________	⇦ More severe
Headache	Less severe ⇨	________	⇦ More severe
Muscle and/or joint ache	Less severe ⇨	________	⇦ More severe

Subject's initials	Doctor's Initials	Date	Time (24 hour clock)
		D D M M M Y Y Y Y	H H : M M

Appendix 2: Wisconsin Symptom Diary Card

Wisconsin Upper Respiratory Symptom Survey – 21 --- *Daily Symptom Report*

Day:	*Date:*	*Time:*	*ID:*

Please fill in one circle for each of the following items:

	**Not sick** **0**	Very mildly **1**	**2**	Mildly **3**	**4**	Moderately **5**	**6**	Severely **7**
How sick do you feel **today**?	O	O	O	O	O	O	O	O

Please rate the **average severity of your cold symptoms over the last 24 hours** for each symptom:

	**Do not have this symptom** **0**	Very mild **1**	**2**	Mild **3**	**4**	Moderate **5**	**6**	Severe **7**
Runny nose	O	O	O	O	O	O	O	O
Plugged nose	O	O	O	O	O	O	O	O
Sneezing	O	O	O	O	O	O	O	O
Sore throat	O	O	O	O	O	O	O	O
Scratchy throat	O	O	O	O	O	O	O	O
Cough	O	O	O	O	O	O	O	O
Hoarseness	O	O	O	O	O	O	O	O
Head congestion	O	O	O	O	O	O	O	O
Chest congestion	O	O	O	O	O	O	O	O
Feeling tired	O	O	O	O	O	O	O	O

Over the last 24 hours, how much has your cold interfered with your ability to:

	**Not at all** **0**	Very mildly **1**	**2**	Mildly **3**	**4**	Moderately **5**	**6**	Severely **7**
Think clearly	O	O	O	O	O	O	O	O
Sleep well	O	O	O	O	O	O	O	O
Breathe easily	O	O	O	O	O	O	O	O
Walk, climb stairs, exercise	O	O	O	O	O	O	O	O
Accomplish daily activities	O	O	O	O	O	O	O	O
Work outside the home	O	O	O	O	O	O	O	O
Work inside the home	O	O	O	O	O	O	O	O
Interact with others	O	O	O	O	O	O	O	O
Live your personal life	O	O	O	O	O	O	O	O

Compared to yesterday, I feel that my cold is…

Very much better	Somewhat better	A little better	The same	A little worse	Somewhat worse	Very much worse
O	O	O	O	O	O	O

WURSS -21© (Wisconsin Upper Respiratory Symptom Survey) 2004
Created by Bruce Barrett MD PhD et al., UW Department of Family Medicine, 777 S. Mills St. Madison, WI 53715, USA

Appendix 3: Common Terminology Criteria for Adverse Events

Common Terminology Criteria for Adverse Events (CTCAE)

Version 4.0

Published: May 28, 2009 (v4.03: June 14, 2010)

U.S.DEPARTMENT OF HEALTH AND HUMAN SERVICES
National Institutes of Health
National Cancer Institute

Common Terminology Criteria for Adverse Events v4.0 (CTCAE)

Publish Date: May 28, 2009

Quick Reference

The NCI Common Terminology Criteria for Adverse Events is a descriptive terminology which can be utilized for Adverse Event (AE) reporting. A grading (severity) scale is provided for each AE term.

Components and Organization

SOC

System Organ Class, the highest level of the MedDRA hierarchy, is identified by anatomical or physiological system, etiology, or purpose (e.g., SOC Investigations for laboratory test results). CTCAE terms are grouped by MedDRA Primary SOCs. Within each SOC, AEs are listed and accompanied by descriptions of severity (Grade).

CTCAE Terms

An Adverse Event (AE) is any unfavorable and unintended sign (including an abnormal laboratory finding), symptom, or disease temporally associated with the use of a medical treatment or procedure that may or may *not* be considered related to the medical treatment or procedure. An AE is a term that is a unique representation of a specific event used for medical documentation and scientific analyses. Each CTCAE v4.0 term is a MedDRA LLT (Lowest Level Term).

Definitions

A brief definition is provided to clarify the meaning of each AE term.

Grades

Grade refers to the severity of the AE. The CTCAE displays Grades 1 through 5 with unique clinical descriptions of severity for each AE based on this general guideline:

Grade 1	Mild; asymptomatic or mild symptoms; clinical or diagnostic observations only; intervention not indicated.
Grade 2	Moderate; minimal, local or noninvasive intervention indicated; limiting age-appropriate instrumental ADL*.
Grade 3	Severe or medically significant but not immediately life-threatening; hospitalization or prolongation of hospitalization indicated; disabling; limiting self care ADL**.
Grade 4	Life-threatening consequences; urgent intervention indicated.
Grade 5	Death related to AE.

A Semi-colon indicates 'or' within the description of the grade.

A single dash (-) indicates a grade is not available.

Not all Grades are appropriate for all AEs. Therefore, some AEs are listed with fewer than five options for Grade selection.

Grade 5

Grade 5 (Death) is not appropriate for some AEs and therefore is not an option.

Activities of Daily Living (ADL)

*Instrumental ADL refer to preparing meals, shopping for groceries or clothes, using the telephone, managing money, etc.

**Self care ADL refer to bathing, dressing and undressing, feeding self, using the toilet, taking medications, and not bedridden.

+ CTCAE v4.0 incorporates certain elements of the MedDRA terminology. For further details on MedDRA refer to the MedDRA MSSO Web site (http://www.meddramsso.com).

The full list of CTCAE criteria is available at
http://evs.nci.nih.gov/ftp1/CTCAE/CTCAE_4.03_2010-06-14_QuickReference_8.5x11.pdf

Appendix 4: DAIDS Table for Grading the Severity of Adult and Pediatric Adverse Events

DIVISION OF AIDS TABLE FOR GRADING THE SEVERITY OF ADULT AND PEDIATRIC ADVERSE EVENTS
VERSION 1.0, DECEMBER, 2004; CLARIFICATION AUGUST 2009

The Division of AIDS Table for Grading the Severity of Adult and Pediatric Adverse Events ("DAIDS AE Grading Table") is a descriptive terminology which can be utilized for Adverse Event (AE) reporting. A grading (severity) scale is provided for each AE term.

This clarification of the DAIDS Table for Grading the Severity of Adult and Pediatric AE's provides additional explanation of the DAIDS AE Grading Table and clarifies some of the parameters.

I. Instructions and Clarifications

Grading Adult and Pediatric AEs

The DAIDS AE Grading Table includes parameters for grading both Adult and Pediatric AEs. When a single set of parameters is not appropriate for grading specific types of AEs for both Adult and Pediatric populations, separate sets of parameters for Adult and/or Pediatric populations (with specified respective age ranges) are given in the Table. If there is no distinction in the Table between Adult and Pediatric values for a type of AE, then the single set of parameters listed is to be used for grading the severity of both Adult and Pediatric events of that type.

> **Note:** In the classification of adverse events, the term "**severe**" is not the same as "**serious**." Severity is an indication of the intensity of a specific event (as in mild, moderate, or severe chest pain). The term "**serious**" relates to a participant/event outcome or action criteria, usually associated with events that pose a threat to a participant's life or functioning.

Addenda 1-3 Grading Tables for Microbicide Studies

For protocols involving topical application of products to the female genital tract, male genital area or rectum, strong consideration should be given to using Appendices I-III as the primary grading scales for these areas. The protocol would need to specifically state that one or more of the Appendices would be primary (and thus take precedence over the main Grading Table) for items that are listed in both the Appendix and the main Grading Table.

- Addendum 1 - Female Genital Grading Table for Use in Microbicide Studies - PDF
- Addendum 2 - Male Genital Grading Table for Use in Microbicide Studies - PDF
- Addendum 3 - Rectal Grading Table for Use in Microbicide Studies - PDF

Grade 5

For any AE where the outcome is death, the severity of the AE is classified as Grade 5.

Estimating Severity Grade for Parameters Not Identified in the Table

In order to grade a clinical AE that is not identified in the DAIDS AE grading table, use the category "Estimating Severity Grade" located on Page 3.

Determining Severity Grade for Parameters "Between Grades"

If the severity of a clinical AE could fall under either one of two grades (e.g., the severity of an AE could be either Grade 2 or Grade 3), select the higher of the two grades for the AE. If a laboratory value that is graded as a multiple of the ULN or LLN falls between two grades, select the higher of the two grades for the AE. For example, Grade 1 is 2.5 x ULN and Grade 2 is 2.6 x ULN for a parameter. If the lab value is 2.53 x ULN (which is between the two grades), the severity of this AE would be Grade 2, the higher of the two grades.

Values Below Grade 1

Any laboratory value that is between either the LLN or ULN and Grade 1 should not be graded.

Determining Severity Grade when Local Laboratory Normal Values Overlap with Grade 1 Ranges

In these situations, the severity grading is based on the ranges in the DAIDS AE Grading Table, even when there is a reference to the local lab LLN.

For example: Phosphate, Serum, Low, Adult and Pediatric > 14 years (Page 20) Grade 1 range is 2.[illegible] mg/dL - < LLN. A particular laboratory's normal range for Phosphate is 2.1 – 3.8 mg/dL. A participant[illegible] actual lab value is 2.5. In this case, the value of 2.5 exceeds the LLN for the local lab, but will be gra[illegible] as Grade 1 per DAIDS AE Grading Table.

.

II. Definitions of terms used in the Table:

Basic Self-care Functions	Adult<br>Activities such as bathing, dressing, toileting, transfer/movement, continence, and feeding.<br><br>Young Children<br>Activities that are age and culturally appropriate (e.g., feeding sel[illegible] culturally appropriate eating implement).
LLN	Lower limit of normal
Medical Intervention	Use of pharmacologic or biologic agent(s) for treatment of an AE.
NA	Not Applicable
Operative Intervention	Surgical OR other invasive mechanical procedures.
ULN	Upper limit of normal
Usual Social & Functional Activities	Adult<br>Adaptive tasks and desirable activities, such as going to work, shopping, cooking, use of transportation, pursuing a hobby, etc.<br><br>Young Children<br>Activities that are age and culturally appropriate (e.g., social interactions, play activities, learning tasks, etc.).

The full list of DAID criteria is available at: http://rsc.tech-res.com/Document/safetyandpharmacovigilance/Table_for_Grading_Severity_of_Adult_Pediatric_Adverse_Events.pdf.
